# Genetic Variation and Sickle Cell Disease Severity: A Systematic Review and Meta-Analysis

**DOI:** 10.1001/jamanetworkopen.2023.37484

**Published:** 2023-10-18

**Authors:** Justin K. Kirkham, Jeremie H. Estepp, Mitch J. Weiss, Sara R. Rashkin

**Affiliations:** 1 Department of Oncology, St Jude Children’s Research Hospital, Memphis, Tennessee; 2 Department of Hematology, St Jude Children’s Research Hospital, Memphis, Tennessee; 3 Department of Global Pediatric Medicine, St Jude Children’s Research Hospital, Memphis, Tennessee; 4 Now with Agios Pharmaceuticals, Cambridge, Massachusetts

## Abstract

**Importance:**

Sickle cell disease (SCD) is a monogenic disorder, yet clinical outcomes are influenced by additional genetic factors. Despite decades of research, the genetics of SCD remain poorly understood.

**Objective:**

To assess all reported genetic modifiers of SCD, evaluate the design of associated studies, and provide guidelines for future analyses according to modern genetic study recommendations.

**Data Sources:**

PubMed, Web of Science, and Scopus were searched through May 16, 2023, identifying 5290 publications.

**Study Selection:**

At least 2 reviewers identified 571 original, peer-reviewed English-language publications reporting genetic modifiers of human SCD phenotypes, wherein the outcome was not treatment response, and the comparison was not between SCD subtypes or including healthy controls.

**Data Extraction and Synthesis:**

Data relevant to all genetic modifiers of SCD were extracted, evaluated, and presented following STREGA and PRISMA guidelines. Weighted *z* score meta-analyses and pathway analyses were conducted.

**Main Outcomes and Measures:**

Outcomes were aggregated into 25 categories, grouped as acute complications, chronic conditions, hematologic parameters or biomarkers, and general or mixed measures of SCD severity.

**Results:**

The 571 included studies reported on 29 670 unique individuals (50% ≤ 18 years of age) from 43 countries. Of the 17 757 extracted results (4890 significant) in 1552 genes, 3675 results met the study criteria for meta-analysis: reported phenotype and genotype, association size and direction, variability measure, sample size, and statistical test. Only 173 results for 62 associations could be cross-study combined. The remaining associations could not be aggregated because they were only reported once or methods (eg, study design, reporting practice) and genotype or phenotype definitions were insufficiently harmonized. Gene variants regulating fetal hemoglobin and α-thalassemia (important markers for SCD severity) were frequently identified: 19 single-nucleotide variants in *BCL11A*, *HBS1L-MYB*, and *HBG2* were significantly associated with fetal hemoglobin (absolute value of Z = 4.00 to 20.66; *P* = 8.63 × 10^−95^ to 6.19 × 10^−5^), and α-thalassemia deletions were significantly associated with increased hemoglobin level and reduced risk of albuminuria, abnormal transcranial Doppler velocity, and stroke (absolute value of Z = 3.43 to 5.16; *P* = 2.42 × 10^−7^ to 6.00 × 10^−4^). However, other associations remain unconfirmed. Pathway analyses of significant genes highlighted the importance of cellular adhesion, inflammation, oxidative and toxic stress, and blood vessel regulation in SCD (23 of the top 25 Gene Ontology pathways involve these processes) and suggested future research areas.

**Conclusions and Relevance:**

The findings of this comprehensive systematic review and meta-analysis of all published genetic modifiers of SCD indicated that implementation of standardized phenotypes, statistical methods, and reporting practices should accelerate discovery and validation of genetic modifiers and development of clinically actionable genetic profiles.

## Key Points

**Question:**

What genetic modifiers of sickle cell disease (SCD) are currently defined, and what are potential approaches to improve future studies?

**Findings:**

In this systematic review and meta-analysis of 571 studies examining 29 670 individuals with SCD, 17 757 associations involving 1552 genes and 25 SCD phenotype categories were discovered; of these, only 173 associations met the study design, reporting, and phenotype or genotype harmonization required for meta-analysis. Gene variants regulating fetal hemoglobin and α-thalassemia were frequently identified, but other associations remained unconfirmed.

**Meaning:**

While major genetic modifiers of SCD severity were identified, including some that are clinically relevant, validated genetic associations were lacking, in part due to suboptimal study design and data reporting.

## Teaser

This systematic review and meta-analysis assesses all reported genetic modifiers of sickle cell disease and evaluates the design and data reporting of each study to provide guidelines for future analyses to accelerate discovery and validation of genetic modifiers in this disease.

## Introduction

Sickle cell disease (SCD) is the most common monogenic disorder in the world due to the protection that heterozygosity affords against malaria.^1^ Although SCD most heavily impacts sub-Saharan Africa, population migration and relocation have resulted in 1 in 2000 infants born in the United States with SCD, and 1 in 67 infants will be heterozygous carriers.^2,3,4^ Demographic trends and widespread improvements in clinical care will result in an increase in the proportion of the world’s population affected by SCD.^2^ An improved understanding of the pathophysiology of SCD and the environmental and genetic drivers of disease severity is essential to improve the lives of individuals with this disease.

Most cases of SCD are caused by a homozygous variation in the *HBB* gene (p.Glu6Val) encoding the β-globin subunit of adult hemoglobin tetramer (α2β2).^2^ At low oxygen concentrations in venous capillaries, sickle hemoglobin (α2β^S^2) forms rigid polymers, causing circulating red blood cells to become stiff, sticky, and brittle, triggering a complex pathophysiology including hemolysis, vascular occlusion, and inflammation.^2^ Clinical manifestations include severe acute and chronic pain, immunodeficiency, multiorgan damage, and early mortality. Hemolysis-related cellular injury, partly mediated by circulating free heme released from red blood cells, is thought to drive progression of cerebrovascular disease, kidney disease, pulmonary hypertension, priapism, and leg ulcers,^5^ whereas vaso-occlusion is thought to precipitate acute pain episodes, acute chest syndrome, and avascular necrosis.^2^

Despite being a monogenic disorder, the symptoms of SCD vary between affected individuals. The influence of environment on SCD is illustrated by markedly different outcomes between sub-Saharan Africa, where approximately half of affected children die before 5 years of age,^6^ and high-income countries, where enhanced medical support extends patient lifespan, although most patients still suffer considerably and die prematurely.^7^

Manifestations of SCD are also influenced by genetic factors. For example, residual expression of fetal hemoglobin (HbF, α2γ2) in postnatal red blood cells, which reduces SCD severity by interfering with polymerization of sickle hemoglobin,^8^ is largely determined genetically. Coinherited hereditary persistence of fetal hemoglobin, caused by variants in the extended β-like globin locus, results in extremely high levels of HbF, eliminating many symptoms of SCD.^9^ Genome-wide association studies have shown that 20%-50% of the variation in HbF can be explained by single-nucleotide variants (SNVs) in 3 loci: *BCL11A*, *HBS1L*-*MYB*, and the extended β-like globin locus.^10,11,12,13^ The erythroid-specific enhancer *BCL11A* encodes a potent transcriptional repressor for the γ-globin genes (*HBG1* and *HBG2*).^10,11,14^ This discovery led to gene therapy strategies aimed at reducing erythroid *BCL11A* expression, some of which are showing early signs of efficacy in clinical trials,^15,16^ illustrating how understanding the genetic modifiers of SCD can have profound therapeutic implications.

The genetic contributions to SCD-related complications are poorly defined, despite a preponderance of publications on this topic. As a motivating example, a recent polygenic score incorporating 21 SNVs in 9 genetic loci, including HbF modifiers, explained only 3.5% of the variation in acute pain episodes.^17^ A more complete understanding of how genetics influences pathophysiology could improve therapy by providing tools to predict outcomes and identifying new modes for therapeutic intervention. To assess current knowledge, we performed a systematic review of, to our knowledge, all publications reporting genetic modifiers of SCD, cataloged the findings by subdividing genotype-phenotype associations by quality of data analysis and reporting, and performed meta-analyses and pathway analyses. Based on our findings and current guidelines in the field of human genetics, we provide recommendations for analytical approaches and reporting to enhance scientific rigor, reduce spurious results, and facilitate cross-study data synthesis.

## Methods

### Article Search and Abstract Screening

This systematic review was prospectively registered with PROSPERO (No. CRD42021274466) and was reported following the Preferred Reporting Items for Systematic Reviews and Meta-analyses (PRISMA) reporting guideline and the Strengthening the Reporting of Genetic Association Studies (STREGA) reporting guideline. We searched PubMed, Web of Science, and Scopus for all studies reporting genetic modifiers of SCD, irrespective of SCD subtype, published before May 16, 2023 (search terms in eMethods in Supplement 1). A total of 8892 studies were identified (eFigure 1 in Supplement 1): 3132 from PubMed, 2443 from Web of Science, and 3317 from Scopus. After deduplication, 5290 unique manuscripts remained.

Abstracts were screened by 2 independent reviewers (J.K.K., S.R.R.), with a third reviewer (J.H.E.) blinded to other screening opinions resolving disagreements. To comprehensively aggregate all published mutations associated with SCD-related outcomes and to avoid excluding important genetic modifiers due to incorrect phenotypic or genotypic attribution, we included all reported phenotypes and genetic polymorphisms. Studies were excluded if the manuscript was unavailable in English, the research was not conducted in humans, individuals without SCD were included, the only analysis was nongenetic, the only comparison was between SCD subtypes, the outcome was treatment response only, the manuscript was not peer reviewed, or no original research was included. A total of 571 publications passed this screening (eFigure 1 in Supplement 1, eTable 1 in Supplement 2).^8,11,12,13,17,18,19,20,21,22,23,24,25,26,27,28,29,30,31,32,33,34,35,36,37,38,39,40,41,42,43,44,45,46,47,48,49,50,51,52,53,54,55,56,57,58,59,60,61,62,63,64,65,66,67,68,69,70,71,72,73,74,75,76,77,78,79,80,81,82,83,84,85,86,87,88,89,90,91,92,93,94,95,96,97,98,99,100,101,102,103,104,105,106,107,108,109,110,111,112,113,114,115,116,117,118,119,120,121,122,123,124,125,126,127,128,129,130,131,132,133,134,135,136,137,138,139,140,141,142,143,144,145,146,147,148,149,150,151,152,153,154,155,156,157,158,159,160,161,162,163,164,165,166,167,168,169,170,171,172,173,174,175,176,177,178,179,180,181,182,183,184,185,186,187,188,189,190,191,192,193,194,195,196,197,198,199,200,201,202,203,204,205,206,207,208,209,210,211,212,213,214,215,216,217,218,219,220,221,222,223,224,225,226,227,228,229,230,231,232,233,234,235,236,237,238,239,240,241,242,243,244,245,246,247,248,249,250,251,252,253,254,255,256,257,258,259,260,261,262,263,264,265,266,267,268,269,270,271,272,273,274,275,276,277,278,279,280,281,282,283,284,285,286,287,288,289,290,291,292,293,294,295,296,297,298,299,300,301,302,303,304,305,306,307,308,309,310,311,312,313,314,315,316,317,318,319,320,321,322,323,324,325,326,327,328,329,330,331,332,333,334,335,336,337,338,339,340,341,342,343,344,345,346,347,348,349,350,351,352,353,354,355,356,357,358,359,360,361,362,363,364,365,366,367,368,369,370,371,372,373,374,375,376,377,378,379,380,381,382,383,384,385,386,387,388,389,390,391,392,393,394,395,396,397,398,399,400,401,402,403,404,405,406,407,408,409,410,411,412,413,414,415,416,417,418,419,420,421,422,423,424,425,426,427,428,429,430,431,432,433,434,435,436,437,438,439,440,441,442,443,444,445,446,447,448,449,450,451,452,453,454,455,456,457,458,459,460,461,462,463,464,465,466,467,468,469,470,471,472,473,474,475,476,477,478,479,480,481,482,483,484,485,486,487,488,489,490,491,492,493,494,495,496,497,498,499,500,501,502,503,504,505,506,507,508,509,510,511,512,513,514,515,516,517,518,519,520,521,522,523,524,525,526,527,528,529,530,531,532,533,534,535,536,537,538,539,540,541,542,543,544,545,546,547,548,549,550,551,552,553,554,555,556,557,558,559,560,561,562,563,564,565,566,567,568,569,570,571,572,573,574,575,576,577,578,579,580,581,582,583^ Data from these studies were extracted (eMethods, eTable 2, and eTable 3 in Supplement 2). Following extraction, we standardized gene annotation and phenotype categories to facilitate results tabulation (eMethods, eTable 4 in Supplement 1). No individual-level participant data were used.

### Risk of Bias

**Figure 1.**
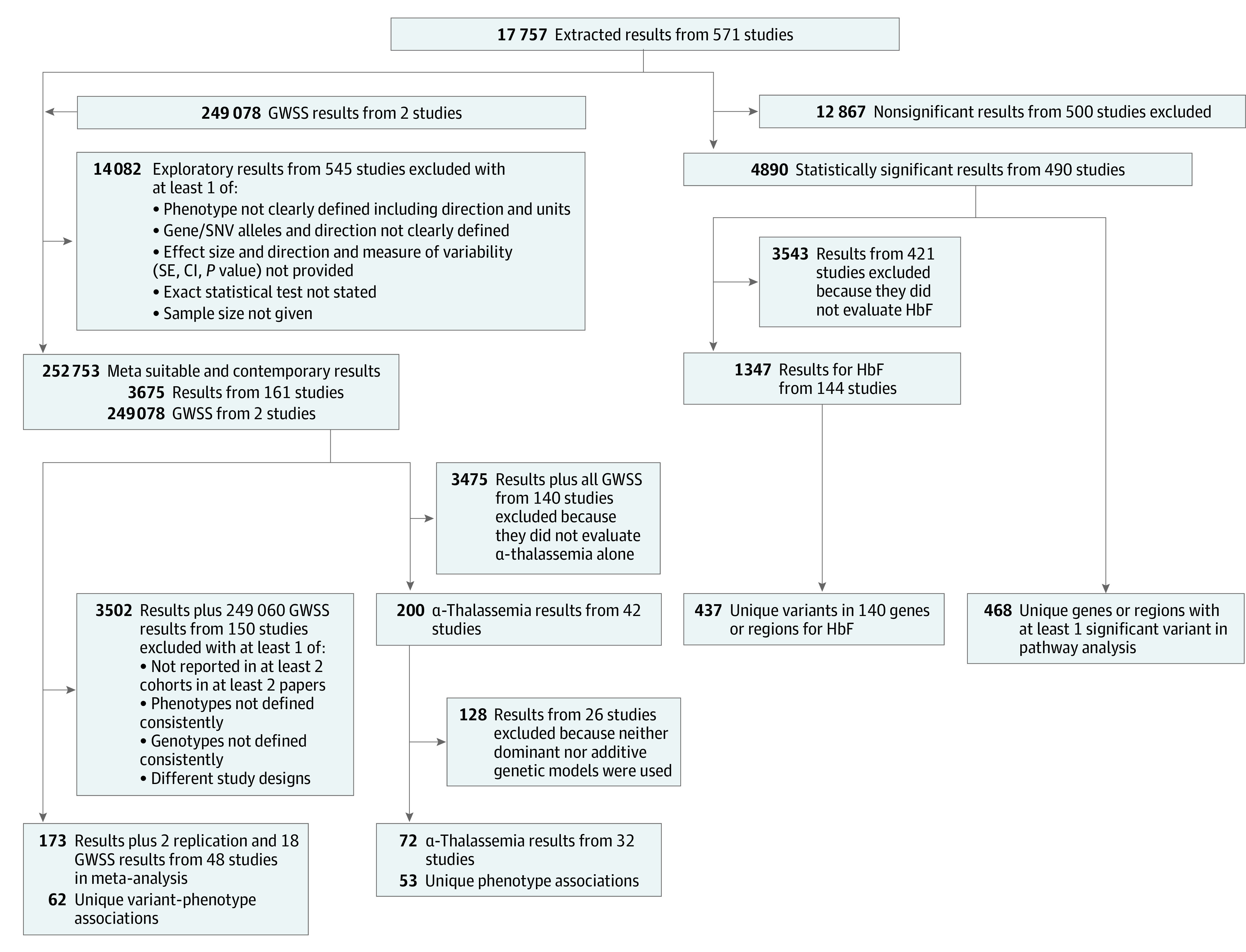
Flowchart of Analysis GWSS indicates genome-wide summary statistics; HbF, fetal hemoglobin.

Evolving approaches to genetic studies and clinical care over time, combined with variability in study design, phenotype definitions, and reporting practices, resulted in highly heterogeneous data, even within a single publication. Rather than determining the risk of bias for each publication, we created 3 categories into which all results were assigned using the STREGA guidelines^584^ (eMethods in Supplement 1). Briefly, *exploratory* results were evaluated statistically but lacked information required for cross-study meta-analysis. *Meta-suitable* results contained the minimum information to allow for meta-analysis: clearly defined outcome and genetic variants, sample size, statistical test, association size, direction, and measure of variability. *Contemporary* results contained all requirements for the meta-suitable category plus further elements crucial for genetic association studies (ie, quality control checks, accounting for population stratification and relatedness, covariate adjustment, and external validation). We did not exclude results based on these categories; however, some sections only used meta-suitable and contemporary results (Figure 1; eMethods in Supplement 1).

### Meta-Analysis

We conducted meta-analyses using a weighted *z* score–based approach (eMethods in Supplement 1) on all SNV-phenotype pairs with meta-suitable and contemporary results from at least 2 cohorts reported in at least 2 manuscripts in which the phenotype was the same, the same genotype comparison was done, and the same statistical test was performed. When a manuscript reported multiple results for the same cohort, we selected the one most similar to the other results being used in terms of adjustment for other covariates. If multiple studies reported results for the same cohort, we selected the result using the largest sample size.

### Beyond Meta-Analysis

Our meta-analyses included only associations in which phenotypes and genotypes were defined consistently across studies. While statistically rigorous, this approach omitted biologically relevant associations established through repeated linking of loci to related phenotypes. As variability in study design and reporting prevented meta-analysis of most results, including well-established modifiers, we performed further data interrogations, as described here and in the eMethods in Supplement 1.

We examined genes with variants significantly associated with HbF in at least 3 manuscripts because HbF is a well-known disease modifier.^8^ Similarly, coinherited deletional α-thalassemia is common in SCD populations and modifies SCD pathophysiology,^484^ although the association with SCD varies across phenotypes and studies.^2^ To illustrate this comprehensively, we compared all meta-suitable and contemporary associations of SCD phenotypes with α-thalassemia deletions (eMethods in Supplement 1). Finally, we conducted pathway analyses to align significant findings with biological functions from the curated Gene Ontology (GO) and Reactome databases (eMethods in Supplement 1). This approach is used to analyze lists of important genes to facilitate interpretation and hypothesis generation.

### Statistical Analysis

Unless stated otherwise, analyses were conducted with R, version 4.2.1 (CRAN). For the meta-analyses, a Bonferroni-corrected 2-sided *P* < 8.1 × 10^−4^ (.05/62) was considered statistically significant. For pathway analyses, an adjusted *P* < .05 was considered significant.

## Results

We identified 571 manuscripts published before May 16, 2023, reporting genotype-phenotype associations across 29 670 unique individuals (50% ≤ 18 years of age) from 43 countries (eMethods, eFigures 1 and 2, and eTable 5 in Supplement 1; eTable 1 in Supplement 2).^8,11,12,13,17,18,19,20,21,22,23,24,25,26,27,28,29,30,31,32,33,34,35,36,37,38,39,40,41,42,43,44,45,46,47,48,49,50,51,52,53,54,55,56,57,58,59,60,61,62,63,64,65,66,67,68,69,70,71,72,73,74,75,76,77,78,79,80,81,82,83,84,85,86,87,88,89,90,91,92,93,94,95,96,97,98,99,100,101,102,103,104,105,106,107,108,109,110,111,112,113,114,115,116,117,118,119,120,121,122,123,124,125,126,127,128,129,130,131,132,133,134,135,136,137,138,139,140,141,142,143,144,145,146,147,148,149,150,151,152,153,154,155,156,157,158,159,160,161,162,163,164,165,166,167,168,169,170,171,172,173,174,175,176,177,178,179,180,181,182,183,184,185,186,187,188,189,190,191,192,193,194,195,196,197,198,199,200,201,202,203,204,205,206,207,208,209,210,211,212,213,214,215,216,217,218,219,220,221,222,223,224,225,226,227,228,229,230,231,232,233,234,235,236,237,238,239,240,241,242,243,244,245,246,247,248,249,250,251,252,253,254,255,256,257,258,259,260,261,262,263,264,265,266,267,268,269,270,271,272,273,274,275,276,277,278,279,280,281,282,283,284,285,286,287,288,289,290,291,292,293,294,295,296,297,298,299,300,301,302,303,304,305,306,307,308,309,310,311,312,313,314,315,316,317,318,319,320,321,322,323,324,325,326,327,328,329,330,331,332,333,334,335,336,337,338,339,340,341,342,343,344,345,346,347,348,349,350,351,352,353,354,355,356,357,358,359,360,361,362,363,364,365,366,367,368,369,370,371,372,373,374,375,376,377,378,379,380,381,382,383,384,385,386,387,388,389,390,391,392,393,394,395,396,397,398,399,400,401,402,403,404,405,406,407,408,409,410,411,412,413,414,415,416,417,418,419,420,421,422,423,424,425,426,427,428,429,430,431,432,433,434,435,436,437,438,439,440,441,442,443,444,445,446,447,448,449,450,451,452,453,454,455,456,457,458,459,460,461,462,463,464,465,466,467,468,469,470,471,472,473,474,475,476,477,478,479,480,481,482,483,484,485,486,487,488,489,490,491,492,493,494,495,496,497,498,499,500,501,502,503,504,505,506,507,508,509,510,511,512,513,514,515,516,517,518,519,520,521,522,523,524,525,526,527,528,529,530,531,532,533,534,535,536,537,538,539,540,541,542,543,544,545,546,547,548,549,550,551,552,553,554,555,556,557,558,559,560,561,562,563,564,565,566,567,568,569,570,571,572,573,574,575,576,577,578,579,580,581,582,583^ Approximately 52% of individuals resided in USA, Canada, France, or Brazil, while just 6628 individuals studied (22%) were from African cohorts (eFigure 2 and eTable 5 in Supplement 1). Fifty-five manuscripts (10%) assessed individuals from more than 1 country. At least 14 970 individuals were included in studies as children (eTable 1 in Supplement 2 and eTable 5 in Supplement 1), but some children were also included in studies as adults in longitudinal cohorts.

### Risk of Bias

Of 17 757 reported associations, 3631 (20%) were meta-suitable, containing all elements required to perform a meta-analysis, and only 44 results from 2 studies met all contemporary criteria (eTable 2 in Supplement 2). The proportion of studies using a more rudimentary exploratory approach to a more rigorous meta-suitable approach did not appear to change over time (eFigure 3 in Supplement 1). For the rest of our analysis, we grouped the contemporary results with the meta-suitable results (3675 total results).

### Genotype-Phenotype Associations

Across the 571 publications analyzed, 17 757 association results in 1552 unique genes were reported, along with 249 078 genome-wide association summary statistics (Figure 1, Table 1, and eTables 2 and 3 in Supplement 2). The number of genes interrogated varied by phenotype category (Table 1), ranging from 9 (retinopathy) to 452 (HbF). Of the 2399 unique gene- or polygene-phenotype category pairs (eTable 6 in Supplement 2), there was a median (IQR) of 2 (1-4) results, with 1976 (82%) limited to a single study. Overall, 4890 (28%) extracted results were statistically significant (eMethods in Supplement 1), but this does not account for varying significance thresholds (ie, 0.05, 5 × 10^−8^, etc).

**Table 1.** Number Of Studies, Total Results, and Unique Genes Reported for Each Phenotype Category

Phenotype category^a^	Complication prevalence, %^b^	Total studies, No.	Total results, No.	Unique genes, No.
Acute SCD-related complications			2316	
Acute pain episode	100	140	1467	113
ACS, pneumonia, or respiratory infection	30	74	334	61
Infection (excludes respiratory infection)	10	42	290	35
Priapism	30^c^	32	108	40
Acute splenic sequestration	15	19	51	11
Other acute phenotype	NA	24	66	17
Chronic SCD-related complications			6253	
Allo- or autoantibody or transfusion reaction	20	14	1067	253
Cerebrovascular disease	50	106	1083	222
Kidney dysfunction	35	57	1174	220
Cardiopulmonary dysfunction	50	37	606	83
Hyperbilirubinemia, cholelithiasis, cholecystitis, or cholecystectomy	50	102	815	46
Osteonecrosis	30	59	227	45
Leg ulcers	15	42	163	43
Iron overload	30	30	81	29
Chronic pain	55	13	225	22
Splenic dysfunction	90	28	60	11
Retinopathy	50	13	52	9
Other chronic phenotype	NA	37	700	190
Hematologic parameters and biomarkers of disease severity			8582	
HbF^d^	NA	240	3952	452
Hemolysis	NA	155	789	64
Anemia^d^	95	196	738	58
Oxidative stress	NA	23	196	22
Other hematologic parameter	NA	199	2345	113
Other parameter or biomarker	NA	63	562	78
General or mixed measurement of SCD severity	NA	89	606	171
Total^d^	NA	571	17 757	1552

Abbreviations: ACS, acute chest syndrome; HbF, fetal hemoglobin; NA, not applicable; SCD, sickle cell disease.

^a^ Within each subset, phenotype categories are ordered by decreasing number of total unique genes, excepting “other” categories, which are listed last.

^b^ Complication prevalence rates were obtained from published estimates among adults of all SCD subtypes within the United States, when available (eMethods in Supplement 1).

^c^ Among male participants.

^d^ Excludes 249 078 genome-wide summary statistics from 2 publications to avoid count distortion.

### Meta-Analysis

While 3675 of the 17 757 total results (21%) were categorized as meta-suitable, due to differences in specific phenotypes, genotypes, or statistical methods, studies analyzing similar outcomes were often insufficiently harmonized for cross-comparison or were not replicated (Figure 1; eMethods in Supplement 1). Only 173 of 17 757 results (1%) plus 2 replication results and 18 genome-wide association summary statistics, representing 62 distinct genotype-phenotype associations, could be cross-study meta-analyzed (Figure 1, Table 2). Of these 193 results, 111 (58%) matched direction and significance with the meta-analysis results; of the remaining 82, 54 (66%) were in directional agreement but differed in statistical significance.

**Table 2.** Meta-Analysis Results^a^

Phenotype category	Specific outcome	Variant	Gene	EA	OA	No. of studies^b^	Total sample size, No.	*z* Score^c^	*P* value^d^	Direction^e^	Significance^f^
ACS, pneumonia, or respiratory infection^189,363^	ACS (event occurrence)	rs2070744	*NOS3*	CC	TT or CT	2	273	1.4	.16	+−	SN
Acute pain episode^148,207,300^	VOC (event occurrence)	α-Thalassemia	α-Globin	1 or 2 Deletions	No deletions	3	435	2.18	.03	+++	NNN
Acute pain episode^185,333^	VOC (event occurrence)	rs5030737, rs1800450, rs1800451	*MBL2*	AA	AO or OO	2	155	−0.62	.54	+−	NS
Acute pain episode^17,92^	VOC (event rate)	rs1042713	*ADRB2*	A	G	2	463	−1.16	.25	−−	NN
Acute pain episode^17,92^	VOC (event rate)	rs1042713	*ADRB2*	AA or AG	GG	2	463	−2.55	.01	−−	SN
Acute pain episode^17,113^	VOC (event rate)	rs10483639	*GCH1*	C	G	2	458	1.96	.05	++	NN
Acute pain episode^17,113^	VOC (event rate)	rs10483639	*GCH1*	CC or CG	GG	2	458	1.61	.11	+−	NN
Acute pain episode^17,159^	VOC (event rate)	rs1800587	*IL1A*	A	G	2	442	2.92	.004	+−	SN
Acute pain episode^17,159^	VOC (event rate)	rs1800587	*IL1A*	AA or AG	GG	2	442	2.19	.03	+−	NN
Acute pain episode^17,116^	VOC (event rate)	rs1947913	*TRPA1*	A	T	2	459	−1.67	.10	−+	NN
Acute pain episode^17,116^	VOC (event rate)	rs1947913	*TRPA1*	AA or AT	TT	2	459	0.017	.99	−+	NN
Acute pain episode^17,112^	VOC (event rate)	rs2963155	*NR3C1*	A	G	2	463	0.87	.38	+−	SS
Acute pain episode^17,112^	VOC (event rate)	rs2963155	*NR3C1*	AA	GG or GA	2	463	0.71	.48	+−	SS
Acute pain episode^17,227^	VOC (event rate)	rs4680	*COMT*	A	G	2	457	3.3	9.60 × 10^−4^	++	SN
Acute pain episode^17,265^	VOC (event rate)	rs6858735	*TBC1D1*	T	C	3	2228	4.57	4.81 × 10^−6^	++−	SNN
Acute pain episode^17,265^	VOC (event rate)	rs7899453	*RPS24*	A	C	3	2228	4.7	2.61 × 10^−6^	+++	NNN
Acute splenic sequestration^148,300^	Acute splenic sequestration (event occurrence)	α-Thalassemia	α-Globin	1 or 2 Deletions	No deletions	2	239	1.59	.11	−+	NS
Anemia^148,207^	Blood transfusion (event occurrence)	α-Thalassemia	α-Globin	1 or 2 Deletions	No deletions	2	225	−1.81	.07	−−	NN
Anemia^118,436^	Hemoglobin (continuous)	α-Thalassemia	α-Globin	1 or 2 Deletions	No deletions	2	1193	5.12	3.11 × 10^−7^	++	SS
Anemia^115,223^	Hemoglobin (continuous)	rs66650371	*HBS1L-MYB*	D	I	2	986	2.96	.003	++	SN
Anemia^115,223^	Hemoglobin (continuous)	rs7482144	Extended β-globin locus	A	G	2	986	2.4	.02	++	NS
Cerebrovascular disease^25,389^	Abnormal TCD result (event occurrence)	α-Thalassemia	α-Globin	1 or 2 Deletions	No deletions	2	366	−5.16	2.42 × 10^−7^	−−	SS
Cerebrovascular disease^21,118,148,300,311,445^	Stroke (event occurrence)	α-Thalassemia	α-Globin	1 or 2 Deletions	No deletions	6	3655	−5.12	2.97 × 10^−7^	−−−+−−	SSNNSS
Cerebrovascular disease^382,400^	Stroke (event occurrence)	GT repeats	*AGT*	A3 and/or A4	Other	2	219	−0.35	0.73	−+	NS
Cerebrovascular disease^300,387^	Stroke (event occurrence)	rs1800629	*TNF*	AA or GA	GG	3	599	−1.83	.07	−−−	NSN
Cerebrovascular disease^196,224^	Stroke (time to event)	α-Thalassemia	α-Globin	1 or 2 Deletions	No deletions	2	595	−3.43	6.00 × 10^−4^	−−	SS
Hyperbilirubinemia, cholelithiasis, cholecystitis, or cholecystectomy^148,289^	Cholelithiasis (event occurrence)	α-Thalassemia	α-Globin	1 or 2 Deletions	No deletions	2	242	−2.48	.01	−−	NS
Hyperbilirubinemia, cholelithiasis, cholecystitis, or cholecystectomy^344,380,205^	Cholelithiasis (event occurrence)	TA repeats	*UGT1A* locus	(6/6)	(7/7)	4	821	−5.09	3.57 × 10^−7^	−−−−	NSSS
Hyperbilirubinemia, cholelithiasis, cholecystitis, or cholecystectomy^344,380^	Cholelithiasis (event occurrence)	TA repeats	*UGT1A* locus	(6/6)	(6/7)	3	719	−1.92	.06	−−−	NNN
Hyperbilirubinemia, cholelithiasis, cholecystitis, or cholecystectomy^380,205^	Cholelithiasis (event occurrence)	TA repeats	*UGT1A* locus	(6/6)	(7/8)	3	668	−4.27	1.93 × 10^−5^	−−−	NNS
General or mixed measurement of SCD severity^148,207^	Hospitalization (event occurrence)	α-Thalassemia	α-Globin	1 or 2 Deletions	No deletions	2	225	−0.9	.37	−−	NN
HbF^17,57,163,269,270,312^	HbF (continuous)	rs11886868	*BCL11A*	T	C	7	2339	−15.3	7.08 × 10^−53^	−−−−−−−	SSNNSSS
HbF^17,115,122,163,202,214,269,270^	HbF (continuous)	rs1427407	*BCL11A*	T	G	10	3394	20.66	8.63 × 10^−95^	++++++++++	SSSSSSSSNS
HbF^17,228,312^	HbF (continuous)	rs28384513	*HBS1L-MYB*	A	C	4	1947	5.26	1.43 × 10^−7^	++−+	NNNS
HbF^17,210^	HbF (continuous)	rs35786788	*HBS1L-MYB*	A	G	2	1606	7.11	1.20 × 10^−12^	++	NN
HbF^17,163,228,269,270,312^	HbF (continuous)	rs4671393	*BCL11A*	A	G	7	2256	16.25	2.19 × 10^−59^	+++++++	SSSNSSS
HbF^17,57,210,228,269,270^	HbF (continuous)	rs4895441	*HBS1L-MYB*	A	G	6	2221	−7.55	4.24 × 10^−14^	−−−−+−	SSNSNN
HbF^115,122^	HbF (continuous)	rs6545816	*BCL11A*	A	C	2	841	−4	6.19 × 10^−5^	+−	NS
HbF^17,115,122,210^	HbF (continuous)	rs66650371	*HBS1L-MYB*	D	I	4	2447	9.52	1.82 × 10^−21^	++++	NSSS
HbF^17,214^	HbF (continuous)	rs6706648	*BCL11A*	T	C	5	1728	−12.57	3.01 × 10^−36^	−−−−−	SNSNS
HbF^269,270^	HbF (continuous)	rs6729815	*BCL11A*	T	C	2	198	−0.12	.91	−+	NN
HbF^17,269,270^	HbF (continuous)	rs6732518	*BCL11A*	T	C	3	782	−4.55	5.27 × 10^−6^	−++	SNN
HbF^17,214^	HbF (continuous)	rs6738440	*BCL11A*	A	G	5	1728	9.7	3.10 × 10^−22^	+++++	SNSNS
HbF^269,270^	HbF (continuous)	rs73555746	*HBS1L-MYB*	A	C	2	198	0.39	.69	+−	NN
HbF^17,115,122,228,312^	HbF (continuous)	rs7482144	Extended β-globin locus	A	G	5	2637	7.14	9.27 × 10^−13^	+++++	NNSSS
HbF^17,57^	HbF (continuous)	rs7557939	*BCL11A*	A	G	2	834	−9.19	3.95 × 10^−20^	−−	SN
HbF^57,214^	HbF (continuous)	rs7599488	*BCL11A*	T	C	4	1298	0.47	.63	−++−	NNNN
HbF^17,202,214^	HbF (continuous)	rs7606173	*BCL11A*	C	G	6	2354	−13.68	1.41 × 10^−42^	−−+−−−	SSNSNS
HbF^17,269,270^	HbF (continuous)	rs766432	*BCL11A*	A	C	3	782	−10.13	4.04 × 10^−24^	−+−	SNS
HbF^269,270^	HbF (continuous)	rs7775698	*HBS1L-MYB*	A	G	2	198	0.53	0.60	+−	NN
HbF^17,163,228,269,270,312^	HbF (continuous)	rs9399137	*HBS1L-MYB*	T	C	7	2256	−7.24	4.64 × 10^−13^	−−−−−−−	NSNNNSS
HbF^17,115,163,269,270,312^	HbF (continuous)	rs9402686	*HBS1L-MYB*	A	G	7	2349	8.44	3.11 × 10^−17^	+++−+++	SSSNNSS
HbF^17,312^	HbF (continuous)	rs9494142	*HBS1L-MYB*	T	C	3	1780	−5.31	1.07 × 10^−7^	−−−	SNN
HbF^17,57,210^	HbF (continuous)	rs9494145	*HBS1L-MYB*	T	C	3	1856	−7.88	3.33 × 10^−15^	−−−	NSN
Leg ulcers^148,118^	Leg ulcers (event occurrence)	α-Thalassemia	α-Globin	1 or 2 Deletions	No deletions	2	2336	−2.05	.04	−−	NN
Other hematologic parameter^115,223^	PLT (continuous)	rs66650371	*HBS1L-MYB*	D	I	2	986	−0.96	.34	−+	SN
Other hematologic parameter^115,223^	PLT (continuous)	rs7482144	Extended β-globin locus	A	G	2	986	−1.97	.05	−−	NN
Other hematologic parameter^115,223^	RBC count (continuous)	rs66650371	*HBS1L-MYB*	D	I	2	986	1.89	.06	++	SN
Other hematologic parameter^115,223^	RBC count (continuous)	rs7482144	Extended β-globin locus	A	G	2	986	1.66	.10	++	NN
Priapism^21,118,148^	Priapism (event occurrence)	α-Thalassemia	α-Globin	1 or 2 Deletions	No deletions	3	1745	−2.64	.008	−−+	SNN
Kidney dysfunction^20,320,514^	Albuminuria (time to event)	α-Thalassemia	α-Globin	1 or 2 Deletions	No deletions	3	978	−3.54	4.10 × 10^−4^	−−−	NSS
Kidney dysfunction^170,543^	Albuminuria (event occurrence)	G1/G2	*APOL1*	Homozygous G1 or G2 or compound heterozygous	Other	2	433	2.83	.005	++	NS

Abbreviations: ACS, acute chest syndrome; EA, effect allele or genotype; HbF, fetal hemoglobin; OA, other allele or genotype; PLT, platelets; RBC, red blood cell; TCD, transcranial doppler; VOC, vaso-occlusive crisis.

^a^ Weighted *z* score meta-analyses were conducted for all single-nucleotide variant–phenotype pairs with meta-suitable or contemporary results from at least 2 cohorts that were reported in at least 2 manuscripts, where the phenotype was the same, the same genotype comparison was made, and the same statistical test was performed.

^b^ Represents the number of results combined in each meta-analysis, including replication results and genome-wide summary statistics.

^c^ Meta-analysis *z* score, indicating direction of association for EA.

^d^ Statistically significant at the Bonferroni-corrected threshold of *P* < 8.1 × 10^−4^ (.05/62).

^e^ Represents the association direction for the EA compared with the OA of each component study: + indicates increasing and −, decreasing.

^f^ Significance for each respective association is labeled S when it was reported as significant and N otherwise.

Meta-analysis results indicated that α-thalassemia deletions were significantly associated with increased hemoglobin level (*Z* = 5.12; *P* = 3.11 × 10^−7^) and reduced risk of albuminuria (*Z* = −3.54; *P* = 4.10 × 10^−4^), abnormal transcranial Doppler velocity (*Z* = −5.16; *P* = 2.42 × 10^−7^), and stroke (*Z* = −5.12; *P* = 2.97 × 10^−7^ for occurrence; *Z* = −3.43; *P* = 6.00 × 10^−4^ for time to event) (Table 2). Ten SNVs in *BCL11A*, 8 in *HBS1L-MYB*, and 1 in the γ-globin gene (rs7482144, the XmnI site of *HBG2*) were significantly associated with HbF (absolute value of *Z* = 4.00 to 20.66; *P* = 8.63 × 10^−95^ to 6.19 × 10^−5^). An increased number *UGT1A1* promoter repeats was associated with increased risk for cholelithiasis (*Z* = 5.09, *P* = 3.57 × 10^−7^ for (TA)7/(TA)7; *Z* = 4.27, *P* = 1.93 × 10^−5^ for (TA)7/(TA)8; compared with (TA)6/(TA)6). Single-nucleotide variants in *RPS24* (rs7899453-A, *Z* = 4.70; *P* = 2.61 × 10^−6^) and *TBC1D1* (rs6858735-T, *Z* = 4.57; *P* = 4.81 × 10^−6^) were significantly associated with increased rate of vaso-occlusive crisis.

While high-risk G1/G2 *APOL1* variants were frequently associated with numerous markers for kidney dysfunction (eTables 2 and 6 in Supplement 2), only 2 results could be meta-analyzed, showing nominal association with increased risk for albuminuria (*Z* = 2.83, *P* = .005) (Table 2). Similarly, 1 SNV in *COMT* (rs4680) was nominally associated with vaso-occlusive crisis in our meta-analyses (*Z* = 3.30, *P* = 9.60 × 10^−4^) (Table 2), but numerous SNVs and haplotypes within this gene have been associated with acute pain outcomes (eTables 2 and 6 in Supplement 2).

### HbF

The genetics of HbF expression was more widely studied than any other SCD phenotype, with 240 of 571 studies (42%) reporting a total of 3952 associations involving 452 genes (Table 1; eTables 2 and 6 in Supplement 2). Significant associations with HbF were reported for 140 genes in 144 studies, yet 1220 of 1347 (91%) of these associations were exploratory (eFigure 4 in Supplement 1 and eTables 2 and 6 in Supplement 2). Most significant results identified the extended β-like globin locus, *BCL11A*, or *HBS1L-MYB*, with few repeated or meta-suitable results outside of these regions (eFigure 4 in Supplement 1). Numerous studies linked these same HbF modifier genes to specific complications of SCD, including acute pain, anemia, cerebrovascular disease, and hemolysis (eTables 2 and 6 in Supplement 2).

### α-Thalassemia

**Figure 2.**
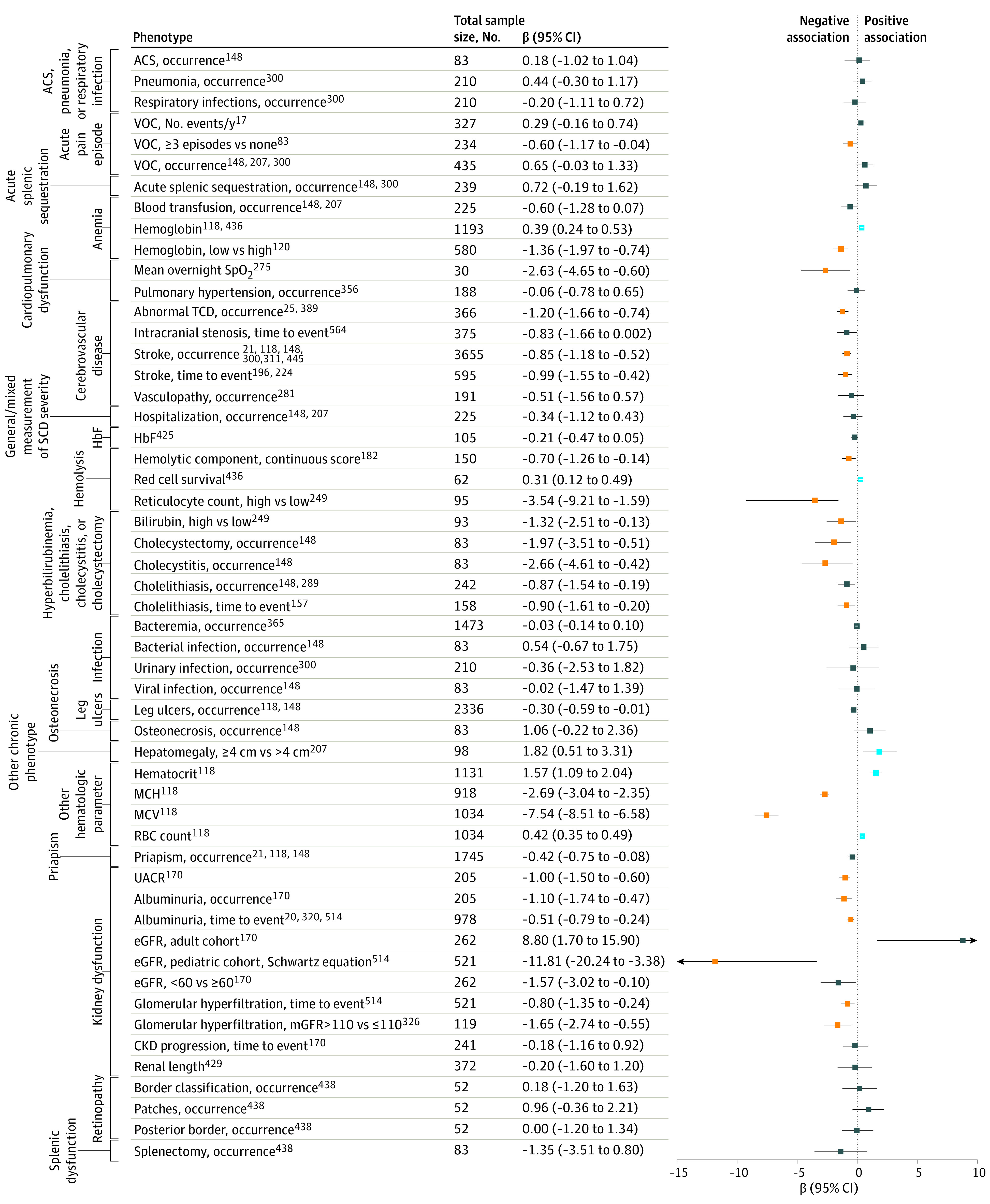
Published α-Thalassemia Associations Across All Meta-Suitable Results All associations are comparing 1 or 2 deletions vs no deletions, except fetal hemoglobin (HbF) and cholelithiasis (time to event), where only additive genotype coding was reported. Where appropriate, odds ratios and hazard ratios were transformed to β-scale (log odds ratio or log hazard ratio) for purposes of plotting. Results studied in more than 1 study were first combined via fixed-effects meta-analysis. Significant results as originally reported are shown in blue and gold for positive and negative association directions, respectively. For those meta-analyzed, significance was determined as *P* < 8.1 × 10^−4^ (.05/62). ACS indicates acute chest syndrome; CKD, chronic kidney disease; eGFR, estimated glomerular filtration rate; HbF, fetal hemoglobin; MCH, mean corpuscular hemoglobin; MCV, mean corpuscular volume; mGFR, measured glomerular filtration rate; RBC, red blood cell; SCD, sickle cell disease; SpO_2_, hemoglobin oxygen saturation; TCD, transcranial Doppler; UACR, urine albumin-to-creatinine ratio; and VOC, vaso-occlusive crisis.

Concurrent α-thalassemia was consistently associated with increased hemoglobin levels (eg, continuous β, 0.39; 95% CI, 0.24-0.53) and reduced risk for elevated markers of hemolysis (eg, hemolytic component β, −0.70; 95% CI, −1.26 to −0.14), hepatomegaly (≤4 cm vs >4 cm β, 1.82; 95% CI, 0.51-3.31), biliary dysfunction (eg, bilirubin levels as high vs low β, −1.32; 95% CI, −2.51 to −0.13), stroke (eg, occurrence β, −0.85; 95% CI, −1.18 to −0.52), and kidney dysfunction (eg, albuminuria occurrence β, −1.10; 95% CI, −1.74 to −0.47) (Figure 2). While less clear, there may be increased risk of acute pain crisis (eg, vaso-occlusive crisis events per year β, 0.29; 95% CI, −0.16 to 0.74), acute splenic sequestration (β, 0.72; 95% CI, −0.19 to 1.62), and osteonecrosis (β, 1.06; 95% CI, −0.22 to 2.36) and reduced risk of leg ulcers (β, −0.30; 95% CI, −0.59 to −0.01) and priapism (β, −0.42; 95% CI, −0.75 to −0.08).

### Pathway Analysis

**Figure 3.**
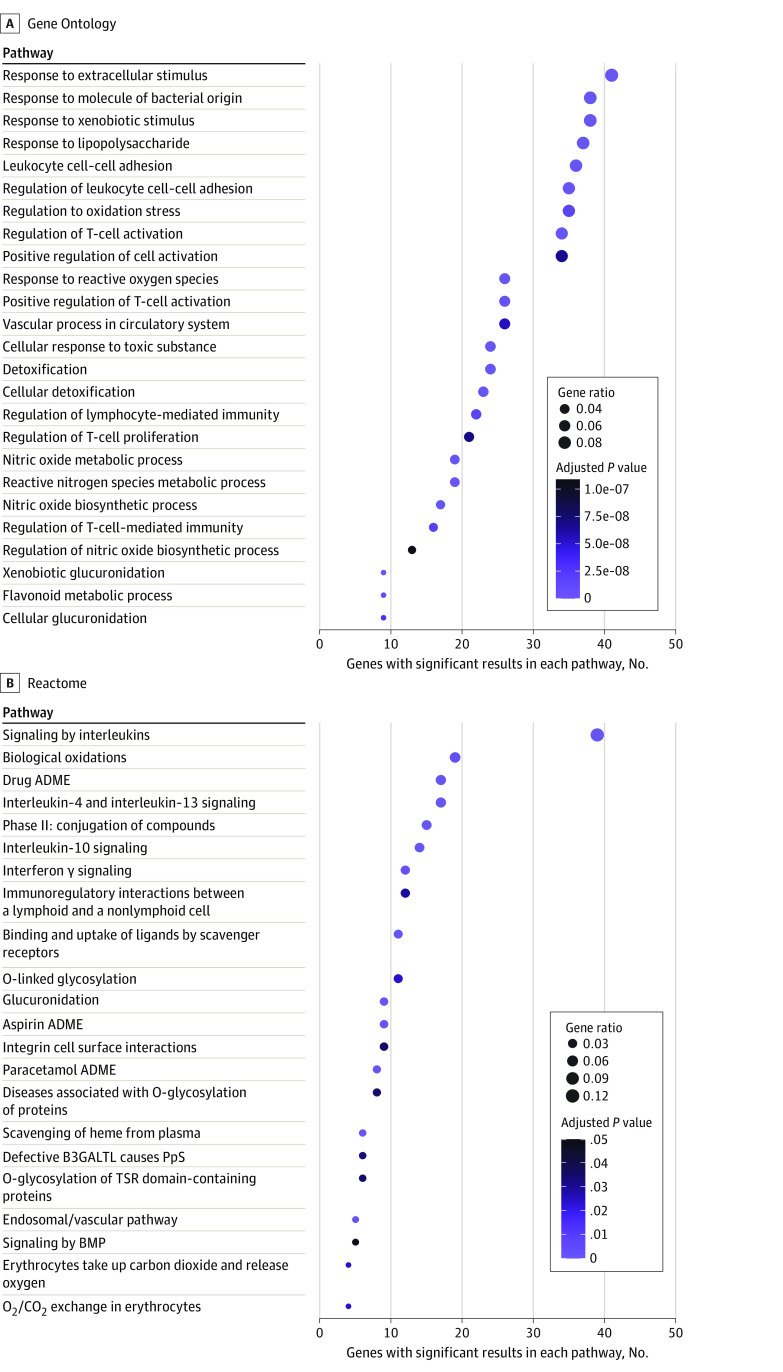
Enriched Pathways for Genes Reported to be Significantly Associated With Any Sickle Cell Disease–Related Outcome Gene Ontology– and Reactome-curated pathways were examined for any pathways with significant enrichment among all genes with at least 1 reported significant result. The top 25 and all 22 with adjusted *P* < .05 are reported for Gene Ontology and Reactome, respectively. Circle size represents the number of genes in that pathway out of the total submitted genes (gene ratio); circle color, degree of significance after adjusting for multiple testing (adjusted P value, using the Benjamini-Hochberg method). ADME indicates absorption, distribution, metabolism, and excretion; BMP, bone morphogenetic proteins; CO_2_, carbon dioxide; O_2_, oxygen; PpS, Peters-plus syndrome; and TSR, thrombospondin type 1 repeat.

Consistent with the known pathophysiology of SCD, genes with at least 1 significant result for any outcome were enriched for cellular adhesion, oxidative and toxic stress, inflammation, and blood vessel regulation, with 23 of the 25 most enriched GO pathways representing these processes (Figure 3; eTables 7 and 8 in Supplement 2). Phenotype category-specific pathway analyses (eTables 7 and 8 in Supplement 2) identified numerous other pathway enrichments, including the flavonoid metabolic GO pathway, for which genes associated with hyperbilirubinemia or biliary dysfunction, hemolysis, and anemia were enriched.

## Discussion

This comprehensive systematic review and meta-analysis consolidates current knowledge of SCD genetic modifiers, incorporating data from 571 publications from 1981 to 2023 and describing at least 29 670 unique individuals residing in 43 countries. These 571 studies were assessed for quality of study design and reporting, according to STREGA guidelines. Remarkably, only 1% of results reported across the last 43 years of work met minimum standards for cross-study meta-analysis due to variability in study designs, reporting practices, and phenotype or genotype definitions.

The differing methodologies and reporting practices between screened studies limited our ability to include, aggregate, or analyze published data. Many manuscripts used individuals without SCD as controls and were excluded, as this does not assess the genetic modifiers of SCD severity. Similarly, studies using biochemical measures as surrogates for genotypes (ie, glucose-6-phosphate dehydrogenase levels) were excluded because biochemical measurements do not always align with genotype. Other limitations included use of rudimentary statistical tests, rather than regression-based techniques adjusting for confounders, and inconsistent phenotype or genotype definitions. Despite these methodological challenges, we resolved some previous discrepant results and provide guidelines for future studies.

Across 571 manuscripts, we, as in a recent review,^585^ identified several genes associated with SCD complications in at least 2 studies. However, among those validated in meta-analysis, most were related to HbF levels. Specifically, meta-analyses confirmed the polygenic regulation of HbF: 10 SNVs in *BCL11A*, 8 in *HBS1L-MYB*, and 1 *HBG2* were significantly associated with HbF. Remarkably, 137 additional genes were reportedly associated with HbF but have not been confirmed due to lack of validation in a separate cohort or insufficient harmonization of phenotype (ie, dichotomous vs continuous, F cell percentage vs HbF percentage, unclear units) or genotype. Only 20%-50% of the genetic variability in HbF can be explained by currently validated variants (extended β-like globin locus, *BCL11A*, and *HBS1L*-*MYB*),^10,11,12,13^ which have relatively large association sizes. Most likely, many other loci with small association sizes or low frequency account for the remaining heritability. These additional modifier variants may be represented among the genes that lack validation.

This review also clarifies the role of α-thalassemia as a modifier of SCD severity by demonstrating that α-thalassemia was associated with reduced risk of clinically relevant SCD symptoms thought to be driven by hemolysis, including severe anemia, hyperbilirubinemia or gallstones, kidney dysfunction, and stroke. While this was a commonly accepted belief,^232^ our analysis consolidated the data to identify consistent trends among nonharmonized phenotypes with conflicting results.

To date, most validated studies of SCD modifiers have identified common variants with large association sizes (ie, “low-hanging fruit”) in relatively small cohorts. However, most genetic variation in health-related traits is driven by the interplay of many variants with small association sizes or low allele frequencies.^586^ Discovering such modifiers of SCD will require well-designed studies in larger cohorts using modern approaches to genetic analyses, including genome-wide association studies, adjustment for covariates, and, as discussed by Pincez, et al,^585^ multiomics. Future studies following best practices may also confirm candidate associations that have been reported in only 1 study. Moreover, studies in African cohorts could identify heretofore undiscovered variants with different frequencies in European and admixed cohorts. Most high-income countries, such as the US, have relatively few patients with SCD available for genetic studies. By contrast, while only 22% of individuals studied to date were from African cohorts, millions of individuals with SCD reside in sub-Saharan Africa, reflecting a fertile region for future research.

Advanced statistical and machine learning approaches, will also prove beneficial. For example, polygenic scores combining variants of small association sizes have been generated, including for HbF,^17,81,104,122,544^ pain,^17,81,104^ kidney,^20,170^ and cerebrovascular outcomes^34,36,132^ in SCD. However, those scores generally account for a small fraction of heritability and thus lack clinical utility. Improving polygenic scores for SCD phenotypes will require identifying more variants, validation, and rigorous testing, all of which would benefit from larger, more diverse cohorts and could be informed by analogous studies in non-SCD cohorts. Similarly, mendelian randomization, a method to explore causal relationships, has been used infrequently in SCD cohorts.^544,576^ In addition to increasing studies in Africa of both individuals with or without SCD, local ancestry inference in admixed individuals may help deconstruct associations driven by African ancestry.

Pathway analysis is another avenue for identifying potential candidate genes and generating clinically relevant hypotheses, even when traditional meta-analysis is not possible, as it can identify biologically meaningful pathways based on genes with significant associations and indicate other genes in these pathways that may contribute to disease risk. Among genes significantly associated with any SCD outcome in at least 1 study, we found enrichment in pathways controlling cellular adhesion, inflammation, response to toxic and oxidative stress, and blood vessel regulation, aligning with known disease pathophysiology. Other genes in those pathways are potential candidates for future investigation. We also identified potential therapeutic targets, such as flavonoid metabolic processes (of interest generally^587,588^ and in SCD^589,590^), which were enriched for genes associated with hemolysis, anemia, and hyperbilirubinemia or biliary complications.

### Limitations

This study has limitations that may confound or reduce the generalizability of our results. Subtype of SCD, ancestry, and hydroxyurea treatment status were not often reported in detail or, in the case of SCD subtype and ancestry, determined genetically; thus, we made no attempt to assess the difference between SCD subtypes or ancestries or to examine treatment response. Because most studies used a candidate gene approach, our results may be biased toward genes or pathways that were historically of high interest. Similarly, our analysis could be affected by unreported negative or contradictory results arising from positive publication bias. There are some methods, such as bayesian approaches, that cannot be integrated into a meta-analysis, resulting in some high-quality results being classified as exploratory. Finally, while our analysis categories allowed for a measure of study design and reporting rigor, they did not constitute a formal risk of bias assessment.

## Conclusions

Although this systematic review and meta-analysis assessed 571 manuscripts that collectively reported 17 757 genetic associations with outcomes related to SCD severity, those associations validated in cross-study meta-analysis were largely related to HbF. To accelerate the understanding of the genetic etiology of SCD, future genetic association studies should report sufficient information for results to be included in meta-analyses. Elements of contemporary study design and international collaborations will improve scientific rigor, reduce the risk of false positives, and expand generalizability of study results. To facilitate cross-study analysis, the use of consensus measures is recommended for phenotypes and exposures.^591,592^ Combined, these steps will generate the high quality results necessary to develop clinically actionable genetic tools.

## Notes

Supplement 1.
eMethods.
**eFigure 1.** PRISMA Flow Diagram For Manuscript Identification, Screening, and Inclusion **eFigure 2.** Map of Included Patient Cohort Locations by Number of Individuals **eFigure 3.** Data Strength Categories by Time **eFigure 4.** Number of Papers Reporting Significant Associations With Fetal Hemoglobin (HbF) and Number of Unique Variants for Genes With Significant Results Reported in at Least Three Manuscripts **eTable 4.** Phenotype Categories With Major Constituent Phenotypes **eTable 5.** Number of Unique Patients and Studies by Country
eReferences.

Supplement 2. **eTable 1.** Manuscripts Included in This Systematic Review and Meta-Analysis **eTable 2.** Data Extracted and Utilized in This Systematic Review and Meta-Analysis, Excluding Genome-Wide Summary Statistics **eTable 3.** Genome-Wide Summary Statistics Extracted and Utilized in This Systematic Review and Meta-Analysis **eTable 6.** Full List of Gene-Phenotype Category Associations **eTable 7.** Gene Ontology Pathway Analysis Results **eTable 8.** Reactome Pathway Analysis Results

Supplement 3.
Data Sharing Statement
