# Supplementary material for: Genetic Variation and Sickle Cell Disease Severity: A Systematic Review and Meta-Analysis
Source: JAMA Netw Open. 2023 Oct 18;6(10):e2337484. doi: 10.1001/jamanetworkopen.2023.37484 (PMC10585422; doi:10.1001/jamanetworkopen.2023.37484)
Supplement: Supplement 1. — eMethods. eFigure 1. PRISMA Flow Diagram For Manuscript Identification, Screening, and Inclusion eFigure 2. Map of Included Patient Cohort Locations by Number of Individuals eFigure 3. Data Strength Categories by Time eFigure 4. Number of Papers Reporting Significant Associations With Fetal Hemoglobin (HbF) and Number of Unique Variants for Genes With Significant Results Reported in at Least Three Manuscripts eTable 4. Phenotype Categories With Major Constituent Phenotypes eTable 5. Number of Unique Patients and Studies by Country eReferences. [file jamanetwopen-e2337484-s001.pdf]

Supplementary Online Content

Kirkham JK, Estepp JH, Weiss MJ, and Rashkin SR. Genetic variation and sickle cell disease severity: a systematic review and meta-analysis. *JAMA Netw Open*. 2023;5(10):e2337484. doi:10.1001/jamanetworkopen.2023.37484

eMethods.
eFigure 1. PRISMA Flow Diagram For Manuscript Identification, Screening, and Inclusion
eFigure 2. Map of Included Patient Cohort Locations by Number of Individuals
eFigure 3. Data Strength Categories by Time
eFigure 4. Number of Papers Reporting Significant Associations With Fetal Hemoglobin (HbF) and Number of Unique Variants for Genes With Significant Results Reported in at Least Three Manuscripts
eTable 4. Phenotype Categories With Major Constituent Phenotypes
eTable 5. Number of Unique Patients and Studies by Country
eReferences.

This supplementary material has been provided by the authors to give readers additional information about their work.

© 2023 Kirkham JK et al. *JAMA Network Open*.

eMethods

Search Terms

The following terms were used to search each database, respectively. The search was performed on May 16, 2023, and all manuscripts published prior to that date were considered.

PubMed: (sickle OR "sickle cell disease" OR “sickle cell anemia” OR “Hemoglobin SS” OR “Hemoglobin SC” OR “Hemoglobin SD” OR “sickle hemoglobin” OR "Anemia, Sickle Cell"[Mesh]) AND ("Genome-Wide Association Study"[Mesh] OR “GWA” OR “GWAS” OR "Genetic Association Studies"[Mesh] OR "Genetic Predisposition to Disease"[Mesh] OR "Polymorphism, Single Nucleotide"[Mesh] OR "Polymorphism, Genetic"[Mesh] OR "single nucleotide polymorphism*" OR "SNP" OR “SNPs” OR "Genetic Variation"[Mesh] OR "genetic variant*" OR "gene variant*" OR "Genes, Modifier"[Mesh] OR "gene modifier*" OR "genetic modifier*" OR "Haplotypes"[Mesh] OR "haplotype*" OR "candidate gene*" OR "candidate variant*" OR "Quantitative Trait Loci"[Mesh] OR “eQTL” OR “QTL” OR "Genetic Loci"[Mesh] OR "locus" OR "loci" OR “alpha thalassemia”)

Web of Science: ALL=((sickle OR "sickle cell disease" OR “sickle cell anemia” OR “Hemoglobin SS” OR “Hemoglobin SC” OR “Hemoglobin SD” OR “sickle hemoglobin”) AND ("Genome-Wide Association Stud*" OR “GWA” OR “GWAS” OR "Genetic Association Stud*" OR "Genetic Predisposition to Disease" OR "genetic polymorphism*" OR "single nucleotide polymorphism*" OR "SNP" OR “SNPs” OR "Genetic Variation" OR "genetic variant*" OR "gene variant*" OR "gene modifier*" OR "genetic modifier*" OR "haplotype*" OR "candidate gene*" OR "candidate variant*" OR "Quantitative Trait Loc*" OR “eQTL” OR “QTL” OR "Genetic Loc*" OR "locus" OR "loci" OR “alpha thalassemia”))

Scopus: TITLE-ABS-KEY((sickle OR "sickle cell disease" OR “sickle cell anemia” OR “Hemoglobin SS” OR “Hemoglobin SC” OR “Hemoglobin SD” OR “sickle hemoglobin”) AND ("Genome-Wide Association Stud*" OR “GWA” OR “GWAS” OR "Genetic Association Stud*" OR "Genetic Predisposition to Disease" OR "genetic polymorphism*" OR "single nucleotide polymorphism*" OR "SNP" OR “SNPs” OR "Genetic Variation" OR "genetic variant*" OR "gene variant*" OR "gene modifier*" OR "genetic modifier*" OR "haplotype*" OR "candidate gene*" OR "candidate variant*" OR "Quantitative Trait Loc*" OR “eQTL” OR “QTL” OR "Genetic Loc*" OR "locus" OR "loci" OR “alpha thalassemia”))

Data Extraction

Data from the 571 included manuscripts[1-571] were extracted by one of two investigators (JKK and SRR), with discussions to resolve any uncertainty. Information was extracted regarding study design, phenotype definitions, genotype ascertainment methods and quality control performed, statistical analysis, and all genetic association results from the main text and available supplemental materials (**eTable 2**). Each extracted result was determined to be significant based on the reporting manuscript’s criteria as these definitions have shaped the current knowledge base, even though results reported without any multiple testing correction may be more likely to be false positive associations, incorrectly indicating an association is significant. For papers with undefined significance thresholds, if the analysis was genome-wide, a significance threshold of $5x10^{-8}$ was used, and 0.05 was used otherwise.

One manuscript included genome-wide summary statistics for all 237,078 variants tested, and another included the top 4,000 results for each of three different analyses (12,000 total). While we extracted these results (**eTable 3**) and included them in the meta-analyses, we excluded them from tabulation of results to avoid over-inflation of counts.

Study Design Evaluation

Study design was determined by the original author’s designation, when available. As most publications did not clearly self-identify an approach, the primary method of data collection and analysis was determined as follows: For studies in which data collection was done at a single point of time, with respect to each individual subject, and there was not a longitudinal approach to data analysis, the study was categorized as cross-sectional. Studies which selected controls using a pre-determined ratio in respect to the cases of interest were categorized as case-control. All case-control studies, save one, were retrospective in nature. Investigations of a cohort involving collection of data over numerous time points, in relation to each individual subject, were categorized as either prospective or retrospective cohort studies, based on whether the exposure of interest (i.e., genetic mutation) was identified and subjects were consented prior to or after the period of data collection. Finally, studies in which an intervention was selectively applied between

© 2023 Kirkham JK et al. *JAMA Network Open*.

a control group and an experimental group, even if the intervention was not thought to have a direct connection to the exposure (i.e., genetic mutation) data being extracted, were categorized as clinical trials. If a single report made use of multiple cohorts and each cohort was investigated using a different study design, then multiple study designs were listed.

Phenotype Categorization

We classified each result into one of 25 general categories based on reported phenotype (**eTable 4**), with the aim of consolidating diverse phenotypes into common disease manifestations or organ-system categories. These categories were then grouped into broad sets based on the type of complication or measure: 1) acute SCD-related complications, 2) chronic SCD-related complications, 3) hematological parameters and biomarkers or disease severity, and 4) general or mixed measurements of SCD severity.

Prevalence estimates for each phenotype category were determined from the most recent published measurements within the adult SCD population within the United States, regardless of SCD subtype, when available.[310,430,441,449,572-583] When United States prevalence measurements were not available, data from countries with the most similar healthcare infrastructure and life expectancy were utilized. Specifically, estimates for "Infection (excludes respiratory infections)", "Priapism", "Acute splenic sequestration", and "Kidney dysfunction" were from the United Kingdom, Nigeria, France, and France, respectively. Additionally, only pediatric estimates were available for "Acute splenic sequestration", "Hyperbilirubinemia, cholelithiasis, cholecystitis, or cholecystectomy", "Splenic dysfunction", and "Anemia".

Gene Annotation Harmonization

Gene annotations were standardized across all extraction results, using reported annotations, dbSNP, and NCBI Gene. Intergenic single nucleotide polymorphisms (SNPs) were annotated to the nearest up- and downstream genes. Gene-dense regions were clustered due to multiple possible SNP-gene annotations, including the extended beta-globin locus, the olfactory receptor gene cluster, the HLA region, and the HPA gene family. For gene labels not found in NCBI Gene's list of canonical gene identifiers and synonyms (downloaded June 27, 2023), we used dbSNP and the reporting paper's annotations to ensure all unique genes with significant associations were captured. We then obtained Entrez IDs for each gene, which were used as input for the pathway analyses.

Unique Sample Size Tabulation

An estimate of the total number of unique individuals studied, including the number of pediatric patients, was determined for the studies reviewed, accounting for repeated evaluation of the same cohort. Extracted sample sizes were totaled, assuming the most overlap possible where not specifically excluded by the original authors' cohort description or determined to be temporally or geographically distinct, based on subject age, publication date, and available information on location. This summation represents the fewest unique individuals that would satisfy the cohort sizes outlined in all reviewed publications, though it likely underestimates the total subject count in an effort to avoid counting individuals more than once. There was no attempt to account for the potential of migration, so two geographically distant recruiting centers were assumed to represent non-overlapping cohorts. If no cohort location was reported in the manuscript, the corresponding author's institution was used. As 17 manuscripts include a mixed United States and Canada cohort, the total patient count from these two countries were combined. Cohorts located in any region of the French Republic, including the overseas departments of French Guiana, Guadeloupe, Martinique, and Mayotte, were grouped with metropolitan France under the simplified heading of France. The number of studies reporting on individuals from each country was also tabulated. As 55 studies reported on cohorts from at least two different countries, the total number of times all cohorts have been studied was greater than the total number of studies reviewed.

Data Strength Category Definitions

We used the STrengthening the REporting of Genetic Association Studies (STREGA) guidelines[584] – an extension of the Strengthening the Reporting of Observational Studies in Epidemiology (STROBE) statement[585] – to create three data strength categories into which all results were classified, defined as:

1. <u>Exploratory</u> – results which conduct genotype-phenotype association with a formal, statistical test but lack some information required for inclusion in a meta-analysis.
2. <u>Meta-Suitable</u> – results which contain at least the minimum required information for inclusion in a meta-analysis statistically: a) clearly defined phenotypic outcome, including comparison groups, association

direction, units, and any data transformations performed (STREGA 7a, 8a); b) clearly defined genetic variables, including genetic model (i.e., additive, dominant), with all genotypes defined by allele and association direction (STREGA 7b); c) exact statistical test conducted (STREGA 12); d) number of individuals used in analysis (STREGA 13); e) association size (i.e., beta, odds ratio, hazard ratio, etc.) and direction and a measure of variability (i.e., standard error [SE], confidence interval [CI], P-value) (STREGA 16a).

3. Contemporary – results which contain all criteria required for meta-suitable as well as further elements crucial for genetic association studies: a) genotyping/sequencing methods and quality control steps, including consideration of Hardy-Weinberg Equilibrium (STREGA 8a, 12f, 12g); b) accounting for population stratification (STREGA 12h); c) multiple testing correction (STREGA 12i, 16d); d) assessment of and, where relevant, accounting for related samples (STREGA 12j); e) evaluation of and adjustment for potential confounders (STREGA 12a, 16a). While not explicitly listed in STREGA, due to the potential for false positives, we have also included external validation as a required element for this category.

Meta-analysis

Due to the high level of variability in study design, data transformation, and covariate adjustment among results included in the meta-analyses, in lieu of either a fixed- or random-effects meta-analysis, we used a weighted Z-score-based approach implemented by METAL,[586] as we indicated in an amendment to our PROSPERO registration. This method is robust to differences in ethnicity, phenotype distribution, and other factors, as association sizes are not directly combined. Instead, for each study, Z-scores are calculated, summarizing the magnitude and association direction using P-values. An overall Z-score and P-value are calculated from a weighted sum of the individual scores, where weights are proportional to the study's sample size. As this method requires P-values, for results with only SE and/or CI, we calculated P-values using the provided information and an appropriate formula. If only CI was provided, when the two SE estimates differed by more than 20% of the larger value, likely due to rounding in the published results, that result was excluded; otherwise, the two estimates were averaged. Meta-analysis results were determined to be significant if the P-value satisfied the Bonferroni-corrected threshold (0.05/62). As typical metrics of heterogeneity used for meta-analyses, such as Cochran's Q and I^2, rely on the pooled association size, we evaluated component studies for agreement with the meta-analysis result in terms of significance and directionality.

α-Thalassemia

As the relationship of concomitant α-thalassemia to SCD-related outcomes has been investigated for years, we compared all published results to summarize what is known. We restricted this assessment to extracted results classified as meta-suitable or contemporary. Additionally, we excluded results modeling one and two deletions separately; we further restricted to dominant genetic models unless the only evaluations performed for an outcome were additive per each allele copy.

For comparison, non-continuous trait measures of association (i.e., odds ratio, hazard ratio) were log-transformed to convert to the beta scale (i.e., log odds ratio, log hazard ratio). For phenotypes with results from multiple cohorts, we first conducted fixed-effects meta-analyses to obtain an overall association size and CI, as our primary meta-analysis results did not provide global association sizes and CIs, and the fixed-effects P-values more closely matched our METAL P-values than the random-effects P-values. A forest plot was generated to evaluate the relationship between the presence of α-thalassemia deletions compared to no deletions on all reported outcomes.

Pathway Enrichment Analysis

From the list of extracted results, we compiled a list of unique genes to which significant results were annotated. We used the R packages "clusterProfiler"[587] and "ReactomePA"[588] to conduct over-representation analyses using the Gene Ontology (GO)[589] and Reactome[590] databases. Briefly, a list of input genes was compared to curated pathway databases, testing whether there are any pathways containing more genes from the input list than expected by chance, correcting for multiple testing. We first conducted this assessment across all reported significant results, regardless of phenotype. We then repeated the analysis for each phenotype category with significant results in at least 35 genes. Each P-value was corrected internally for multiple testing using the Benjamini-Hochberg method[591], and pathways were considered significant and reported if both P and adjusted P <0.05.

© 2023 Kirkham JK et al. *JAMA Network Open*.

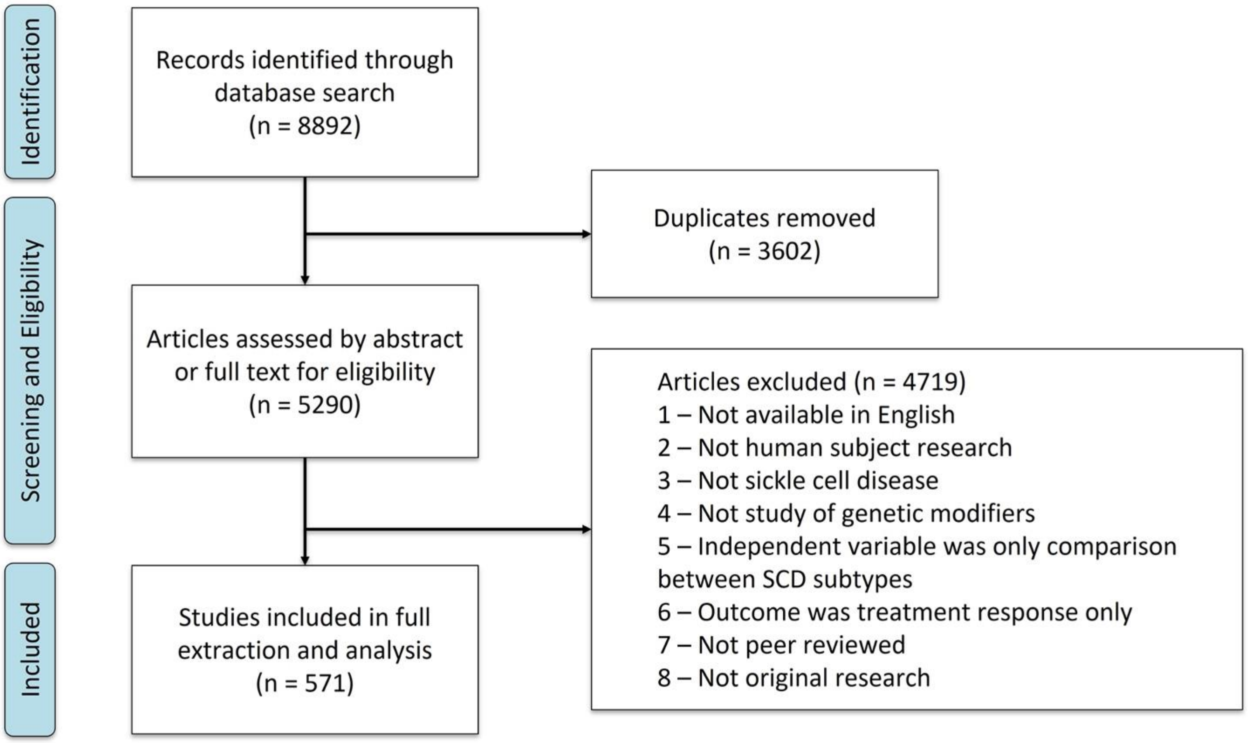

eFigure 1. **PRISMA Flow Diagram For Manuscript Identification, Screening, and Inclusion.** See eMethods for full search terms for each database.

© 2023 Kirkham JK et al. *JAMA Network Open*.

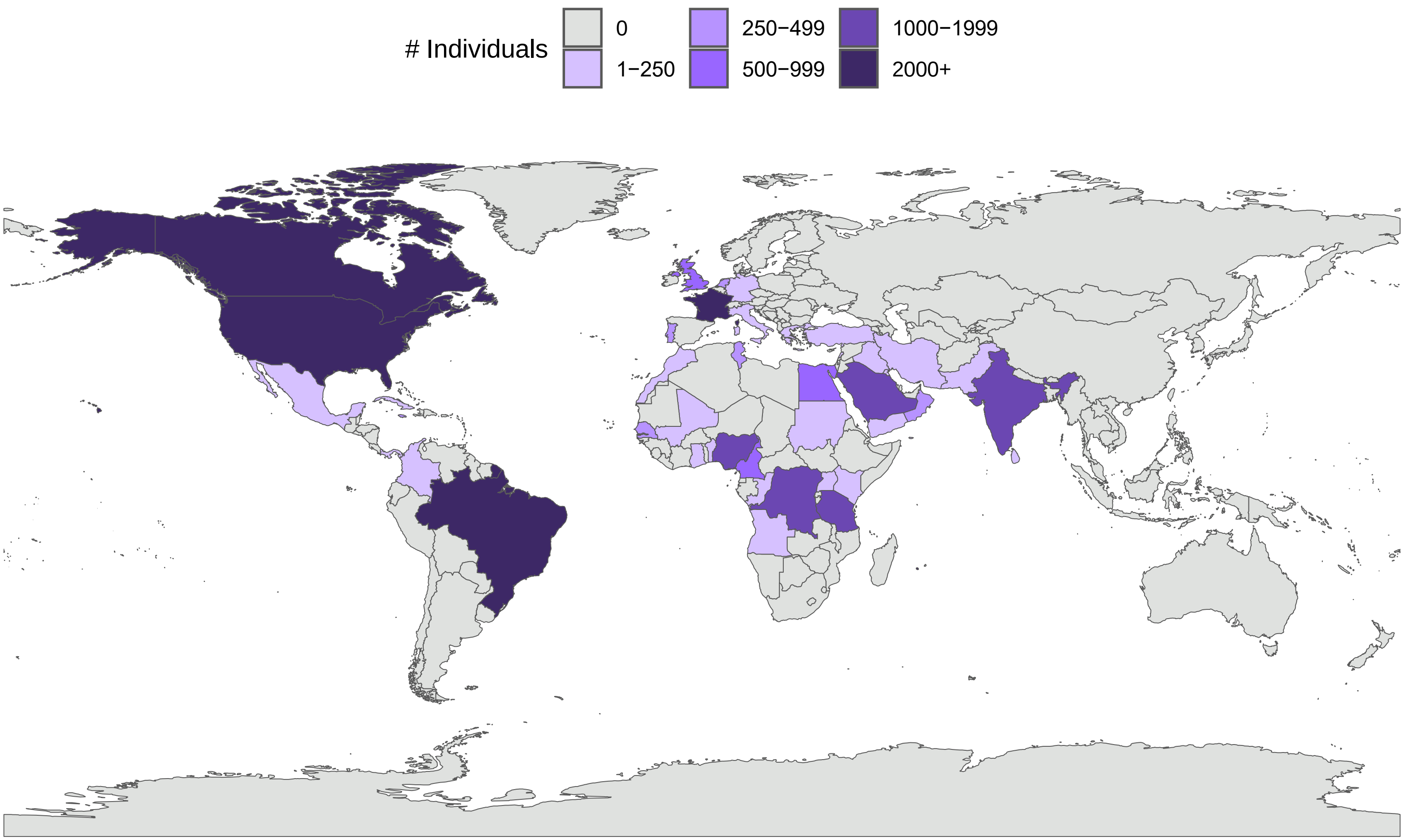

eFigure 2. Map of Included Patient Cohort Locations by Number of Individuals. Countries in which sickle cell cohorts were studied in at least one manuscript included in this review are colored in purple, shaded by number of individuals. If the origin of the cohort was not reported in the manuscript, the corresponding author's institution is shown. Cohorts located in any region of the French Republic, including the overseas departments of French Guiana, Guadeloupe, Martinique, and Mayotte, were grouped with metropolitan France under the simplified heading of France and shaded if individuals were included from any. As many cohorts in the United States and Canada recruited patients from the other, the total patient count from these two countries were grouped.

© 2023 Kirkham JK et al. *JAMA Network Open*.

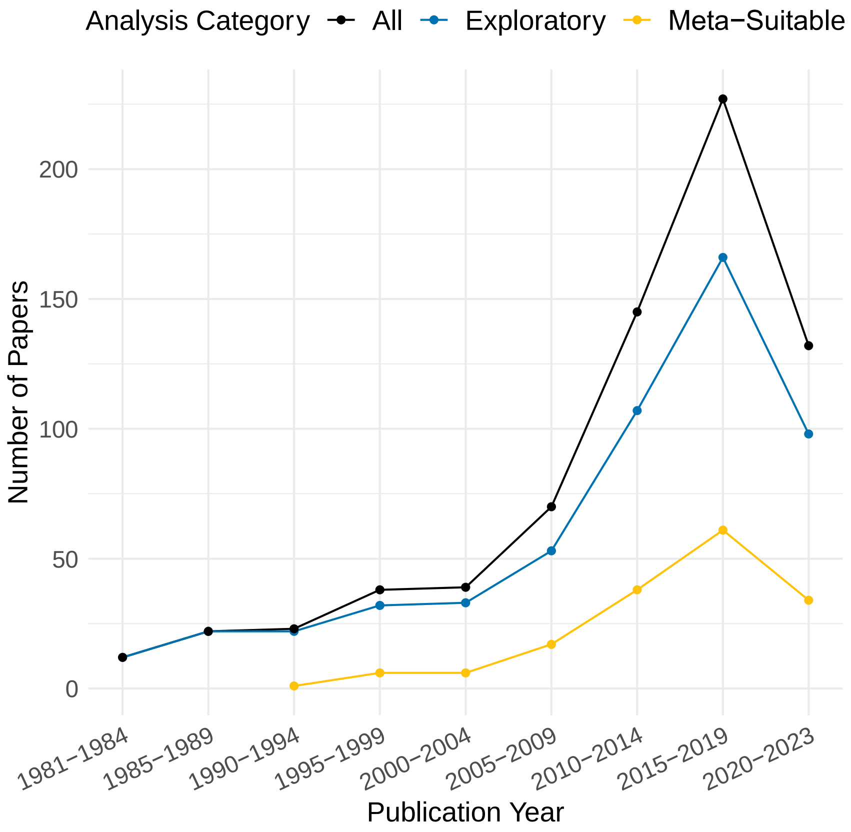

eFigure 3. Data Strength Categories by Time. The total number of papers published within each year range are plotted in black. Papers publishing exploratory or meta-suitable/contemporary results are within each year range are plotted in blue and yellow, respectively. Papers reporting results for both categories were counted in each.

© 2023 Kirkham JK et al. *JAMA Network Open*.

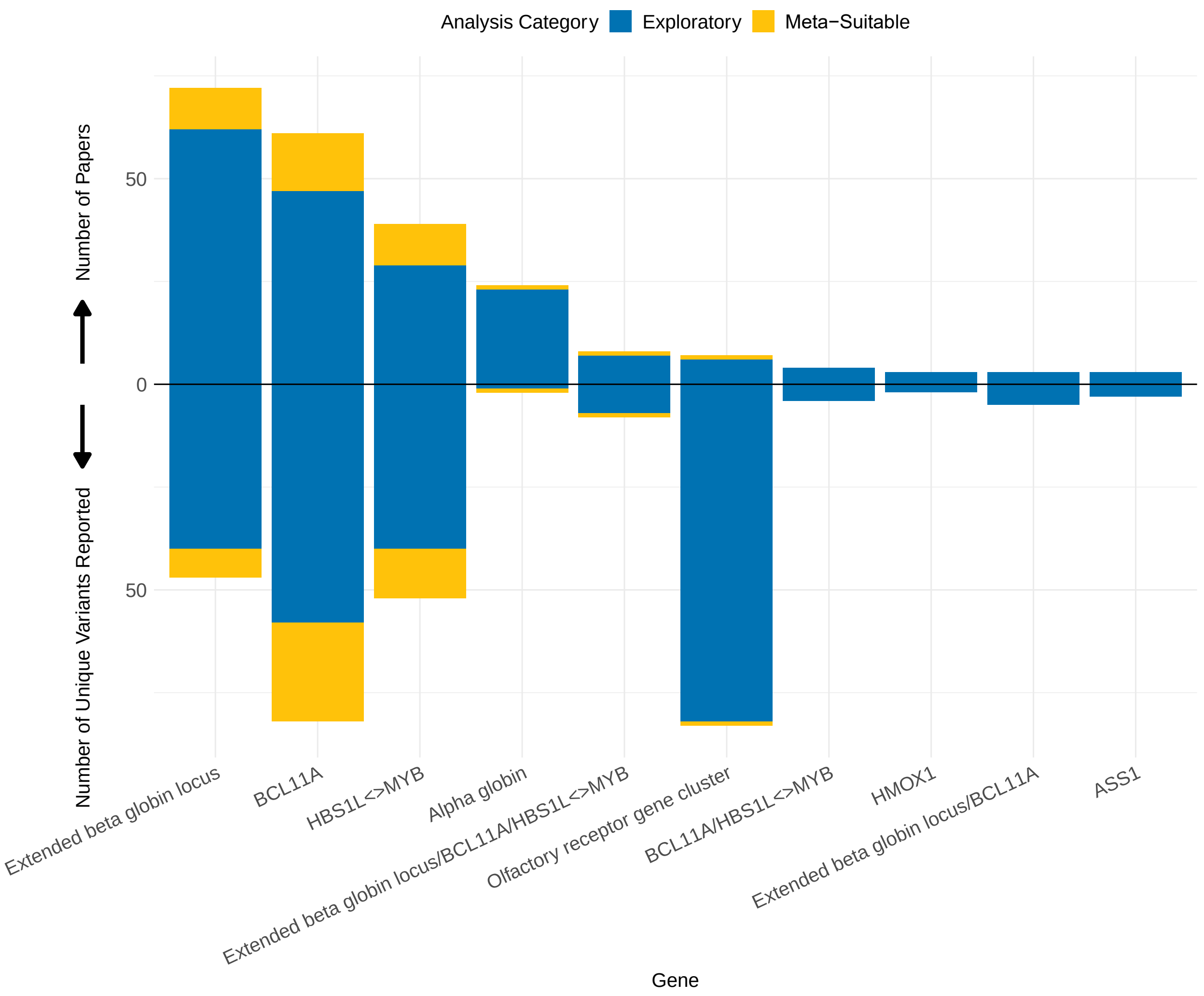

eFigure 4. Number of Papers Reporting Significant Associations With Fetal Hemoglobin (HbF) and Number of Unique Variants for Genes With Significant Results Reported in at Least Three Manuscripts. Papers reporting exploratory results are tabulated in blue, and those reporting meta-suitable results are tabulated in yellow. Similarly, results classified as exploratory and meta-suitable were tabulated in blue and yellow, respectively. Some papers report results of both categories and were counted in both, and some variants were classified as both categories and were counted in both. Gene names separated by arrows (“<>”) indicate SNPs in the intergenic region were associated with HbF, and gene names separated by a slash (“/”) indicate that both genes were implicated, as in a polygenic score.

© 2023 Kirkham JK et al. *JAMA Network Open*.

eTable 4. Phenotype Categories With Major Constituent Phenotypes. Specific phenotypes within each category are shown if they were reported at least twice and not described in the category name. ACS, acute chest syndrome; HbF, fetal hemoglobin; CNS, central nervous system; TCD, transcranial doppler; MRI, magnetic resonance imaging; BMI, body mass index.

Phenotype Category	Specific phenotypes
**Acute SCD-Related Complications**	
Acute pain episode	Analgesic dosing, dactylitis, hand and foot syndrome, health care utilization, veno-occlusive crisis, other acute pain
ACS, pneumonia, respiratory infection	
Infection (excludes respiratory infections)	Bacteremia, bacterial infection, hepatitis C, malaria, meningitis, osteomyelitis, sepsis, urinary tract infection
Priapism	
Acute splenic sequestration	
Other acute phenotype	Age at presentation for acute complication, aplastic crisis, hospitalization for multiple or unspecified acute complications, thrombosis
**Chronic SCD-Related Complications**	
Allo- or autoantibody or transfusion reaction	
Cerebrovascular disease	Cerebrovascular accident (overt or silent), CNS bloodflow, CNS vasculopathy, TCD abnormality
Cardiopulmonary dysfunction	Cardiopulmonary complications, echocardiogram or cardiac MRI measurements, exercise tolerance tests, oxygen saturation, pulmonary hypertension
Kidney dysfunction	Blood urea nitrogen (BUN), chronic kidney disease (CKD), creatinine, cystatin C, end stage renal disease (ESRD), glomerular filtration rate (GFR), hyperfiltration, renal imaging measurements, urine studies
Hyperbilirubinemia, cholelithiasis, cholecystitis, or cholecystectomy	
Osteonecrosis	
Chronic pain	Composite pain index
Leg ulcers	
Iron overload	Iron chelation therapy, iron studies, liver iron content
Retinopathy	
Splenic dysfunction	Impaired splenic function, splenectomy, splenomegaly
Other chronic phenotype	BMI, bone age, diabetes, fertility and pregnancy outcome, height, hepatomegaly, osteoporosis, other chronic disease, vascular complication, vascular reactivity, weight
**Hematologic Parameters and Biomarkers of Disease Severity**	
HbF	F cells, gamma globin
Other hematologic parameter	Blood viscosity, complete blood counts, hemoglobin identification, hemorheology, leukocyte subsets, other red blood cell studies
Hemolysis	Aspartate aminotransferase (AST), lactate dehydrogenase (LDH), hemoglobinuria, hemolysis-associated complications, hemolysis score/index, hyperhemolysis, jaundice, red blood cell survival, reticulocyte count
Anemia	Anemia, erythropoietin level, hemoglobin, or transfusion history
Oxidative stress	Advanced oxidation protein products (AOPP), catalase (CAT), ferric reducing antioxidant power (FRAP), glutathione, malondialdehyde (MDA), myeloperoxidase (MPO), nitrate, nitrite, superoxide dismutase (SOD), thiobarbituric acid-reacting substances (TBARS), thiol, trolox equivalent antioxidant capacity (TEAC)
Other parameter or biomarker	Gene expression measurements, heart rate, serum electrolyte and protein concentrations, systemic blood pressure
**General or Mixed Measurement of SCD Severity**	Combination of acute and chronic complications, death, disease severity index, organ failure, total days in hospital

© 2023 Kirkham JK et al. *JAMA Network Open*.

eTable 5. Number of Unique Patients and Studies by Country. Sample sizes were determined assuming the most overlap possible where not specifically excluded by the original authors' cohort description or otherwise determined to be distinct. This summation represents the fewest unique individuals that would satisfy the cohort sizes outlined in all reviewed publications (see **eMethods**). ND = Not determinable

Country	Total Unique Patients	Children	Studies Including Country's Patients
**TOTAL**	**29670**	**14970**	**571 (55 multinational)**
USA and Canada	9635	3728	166
Brazil	3397	1744	111
France	2445	1476	66
India	1631	1200	41
DRC	1356	361	5
Tanzania	1213	910	11
Nigeria	1202	539	13
Saudi Arabia	1194	330	28
United Kingdom	899	283	41
Cameroon	730	406	13
Bahrain	577	377	8
Egypt	508	358	24
Oman	495	495	6
Jamaica	407	356	26
Senegal	403	322	9
Tunisia	358	242	9
Netherlands	342	0	2
Portugal	296	252	7
Ghana	244	131	2
Kuwait	237	140	15
Angola	200	200	4
Sudan	166	33	1
Germany	164	131	1
Lebanon	163	25	2
Uganda	142	142	2
Benin	128	107	3
Iraq	128	64	1
Kenya	128	128	2
Congo	116	110	2
Yemen	102	102	2
Panama	100	100	2
Morocco	80	80	1
Greece	79	0	1
Cuba	65	ND	1
Colombia	60	ND	2
Italy	60	60	1
Sri Lanka	60	ND	1
Iran	52	14	2
Pakistan	41	ND	1
Turkey	31	0	1
Mexico	24	12	1
Mali	12	12	1

© 2023 Kirkham JK et al. *JAMA Network Open*.

eReferences

1. Rampersaud E, Kang G, Palmer LE, et al. A polygenic score for acute vaso-occlusive pain in pediatric sickle cell disease. *Blood Adv*. Jul 27 2021;5(14):2839-2851. doi:10.1182/bloodadvances.2021004634
2. Akbulut-Jeradi N, Fernandez MJ, Al Khaldi R, Sukumaran J, Adekile A. Unique Polymorphisms at BCL11A, HBS1L-MYB and HBB Loci Associated with HbF in Kuwaiti Patients with Sickle Cell Disease. *J Pers Med*. Jun 17 2021;11(6)doi:10.3390/jpm11060567
3. Adabale A, Makanjuola SBL, Akinbami A, et al. Frequency of beta S globin gene haplotypes among sickle cell patients in Nigeria. *J Int Med Res*. Jun 2021;49(6):3000605211019918. doi:10.1177/03000605211019918
4. Rashkin SR, Rampersaud E, Kang G, et al. Generalization of a genetic risk score for time to first albuminuria in children with sickle cell anaemia: SCCRIP cohort study results. *Br J Haematol*. Jul 2021;194(2):469-473. doi:10.1111/bjh.17647
5. Batista J, Pereira-Martins DA, Falcao DA, et al. Association of KLOTHO polymorphisms with clinical complications of sickle cell anemia. *Ann Hematol*. Aug 2021;100(8):1921-1927. doi:10.1007/s00277-021-04532-w
6. Delgadinho M, Ginete C, Santos B, Miranda A, Brito M. Genotypic Diversity among Angolan Children with Sickle Cell Anemia. *Int J Environ Res Public Health*. May 19 2021;18(10)doi:10.3390/ijerph18105417
7. Alnafie AN, Alateeq SA, Al-Muhanna FA, et al. Exome sequencing in high and low fetal haemoglobin Arab-Indian haplotype sickle cell disease. *Br J Haematol*. Jul 2021;194(2):e61-e64. doi:10.1111/bjh.17542
8. Page GP, Kanias T, Guo YJ, et al. Multiple-ancestry genome-wide association study identifies 27 loci associated with measures of hemolysis following blood storage. *J Clin Invest*. Jul 1 2021;131(13)doi:10.1172/JCI146077
9. Ojewunmi OO, Adeyemo TA, Oyetunji AI, Benn Y, Ekpo MG, Iwalokun BA. Association of alpha-thalassemia and Glucose-6-Phosphate Dehydrogenase deficiency with transcranial Doppler ultrasonography in Nigerian children with sickle cell anemia. *J Clin Lab Anal*. Jun 2021;35(6):e23802. doi:10.1002/jcla.23802
10. Brousse V, Pondarre C, Kossorotoff M, et al. Brain injury pathophysiology study by a multimodal approach in children with sickle cell anemia with no intra or extra cranial arteriopathy. *Haematologica*. Apr 22 2021;doi:10.3324/haematol.2020.278226
11. Ngo-Bitoungui VJ, Belinga S, Mnika K, et al. Investigations of Kidney Dysfunction-Related Gene Variants in Sickle Cell Disease Patients in Cameroon (Sub-Saharan Africa). *Front Genet*. 2021;12:595702. doi:10.3389/fgene.2021.595702
12. Hatzlhofer BLD, Pereira-Martins DA, de Farias Domingos I, et al. Alpha thalassemia, but not beta(S)-globin haplotypes, influence sickle cell anemia clinical outcome in a large, single-center Brazilian cohort. *Ann Hematol*. Apr 2021;100(4):921-931. doi:10.1007/s00277-021-04450-x
13. Romana M, Reminy K, Moeckesch B, et al. Loss of alpha globin genes is associated with improved microvascular function in patients with sickle cell anemia. *Am J Hematol*. May 1 2021;96(5):E165-E168. doi:10.1002/ajh.26126
14. Figueiredo CVB, Santiago RP, da Guarda CC, et al. Priapism in sickle cell disease: Associations between NOS3 and EDN1 genetic polymorphisms and laboratory biomarkers. *PLoS One*. 2021;16(2):e0246067. doi:10.1371/journal.pone.0246067
15. Batista J, Arcanjo GS, Batista THC, et al. Influence of UGT1A1 promoter polymorphism, alpha-thalassemia and beta(s) haplotype in bilirubin levels and cholelithiasis in a large sickle cell anemia cohort. *Ann Hematol*. Apr 2021;100(4):903-911. doi:10.1007/s00277-021-04422-1
16. Al-Ali AK, Alsulaiman A, Alfarhan M, et al. Sickle cell disease in the Eastern Province of Saudi Arabia: Clinical and laboratory features. *Am J Hematol*. Apr 1 2021;96(4):E117-E121. doi:10.1002/ajh.26096
17. Kumar R, Yadav R, Mishra S, et al. Kruppel-like factor 1 (KLF1) gene single nucleotide polymorphisms in sickle cell disease and its association with disease-related morbidities. *Ann Hematol*. Feb 2021;100(2):365-373. doi:10.1007/s00277-020-04381-z
18. Brewin JN, Rooks H, Gardner K, et al. Genome wide association study of silent cerebral infarction in sickle cell disease (HbSS and HbSC). *Haematologica*. Jun 1 2021;106(6):1770-1773. doi:10.3324/haematol.2020.265827

© 2023 Kirkham JK et al. *JAMA Network Open*.

19. Saraf SL, Zhang X, Shah BN, et al. Engulfment and cell motility 1 (ELMO1) and apolipoprotein A1 (APOA1) as candidate genes for sickle cell nephropathy. *Br J Haematol*. May 2021;193(3):628-632. doi:10.1111/bjh.17224
20. Joly P, Bonello-Palot N, Badens C, et al. HbF-promoting polymorphisms may specifically reduce the residual risk of cerebral vasculopathy in SCA children with alpha-thalassemia. *Clin Hemorheol Microcirc*. 2021;77(3):267-272. doi:10.3233/CH-200951
21. Santiago RP, Figueiredo CVB, Fiuza LM, et al. Transforming Growth Factor Beta Receptor 3 Haplotypes in Sickle Cell Disease Are Associated with Lipid Profile and Clinical Manifestations. *Mediators Inflamm*. 2020;2020:3185015. doi:10.1155/2020/3185015
22. Hariharan P, Chavan V, Nadkarni A. Significance of heme oxygenase-1(HMOX1) gene on fetal hemoglobin induction in sickle cell anemia patients. *Sci Rep*. Oct 28 2020;10(1):18506. doi:10.1038/s41598-020-75555-y
23. Urio F, Nkya S, Rooks H, et al. F cell numbers are associated with an X-linked genetic polymorphism and correlate with haematological parameters in patients with sickle cell disease. *Br J Haematol*. Dec 2020;191(5):888-896. doi:10.1111/bjh.17102
24. Santiago RP, Figueiredo CVB, Fiuza LM, et al. TGFBR3 Polymorphisms (rs1805110 and rs7526590) Are Associated with Laboratory Biomarkers and Clinical Manifestations in Sickle Cell Anemia. *Dis Markers*. 2020;2020:8867986. doi:10.1155/2020/8867986
25. Tozatto-Maio K, Girot R, Ly ID, et al. Polymorphisms in Inflammatory Genes Modulate Clinical Complications in Patients With Sickle Cell Disease. *Front Immunol*. 2020;11:2041. doi:10.3389/fimmu.2020.02041
26. Gueye Tall F, Martin C, Ndour EHM, et al. Influence of Oxidative Stress Biomarkers and Genetic Polymorphisms on the Clinical Severity of Hydroxyurea-Free Senegalese Children with Sickle Cell Anemia. *Antioxidants (Basel)*. Sep 14 2020;9(9)doi:10.3390/antiox9090863
27. Brewin JN, Smith AE, Cook R, et al. Genetic Analysis of Patients With Sickle Cell Anemia and Stroke Before 4 Years of Age Suggest an Important Role for Apoliprotein E. *Circ Genom Precis Med*. Oct 2020;13(5):531-540. doi:10.1161/CIRCGEN.120.003025
28. Sinha S, Jit BP, Patro ARK, et al. Influence of rs1042713 and rs1042714 polymorphisms of beta2-adrenergic receptor gene with erythrocyte cAMP in sickle cell disease patients from Odisha State, India. *Ann Hematol*. Dec 2020;99(12):2737-2745. doi:10.1007/s00277-020-04254-5
29. Elenga N, Cuadro-Alvarez E, Martin E, Njuieyon F, Defo A, Maniassom C. Influence of beta-cluster haplotypes, alpha-gene status and UGTA1 polymorphism on clinical and hematological data in sickle-cell disease children from French Guiana. *PLoS One*. 2020;15(9):e0238691. doi:10.1371/journal.pone.0238691
30. Khan J, Muhammad D, Ismail M, Khan I, Zia ur R, Niaz S. Consanguinity, the driving force behind inheritance of HbS-beta thalassemia in Southern Districts of KP. *J Pak Med Assoc*. Jun 2020;70(6):978-983. doi:10.5455/JPMA.300622
31. El-Ghamrawy M, Yassa ME, Tousson AMS, et al. Association between BCL11A, HSB1L-MYB, and XmnI gammaG-158 (C/T) gene polymorphism and hemoglobin F level in Egyptian sickle cell disease patients. *Ann Hematol*. Oct 2020;99(10):2279-2288. doi:10.1007/s00277-020-04187-z
32. Gordeuk VR, Shah BN, Zhang X, et al. The CYB5R3(c) (.350C>G) and G6PD A alleles modify severity of anemia in malaria and sickle cell disease. *Am J Hematol*. Nov 2020;95(11):1269-1279. doi:10.1002/ajh.25941
33. Costa Neto A, Santos F, Ribeiro I, et al. FcgammaR2B B2.4 haplotype predicts increased risk of red blood cell alloimmunization in sickle cell disease patients. *Transfusion*. Jul 2020;60(7):1573-1578. doi:10.1111/trf.15832
34. Pereira-Martins DA, Domingos IF, Belini-Junior E, et al. Association of HMIP1 C-893A polymorphism and disease severity in patients with sickle cell anemia. *Hematol Transfus Cell Ther*. Jun 4 2020;doi:10.1016/j.htct.2020.03.006
35. Santos B, Delgadinho M, Ferreira J, et al. Co-Inheritance of alpha-thalassemia and sickle cell disease in a cohort of Angolan pediatric patients. *Mol Biol Rep*. Jul 2020;47(7):5397-5402. doi:10.1007/s11033-020-05628-8
36. Chamouine A, Saandi T, Muszlak M, et al. High fetal hemoglobin level is associated with increased risk of cerebral vasculopathy in children with sickle cell disease in Mayotte. *BMC Pediatr*. Jun 20 2020;20(1):302. doi:10.1186/s12887-020-02187-6

© 2023 Kirkham JK et al. *JAMA Network Open*.

37. Safwat NA, MM EL, Abdel-Wahab SEA, Hamza MT, Boshnak NH, Kenny MA. Polymorphisms of the receptor for advanced glycation end products as vasculopathy predictor in sickle cell disease. *Pediatr Res*. Jan 2021;89(1):185-190. doi:10.1038/s41390-020-1014-3
38. Alaoui-Ismaili FZ, Laghmich A, Ghailani-Nourouti N, Barakat A, Bennani-Mechita M. XmnI Polymorphism in Sickle Cell Disease in North Morocco. *Hemoglobin*. May 2020;44(3):190-194. doi:10.1080/03630269.2020.1772284
39. Nkya S, Mwita L, Mgaya J, et al. Identifying genetic variants and pathways associated with extreme levels of fetal hemoglobin in sickle cell disease in Tanzania. *BMC Med Genet*. Jun 5 2020;21(1):125. doi:10.1186/s12881-020-01059-1
40. Chinedu O, Tonasse WV, Albuquerque DM, et al. Polymorphisms in the heme oxygenase-1 and bone morphogenetic protein receptor type 1b genes and estimated glomerular filtration rate in Brazilian sickle cell anemia patients. *Hematol Transfus Cell Ther*. Apr-Jun 2021;43(2):165-170. doi:10.1016/j.htct.2020.01.009
41. Sales RR, Belisario AR, Faria G, Mendes F, Luizon MR, Viana MB. Functional polymorphisms of BCL11A and HBS1L-MYB genes affect both fetal hemoglobin level and clinical outcomes in a cohort of children with sickle cell anemia. *Ann Hematol*. Jul 2020;99(7):1453-1463. doi:10.1007/s00277-020-04079-2
42. Silva M, Vargas S, Coelho A, et al. Biomarkers and genetic modulators of cerebral vasculopathy in sub-Saharan ancestry children with sickle cell anemia. *Blood Cells Mol Dis*. Jul 2020;83:102436. doi:10.1016/j.bcmd.2020.102436
43. Shaheen I, Khorshied M, Abdel-Raouf R, et al. L-Selectin P213S and Integrin Alpha 2 C807T Genetic Polymorphisms in Pediatric Sickle Cell Disease Patients. *J Pediatr Hematol Oncol*. Nov 2020;42(8):e707-e711. doi:10.1097/MPH.0000000000001839
44. Srisuwananukorn A, Raslan R, Zhang X, et al. Clinical, laboratory, and genetic risk factors for thrombosis in sickle cell disease. *Blood Adv*. May 12 2020;4(9):1978-1986. doi:10.1182/bloodadvances.2019001384
45. Jhun EH, Sadhu N, He Y, et al. S100B single nucleotide polymorphisms exhibit sex-specific associations with chronic pain in sickle cell disease in a largely African-American cohort. *PLoS One*. 2020;15(5):e0232721. doi:10.1371/journal.pone.0232721
46. Alves AC, da Silva VAL, Dos Santos A, et al. Sickle cell anemia in the state of Maranhao: a haplotype study. *Ann Hematol*. Jun 2020;99(6):1225-1230. doi:10.1007/s00277-020-04048-9
47. Alenzi FQ. New mutations of locus control region in Saudi sickle patients. *Saudi J Biol Sci*. May 2020;27(5):1265-1270. doi:10.1016/j.sjbs.2020.01.028
48. Domingos IF, Pereira-Martins DA, Borges-Medeiros RL, et al. Evaluation of oxidative stress-related genetic variants for predicting stroke in patients with sickle cell anemia. *J Neurol Sci*. Jul 15 2020;414:116839. doi:10.1016/j.jns.2020.116839
49. Fong C, Mendoza Y, Barreto G. Genetic variants in the G gamma-globin promoter modulate fetal hemoglobin expression in the Colombian population. *Genet Mol Biol*. 2020;43(2):e20190076. doi:10.1590/1678-4685-GMB-2019-0076
50. Chenou F, Albuquerque DM, Leonardo DP, et al. Endothelial Nitric Oxide Synthase (eNOS) Gene Polymorphisms and Markers of Hemolysis, Inflammation and Endothelial Dysfunction in Brazilian Sickle Cell Anemia Patients. *Biochem Genet*. Aug 2020;58(4):580-594. doi:10.1007/s10528-020-09959-w
51. Sadhu N, Jhun EH, Posen A, et al. Phenylethanolamine N-methyltransferase gene polymorphisms associate with crisis pain in sickle cell disease patients. *Pharmacogenomics*. Mar 2020;21(4):269-278. doi:10.2217/pgs-2019-0096
52. Willen SM, McNeil JB, Rodeghier M, et al. Haptoglobin genotype predicts severe acute vaso-occlusive pain episodes in children with sickle cell anemia. *Am J Hematol*. Apr 2020;95(4):E92-E95. doi:10.1002/ajh.25728
53. Zhang X, Shah BN, Zhang W, et al. S100B has pleiotropic effects on vaso-occlusive manifestations in sickle cell disease. *Am J Hematol*. Mar 2020;95(3):E62-E65. doi:10.1002/ajh.25691
54. Boulassel MR, Al-Zubaidi A, Al-Zadjali S, et al. Elevated levels of circulating invariant natural killer cell subsets are skewed toward Th2-like phenotype in children with sickle cell disease. *Clin Immunol*. Jan 2020;210:108308. doi:10.1016/j.clim.2019.108308

© 2023 Kirkham JK et al. *JAMA Network Open*.

55. Pereira-Martins DA, Coelho-Silva JL, Domingos IF, et al. Association between ANXA2*5681 polymorphism (rs7170178) and osteonecrosis in haemoglobin SS-genotyped patients. *Br J Haematol*. Feb 2020;188(3):e8-e11. doi:10.1111/bjh.16267
56. Powell-Roach KL, Yao Y, Jhun EH, et al. Vasopressin SNP pain factors and stress in sickle cell disease. *PLoS One*. 2019;14(11):e0224886. doi:10.1371/journal.pone.0224886
57. Valente-Frossard TNS, Cruz NRC, Ferreira FO, et al. Polymorphisms in genes that affect the variation of lipid levels in a Brazilian pediatric population with sickle cell disease: rs662799 APOA5 and rs964184 ZPR1. *Blood Cells Mol Dis*. Feb 2020;80:102376. doi:10.1016/j.bcmd.2019.102376
58. Cintho Ozahata M, Page GP, Guo Y, et al. Clinical and Genetic Predictors of Priapism in Sickle Cell Disease: Results from the Recipient Epidemiology and Donor Evaluation Study III Brazil Cohort Study. *J Sex Med*. Dec 2019;16(12):1988-1999. doi:10.1016/j.jsxm.2019.09.012
59. Ndidi US, Adanho CSA, Santiago RP, et al. Effect of N(Epsilon)-(carboxymethyl)lysine on Laboratory Parameters and Its Association with beta (S) Haplotype in Children with Sickle Cell Anemia. *Dis Markers*. 2019;2019:1580485. doi:10.1155/2019/1580485
60. Olatunya OS, Albuquerque DM, Akanbi GO, et al. Uridine diphosphate glucuronosyl transferase 1A (UGT1A1) promoter polymorphism in young patients with sickle cell anaemia: report of the first cohort study from Nigeria. *BMC Med Genet*. Oct 16 2019;20(1):160. doi:10.1186/s12881-019-0899-3
61. Nicolau M, Vargas S, Silva M, et al. Genetic modulators of fetal hemoglobin expression and ischemic stroke occurrence in African descendant children with sickle cell anemia. *Ann Hematol*. Dec 2019;98(12):2673-2681. doi:10.1007/s00277-019-03783-y
62. de Almeida E, Frantz SR, Cesar P, et al. Frequency of Interleukins IL1ss/IL18 and Inflammasome NLRP1/NLRP3 Polymorphisms in Sickle Cell Anemia Patients and their Association with Severity Score. *Curr Mol Med*. 2019;19(10):776-783. doi:10.2174/1566524019666190826143749
63. Meher S, Patel S, Das K, et al. Association of plasma homocysteine level with vaso-occlusive crisis in sickle cell anemia patients of Odisha, India. *Ann Hematol*. Oct 2019;98(10):2257-2265. doi:10.1007/s00277-019-03776-x
64. Cruz PRS, Ananina G, Gil-da-Silva-Lopes VL, et al. Genetic comparison of sickle cell anaemia cohorts from Brazil and the United States reveals high levels of divergence. *Sci Rep*. Jul 26 2019;9(1):10896. doi:10.1038/s41598-019-47313-2
65. Gueye Tall F, Martin C, Ndour EHM, et al. Combined and differential effects of alpha-thalassemia and HbF-quantitative trait loci in Senegalese hydroxyurea-free children with sickle cell anemia. *Pediatr Blood Cancer*. Oct 2019;66(10):e27934. doi:10.1002/pbc.27934
66. Mikobi TM, Lukusa PT, Muamba JM, Rhama T. Homozygous Deletion Alpha-Thalassemia and Hereditary Persistence of Fetal Hemoglobin, Two Genetic Factors Predictive the Reduction of Morbidity and Mortality During Pregnancy in Sickle Cell Patients. A Report from the Democratic Republic of Congo. *Mediterr J Hematol Infect Dis*. 2019;11(1):e2019039. doi:10.4084/MJHID.2019.039
67. Jit BP, Mohanty PK, Pradhan A, et al. Erythrocyte cAMP in Determining Frequency of Acute Pain Episodes in Sickle Cell Disease Patients from Odisha State, India. *Hemoglobin*. Mar 2019;43(2):88-94. doi:10.1080/03630269.2019.1623248
68. Afifi RAA, Sedky YM, Abd EH, Botros SKA. IL-Ibeta+3954 C/T Polymorphism and Its Clinical Associations in Egyptian Sickle Cell Disease Patients. *Int J Hematol Oncol Stem Cell Res*. Jan 1 2019;13(1):35-41.
69. Meinderts SM, Gerritsma JJ, Sins JWR, et al. Identification of genetic biomarkers for alloimmunization in sickle cell disease. *Br J Haematol*. Sep 2019;186(6):887-899. doi:10.1111/bjh.15998
70. Bhagat S, Thakur AS. Influence of beta-Globin Haplotypes on Oxidative Stress, Antioxidant Capacity and Inflammation in Sickle Cell Patients of Chhattisgarh. *Indian J Clin Biochem*. Apr 2019;34(2):201-206. doi:10.1007/s12291-017-0729-3
71. de Carvalho-Siqueira GQ, Ananina G, de Souza BB, et al. Whole-exome sequencing indicates FLG2 variant associated with leg ulcers in Brazilian sickle cell anemia patients. *Exp Biol Med (Maywood)*. Aug 2019;244(11):932-939. doi:10.1177/1535370219849592
72. Bakr S, Khorshied M, Talha N, et al. Implication of HMOX1 and CCR5 genotypes on clinical phenotype of Egyptian patients with sickle cell anemia. *Ann Hematol*. Aug 2019;98(8):1805-1812. doi:10.1007/s00277-019-03697-9

© 2023 Kirkham JK et al. *JAMA Network Open*.

73. Tozatto-Maio K, Girot R, Ly ID, et al. A Toll-like receptor 2 genetic variant modulates occurrence of bacterial infections in patients with sickle cell disease. *Br J Haematol*. Jun 2019;185(5):918-924. doi:10.1111/bjh.15875
74. Zahr RS, Rampersaud E, Kang G, et al. Children with sickle cell anemia and APOL1 genetic variants develop albuminuria early in life. *Haematologica*. Sep 2019;104(9):e385-e387. doi:10.3324/haematol.2018.212779
75. Milton JN, Shaikho EM, Steinberg MH. Haemolysis in sickle cell anaemia: effects of polymorphisms in alpha-globin gene regulatory elements. *Br J Haematol*. Jul 2019;186(2):363-364. doi:10.1111/bjh.15852
76. Jhun EH, Sadhu N, Hu X, et al. Beta2-Adrenergic Receptor Polymorphisms and Haplotypes Associate With Chronic Pain in Sickle Cell Disease. *Front Pharmacol*. 2019;10:84. doi:10.3389/fphar.2019.00084
77. Upadhye D, Jain D, Nadkarni A, Ghosh K, Colah R. Red Cell Indices and Hemoglobin Profile of Newborn Babies with Both the Sickle Gene and Alpha Thalassaemia in Central India. *Indian J Hematol Blood Transfus*. Jan 2019;35(1):109-113. doi:10.1007/s12288-018-0994-1
78. Rezende PV, Belisario AR, Oliveira EL, et al. Co-inheritance of a-thalassemia dramatically decreases the risk of acute splenic sequestration in a large cohort of newborns with hemoglobin SC. *Haematologica*. Jul 2019;104(7):e281-e283. doi:10.3324/haematol.2018.209221
79. Okumura JV, Silva DGH, Torres LS, et al. Atypical beta-S haplotypes: classification and genetic modulation in patients with sickle cell anemia. *J Hum Genet*. Mar 2019;64(3):239-248. doi:10.1038/s10038-018-0554-4
80. Sabrie M, Cannas G, Tazarourte K, et al. Drepa-Opia: A Pilot Study to Determine the Predictive Factors of Morphine Use and Consumption in Hospitalized Adult Patients with Sickle Cell Disease. *Hemoglobin*. Jul 2018;42(4):217-224. doi:10.1080/03630269.2018.1529602
81. Jit BP, Mohanty PK, Purohit P, et al. Association of fetal hemoglobin level with frequency of acute pain episodes in sickle cell disease (HbS-only phenotype) patients. *Blood Cells Mol Dis*. Mar 2019;75:30-34. doi:10.1016/j.bcmd.2018.12.003
82. Williams LM, Qi Z, Batai K, et al. A locus on chromosome 5 shows African ancestry-limited association with alloimmunization in sickle cell disease. *Blood Adv*. Dec 26 2018;2(24):3637-3647. doi:10.1182/bloodadvances.2018020594
83. Hassan FM, Al-Zahrani FM. BCL11A rs1427407 Genotypes in Sickle Cell Anemia Patients Undergo to Stroke Problems in Sudan. *Korean J Fam Med*. Jan 2019;40(1):53-57. doi:10.4082/kjfm.17.0144
84. Dash PM, Sahu PK, Patel S, Mashon RS, Kharat KR, Mukherjee MB. Effect of Assorted Globin Haplotypes and alpha-Thalassemia on the Clinical Heterogeneity of Hb S-beta-Thalassemia. *Hemoglobin*. Jul 2018;42(4):236-242. doi:10.1080/03630269.2018.1536666
85. ElAlfy MS, Ebeid FSE, Kamal TM, Eissa DS, Ismail EAR, Mohamed SH. Angiotensinogen M235T Gene Polymorphism is a Genetic Determinant of Cerebrovascular and Cardiopulmonary Morbidity in Adolescents with Sickle Cell Disease. *J Stroke Cerebrovasc Dis*. Feb 2019;28(2):441-449. doi:10.1016/j.jstrokecerebrovasdis.2018.10.019
86. Antwi-Boasiako C, Dzudzor B, Kudzi W, et al. Association between eNOS Gene Polymorphism (T786C and VNTR) and Sickle Cell Disease Patients in Ghana. *Diseases*. Sep 29 2018;6(4)doi:10.3390/diseases6040090
87. Rooks H, Brewin J, Gardner K, et al. A gain of function variant in PIEZO1 (E756del) and sickle cell disease. *Haematologica*. Mar 2019;104(3):e91-e93. doi:10.3324/haematol.2018.202697
88. Al-Allawi N, Qadir SMA, Puehringer H, Chui DHK, Farrell JJ, Oberkanins C. The association of HBG2, BCL11A, and HMIP polymorphisms with fetal hemoglobin and clinical phenotype in Iraqi Kurds with sickle cell disease. *Int J Lab Hematol*. Feb 2019;41(1):87-93. doi:10.1111/ijlh.12927
89. Ballas SK, Connes P, Investigators of the Multicenter Study of Hydroxyurea in Sickle Cell A. Rheological properties of sickle erythrocytes in patients with sickle-cell anemia: The effect of hydroxyurea, fetal hemoglobin, and alpha-thalassemia. *Eur J Haematol*. Dec 2018;101(6):798-803. doi:10.1111/ejh.13173
90. Alsultan A, Al-Suliman AM, Aleem A, AlGahtani FH, Alfadhel M. Utilizing Whole-Exome Sequencing to Characterize the Phenotypic Variability of Sickle Cell Disease. *Genet Test Mol Biomarkers*. Sep 2018;22(9):561-567. doi:10.1089/gtmb.2018.0058
91. Saraf SL, Viner M, Rischall A, et al. HMOX1 and acute kidney injury in sickle cell anemia. *Blood*. Oct 11 2018;132(15):1621-1625. doi:10.1182/blood-2018-05-853929

© 2023 Kirkham JK et al. *JAMA Network Open*.

92. Olatunya OS, Albuquerque DM, Adekile A, Costa FF. Influence of alpha thalassemia on clinical and laboratory parameters among nigerian children with sickle cell anemia. *J Clin Lab Anal*. Feb 2019;33(2):e22656. doi:10.1002/jcla.22656
93. Marshall K, Howell S, Badaloo A, Reid M, McFarlane-Anderson N, McKenzie C. Exploring putative genetic determinants of inter-individual phenotypic heterogeneity in sickle cell disease: A cross-sectional Jamaican cohort-based study. *Blood Cells Mol Dis*. Nov 2018;73:1-8. doi:10.1016/j.bcmd.2018.08.001
94. Al-Ali ZA, Fallatah RK, Aljaffer EA, et al. ANTXR1 Intronic Variants Are Associated with Fetal Hemoglobin in the Arab-Indian Haplotype of Sickle Cell Disease. *Acta Haematol*. 2018;140(1):55-59. doi:10.1159/000491688
95. Ilboudo Y, Bartolucci P, Garrett ME, et al. A common functional PIEZO1 deletion allele associates with red blood cell density in sickle cell disease patients. *Am J Hematol*. Nov 2018;93(11):E362-E365. doi:10.1002/ajh.25245
96. Jhun EH, Sadhu N, Yao Y, et al. Glucocorticoid receptor single nucleotide polymorphisms are associated with acute crisis pain in sickle cell disease. *Pharmacogenomics*. Aug 1 2018;19(13):1003-1011. doi:10.2217/pgs-2018-0064
97. Sadhu N, Jhun EH, Yao Y, et al. Genetic variants of GCH1 associate with chronic and acute crisis pain in African Americans with sickle cell disease. *Exp Hematol*. Oct 2018;66:42-49. doi:10.1016/j.exphem.2018.07.004
98. Zhang Y, Paikari A, Sumazin P, et al. Metformin induces FOXO3-dependent fetal hemoglobin production in human primary erythroid cells. *Blood*. Jul 19 2018;132(3):321-333. doi:10.1182/blood-2017-11-814335
99. Adeyemo TA, Ojewunmi OO, Oyetunji IA, et al. A survey of genetic fetal-haemoglobin modifiers in Nigerian patients with sickle cell anaemia. *PLoS One*. 2018;13(6):e0197927. doi:10.1371/journal.pone.0197927
100. Jhun EH, Hu X, Sadhu N, et al. Transient receptor potential polymorphism and haplotype associate with crisis pain in sickle cell disease. *Pharmacogenomics*. Apr 2018;19(5):401-411. doi:10.2217/pgs-2017-0198
101. Yahouedehou S, Carvalho MOS, Oliveira RM, et al. Sickle Cell Anemia Patients in Use of Hydroxyurea: Association between Polymorphisms in Genes Encoding Metabolizing Drug Enzymes and Laboratory Parameters. *Dis Markers*. 2018;2018:6105691. doi:10.1155/2018/6105691
102. Raffield LM, Ulirsch JC, Naik RP, et al. Common alpha-globin variants modify hematologic and other clinical phenotypes in sickle cell trait and disease. *PLoS Genet*. Mar 2018;14(3):e1007293. doi:10.1371/journal.pgen.1007293
103. Zhang Y, Belfer I, Nouraie M, et al. Association of genetic variation in COMT gene with pain related to sickle cell disease in patients from the walk-PHaSST study. *J Pain Res*. 2018;11:537-543. doi:10.2147/JPR.S149958
104. Bernaudin F, Arnaud C, Kamdem A, et al. Biological impact of alpha genes, beta haplotypes, and G6PD activity in sickle cell anemia at baseline and with hydroxyurea. *Blood Adv*. Mar 27 2018;2(6):626-637. doi:10.1182/bloodadvances.2017014555
105. Renoux C, Joly P, Faes C, et al. Association between Oxidative Stress, Genetic Factors, and Clinical Severity in Children with Sickle Cell Anemia. *J Pediatr*. Apr 2018;195:228-235. doi:10.1016/j.jpeds.2017.12.021
106. Gardner K, Fulford T, Silver N, et al. g(HbF): a genetic model of fetal hemoglobin in sickle cell disease. *Blood Adv*. Feb 13 2018;2(3):235-239. doi:10.1182/bloodadvances.2017009811
107. Bhanushali AA, Himani K, Patra PK, Das BR. Hb F Levels in Indian Sickle Cell Patients and Association with the HBB Locus Variant rs10128556 (C>T), and the HBG XmnI (Arab-Indian) Variant. *Hemoglobin*. Jul - Nov 2017;41(4-6):317-320. doi:10.1080/03630269.2017.1414059
108. Mwesigwa S, Moulds JM, Chen A, et al. Whole-exome sequencing of sickle cell disease patients with hyperhemolysis syndrome suggests a role for rare variation in disease predisposition. *Transfusion*. Mar 2018;58(3):726-735. doi:10.1111/trf.14431
109. Wonkam A, Mnika K, Ngo Bitoungui VJ, et al. Clinical and genetic factors are associated with pain and hospitalisation rates in sickle cell anaemia in Cameroon. *Br J Haematol*. Jan 2018;180(1):134-146. doi:10.1111/bjh.15011

© 2023 Kirkham JK et al. *JAMA Network Open*.

110. Abou-Elew HH, Youssry I, Hefny S, Hashem RH, Fouad N, Zayed RA. beta(S) globin gene haplotype and the stroke risk among Egyptian children with sickle cell disease. *Hematology*. Jul 2018;23(6):362-367. doi:10.1080/10245332.2017.1403736
111. Howell S, Marshall K, Reid M, McFarlane-Anderson N, McKenzie C. A cross-sectional clinic-based study exploring whether variants within the glutathione S-transferase, haptoglobin and uridine 5'-diphospho-glucuronosyltransferase 1A1 genes are associated with interindividual phenotypic variation in sickle cell anaemia in Jamaica. *Eur J Haematol*. Feb 2018;100(2):147-153. doi:10.1111/ejh.12993
112. Afifi RA, Kamal D, Sayed RE, et al. CD209-336A/G promotor polymorphism and its clinical associations in sickle cell disease Egyptian Pediatric patients. *Hematol Oncol Stem Cell Ther*. Jun 2018;11(2):75-81. doi:10.1016/j.hemonc.2017.09.002
113. Aleluia MM, Fonseca TCC, Souza RQ, et al. Comparative study of sickle cell anemia and hemoglobin SC disease: clinical characterization, laboratory biomarkers and genetic profiles. *BMC Hematol*. 2017;17:15. doi:10.1186/s12878-017-0087-7
114. Meinderts SM, Sins JWR, Fijnvandraat K, et al. Nonclassical FCGR2C haplotype is associated with protection from red blood cell alloimmunization in sickle cell disease. *Blood*. Nov 9 2017;130(19):2121-2130. doi:10.1182/blood-2017-05-784876
115. Rodrigues C, Sell AM, Guelsin GAS, et al. HLA polymorphisms and risk of red blood cell alloimmunisation in polytransfused patients with sickle cell anaemia. *Transfus Med*. Dec 2017;27(6):437-443. doi:10.1111/tme.12459
116. Saraf SL, Akingbola TS, Shah BN, et al. Associations of alpha-thalassemia and BCL11A with stroke in Nigerian, United States, and United Kingdom sickle cell anemia cohorts. *Blood Adv*. Apr 25 2017;1(11):693-698. doi:10.1182/bloodadvances.2017005231
117. Oliveira VB, Dezan MR, Gomes FCA, et al. -318C/T polymorphism of the CTLA-4 gene is an independent risk factor for RBC alloimmunization among sickle cell disease patients. *Int J Immunogenet*. Oct 2017;44(5):219-224. doi:10.1111/iji.12334
118. Santiago RP, Vieira C, Adanho CSA, et al. Laboratory and Genetic Biomarkers Associated with Cerebral Blood Flow Velocity in Hemoglobin SC Disease. *Dis Markers*. 2017;2017:6359871. doi:10.1155/2017/6359871
119. Kormann R, Jannot AS, Narjoz C, et al. Roles of APOL1 G1 and G2 variants in sickle cell disease patients: kidney is the main target. *Br J Haematol*. Oct 2017;179(2):323-335. doi:10.1111/bjh.14842
120. David S, Aguiar P, Antunes L, et al. Variants in the non-coding region of the TLR2 gene associated with infectious subphenotypes in pediatric sickle cell anemia. *Immunogenetics*. Jan 2018;70(1):37-51. doi:10.1007/s00251-017-1013-7
121. Shaikho EM, Farrell JJ, Alsultan A, Sebastiani P, Steinberg MH. Genetic determinants of HbF in Saudi Arabian and African Benin haplotype sickle cell anemia. *Am J Hematol*. Sep 2017;92(9):E555-E557. doi:10.1002/ajh.24822
122. Belisario AR, Sales RR, Toledo NE, Velloso-Rodrigues C, Silva CM, Viana MB. Interleukin-10 haplotypes are not associated with acute cerebral ischemia or high-risk transcranial Doppler in a newborn cohort of 395 children with sickle cell anemia. *Rev Bras Hematol Hemoter*. Apr - Jun 2017;39(2):108-114. doi:10.1016/j.bjhh.2016.09.017
123. de Azevedo LA, Bonazzoni J, Wagner SC, et al. Do Alpha Thalassemia, Fetal Hemoglobin, and the UGT1A1 Polymorphism have an Influence on Serum Bilirubin Levels and Cholelithiasis in Patients with Sickle Cell Disease? *Mol Diagn Ther*. Aug 2017;21(4):437-442. doi:10.1007/s40291-017-0283-y
124. Ilboudo Y, Bartolucci P, Rivera A, et al. Genome-wide association study of erythrocyte density in sickle cell disease patients. *Blood Cells Mol Dis*. Jun 2017;65:60-65. doi:10.1016/j.bcmd.2017.05.005
125. Khorshied MM, Mohamed NS, Hamza RS, Ali RM, El-Ghamrawy MK. Protein Z and Endothelin-1 genetic polymorphisms in pediatric Egyptian sickle cell disease patients. *J Clin Lab Anal*. Feb 2018;32(2)doi:10.1002/jcla.22264
126. Domingos IF, Pereira-Martins DA, Coelho-Silva JL, et al. Interleukin-6 G-174C polymorphism predicts higher risk of stroke in sickle cell anaemia. *Br J Haematol*. Jul 2018;182(2):294-297. doi:10.1111/bjh.14773
127. Pule GD, Bitoungui VJN, Chemegni BC, Kengne AP, Wonkam A. SAR1a promoter polymorphisms are not associated with fetal hemoglobin in patients with sickle cell disease from Cameroon. *BMC Res Notes*. May 12 2017;10(1):183. doi:10.1186/s13104-017-2502-3

© 2023 Kirkham JK et al. *JAMA Network Open*.

128. Geard A, Pule GD, Chetcha Chemegni B, et al. Clinical and genetic predictors of renal dysfunctions in sickle cell anaemia in Cameroon. *Br J Haematol*. Aug 2017;178(4):629-639. doi:10.1111/bjh.14724
129. Aleluia MM, da Guarda CC, Santiago RP, et al. Association of classical markers and establishment of the dyslipidemic sub-phenotype of sickle cell anemia. *Lipids Health Dis*. Apr 11 2017;16(1):74. doi:10.1186/s12944-017-0454-1
130. Mikobi TM, Tshilobo Lukusa P, Aloni MN, et al. Protective BCL11A and HBS1L-MYB polymorphisms in a cohort of 102 Congolese patients suffering from sickle cell anemia. *J Clin Lab Anal*. Jan 2018;32(1)doi:10.1002/jcla.22207
131. Mikobi TM, Lukusa PT, Aloni MN, et al. Association between sickle cell anemia and alpha thalassemia reveals a high prevalence of the alpha(3.7) triplication in congolese patients than in worldwide series. *J Clin Lab Anal*. Jan 2018;32(1)doi:10.1002/jcla.22186
132. Pontes RM, Costa ES, Siqueira PFR, et al. Protector effect of alpha-thalassaemia on cholecystitis and cholecystectomy in sickle cell disease. *Hematology*. Aug 2017;22(7):444-449. doi:10.1080/10245332.2017.1289325
133. Aleluia MM, Santiago RP, da Guarda CC, et al. Genetic modulation of fetal hemoglobin in hydroxyurea-treated sickle cell anemia. *Am J Hematol*. May 2017;92(5):E70-E72. doi:10.1002/ajh.24680
134. da Silva DGH, Belini Junior E, de Souza Torres L, et al. Impact of genetic polymorphisms in key enzymes of homocysteine metabolism on the pathophysiology of sickle cell anemia. *Free Radic Biol Med*. May 2017;106:53-61. doi:10.1016/j.freeradbiomed.2017.02.019
135. Zhang X, Shah BN, Zhang W, et al. A genetic variation associated with plasma erythropoietin and a non-coding transcript of PRKAR1A in sickle cell disease. *Hum Mol Genet*. Oct 15 2016;25(20):4601-4609. doi:10.1093/hmg/ddw299
136. Renoux C, Connes P, Nader E, et al. Alpha-thalassaemia promotes frequent vaso-occlusive crises in children with sickle cell anaemia through haemorheological changes. *Pediatr Blood Cancer*. Aug 2017;64(8)doi:10.1002/pbc.26455
137. Elalfy MS, El Sherif NH, Kamal TM, Aly NH. Klf10 Gene, a Secondary Modifier and a Pharmacogenomic Biomarker of Hydroxyurea Treatment Among Patients With Hemoglobinopathies. *J Pediatr Hematol Oncol*. Apr 2017;39(3):e155-e162. doi:10.1097/MPH.0000000000000762
138. Adekile AD, Akbulut N, Azab AF, Al-Sharida S, Thomas D. The Sickle beta-Thalassemia Phenotype. *J Pediatr Hematol Oncol*. Jul 2017;39(5):327-331. doi:10.1097/MPH.0000000000000747
139. Jacob SA, Novelli EM, Isenberg JS, et al. Thrombospondin-1 gene polymorphism is associated with estimated pulmonary artery pressure in patients with sickle cell anemia. *Am J Hematol*. Mar 2017;92(3):E31-E34. doi:10.1002/ajh.24635
140. Abuamer S, Shome DK, Jaradat A, et al. Frequencies and phenotypic consequences of association of alpha- and beta-thalassemia alleles with sickle-cell disease in Bahrain. *Int J Lab Hematol*. Feb 2017;39(1):76-83. doi:10.1111/ijlh.12577
141. Joly P, Renoux C, Lacan P, et al. UGT1A1 (TA)n genotype is not the major risk factor of cholelithiasis in sickle cell disease children. *Eur J Haematol*. Mar 2017;98(3):296-301. doi:10.1111/ejh.12838
142. Abu-Duhier F, Mir R. GSTT1 (rs4025935) null genotype is associated with increased risk of sickle cell disease in the populations of Tabuk-Northwestern region of Saudi Arabia. *Hematology*. Apr 2017;22(3):172-177. doi:10.1080/10245332.2016.1201631
143. Hu X, Jhun EH, Yao Y, et al. IL1A rs1800587 associates with chronic noncrisis pain in sickle cell disease. *Pharmacogenomics*. Dec 2016;17(18):1999-2006. doi:10.2217/pgs-2016-0085
144. Sippert EA, Visentainer JE, Alves HV, et al. Red blood cell alloimmunization in patients with sickle cell disease: correlation with HLA and cytokine gene polymorphisms. *Transfusion*. Feb 2017;57(2):379-389. doi:10.1111/trf.13920
145. Armenis I, Kalotychou V, Tzanetea R, et al. Prognostic value of T786C and G894T eNOS polymorphisms in sickle cell disease. *Nitric Oxide*. Jan 30 2017;62:17-23. doi:10.1016/j.niox.2016.11.002
146. Kalai M, Dridi M, Chaouch L, et al. The role of rs1984112_G at CD36 gene in increasing reticulocyte level among sickle cell disease patients. *Hematology*. Apr 2017;22(3):178-182. doi:10.1080/10245332.2016.1253253

© 2023 Kirkham JK et al. *JAMA Network Open*.

147. Friedrisch JR, Sheehan V, Flanagan JM, et al. The role of BCL11A and HMIP-2 polymorphisms on endogenous and hydroxyurea induced levels of fetal hemoglobin in sickle cell anemia patients from southern Brazil. *Blood Cells Mol Dis*. Nov 2016;62:32-37. doi:10.1016/j.bcmd.2016.11.002
148. Pule GD, Ngo Bitoungui VJ, Chetcha Chemegni B, Kengne AP, Wonkam A. Studies of novel variants associated with Hb F in Sardinians and Tanzanians in sickle cell disease patients from Cameroon. *Hemoglobin*. Nov 2016;40(6):377-380. doi:10.1080/03630269.2016.1251453
149. Aguiar L, Matos A, Gil A, et al. Sickle cell anemia - Nitric oxide related genetic modifiers of hematological and biochemical parameters. *Clin Hemorheol Microcirc*. 2016;64(4):957-963. doi:10.3233/CH-168008
150. Vathipadiekal V, Farrell JJ, Wang S, et al. A candidate transacting modulator of fetal hemoglobin gene expression in the Arab-Indian haplotype of sickle cell anemia. *Am J Hematol*. Nov 2016;91(11):1118-1122. doi:10.1002/ajh.24527
151. Rodrigues DO, Ribeiro LC, Sudario LC, et al. Genetic determinants and stroke in children with sickle cell disease. *J Pediatr (Rio J)*. Nov - Dec 2016;92(6):602-608. doi:10.1016/j.jped.2016.01.010
152. Schaefer BA, Flanagan JM, Alvarez OA, et al. Genetic Modifiers of White Blood Cell Count, Albuminuria and Glomerular Filtration Rate in Children with Sickle Cell Anemia. *PLoS One*. 2016;11(10):e0164364. doi:10.1371/journal.pone.0164364
153. Martella M, Quaglia N, Frigo AC, Basso G, Colombatti R, Sainati L. Association between a combination of single nucleotide polymorphisms and large vessel cerebral vasculopathy in African children with sickle cell disease. *Blood Cells Mol Dis*. Oct 2016;61:1-3. doi:10.1016/j.bcmd.2016.07.005
154. Saraf SL, Shah BN, Zhang X, et al. APOL1, alpha-thalassemia, and BCL11A variants as a genetic risk profile for progression of chronic kidney disease in sickle cell anemia. *Haematologica*. Jan 2017;102(1):e1-e6. doi:10.3324/haematol.2016.154153
155. Duarte JD, Desai AA, Sysol JR, et al. Genome-Wide Analysis Identifies IL-18 and FUCA2 as Novel Genes Associated with Diastolic Function in African Americans with Sickle Cell Disease. *PLoS One*. 2016;11(9):e0163013. doi:10.1371/journal.pone.0163013
156. Mendonca Belmont TF, do OK, Soares da Silva A, et al. Single Nucleotide Polymorphisms at +191 and +292 of Galectin-3 Gene (LGALS3) Related to Lower GAL-3 Serum Levels Are Associated with Frequent Respiratory Tract Infection and Vaso-Occlusive Crisis in Children with Sickle Cell Anemia. *PLoS One*. 2016;11(9):e0162297. doi:10.1371/journal.pone.0162297
157. Rezende Pdo V, Costa Kda S, Domingues Junior JC, et al. Clinical, hematological and genetic data of a cohort of children with hemoglobin SD. *Rev Bras Hematol Hemoter*. Jul-Sep 2016;38(3):240-6. doi:10.1016/j.bjhh.2016.05.002
158. Belisario AR, Sales RR, Toledo NE, et al. Reticulocyte count is the most important predictor of acute cerebral ischemia and high-risk transcranial Doppler in a newborn cohort of 395 children with sickle cell anemia. *Ann Hematol*. Oct 2016;95(11):1869-80. doi:10.1007/s00277-016-2789-5
159. Verma H, Mishra H, Khodiar PK, Patra PK, Bhaskar LV. NOS3 27-bp and IL4 70-bp VNTR Polymorphisms Do Not Contribute to the Risk of Sickle Cell Crisis. *Turk J Haematol*. Dec 1 2016;33(4):365-366. NOS3 27-bp ve IL4 70-bp VNTR Polimorfizmleri Orak Hucreli Anemide Kriz Riskine Katkida Bulunmaz. doi:10.4274/tjh.2016.0166
160. Dadheech S, Madhulatha D, Jainc S, Joseph J, Jyothy A, Munshi A. Association of BCL11A genetic variant (rs11886868) with severity in beta-thalassaemia major & sickle cell anaemia. *Indian J Med Res*. Apr 2016;143(4):449-54. doi:10.4103/0971-5916.184285
161. Shaikho EM, Habara AH, Alsultan A, et al. Variants of ZBTB7A (LRF) and its beta-globin gene cluster binding motifs in sickle cell anemia. *Blood Cells Mol Dis*. Jul 2016;59:49-51. doi:10.1016/j.bcmd.2016.04.001
162. Moreira JA, Machado RP, Laurentino MR, et al. Influence of betaS-Globin Haplotypes and Hydroxyurea on Arginase I Levels in Sickle Cell Disease. *Dis Markers*. 2016;2016:9172726. doi:10.1155/2016/9172726
163. Upadhye D, Jain D, Trivedi Y, Nadkarni A, Ghosh K, Colah R. Influence of single nucleotide polymorphisms in the BCL11A and HBS1L-MYB gene on the HbF levels and clinical severity of sickle cell anaemia patients. *Ann Hematol*. Jun 2016;95(7):1201-3. doi:10.1007/s00277-016-2675-1
164. Terzi YK, Bulakbasi Balci T, Boga C, et al. Effect of Hereditary Hemochromatosis Gene H63D and C282Y Mutations on Iron Overload in Sickle Cell Disease Patients. *Turk J Haematol*. Dec 1

2016;33(4):320-325. Orak Hucreli Anemi Hastalarinda Herediter Hemokromatozis Geni H63D ve C282Y Mutasyonlarinin Demir Birikimi Uzerindeki Etkisi. doi:10.4274/tjh.2015.0254
165. Chaouch L, Moumni I, Ouragini H, et al. rs11886868 and rs4671393 of BCL11A associated with HbF level variation and modulate clinical events among sickle cell anemia patients. *Hematology*. Aug 2016;21(7):425-9. doi:10.1080/10245332.2015.1107275
166. Cita KC, Ferdinand S, Connes P, et al. Association of adenylyl cyclase 6 rs3730070 polymorphism and hemolytic level in patients with sickle cell anemia. *Blood Cells Mol Dis*. May 2016;58:21-5. doi:10.1016/j.bcmd.2016.02.006
167. Olenscki Gilli SC, Pericole FV, Benites BD, et al. Cytokine polymorphisms in sickle cell disease and the relationship with cytokine expression. *Exp Hematol*. Jul 2016;44(7):583-9. doi:10.1016/j.exphem.2016.03.008
168. Liu L, Pertsemlidis A, Ding LH, et al. Original Research: A case-control genome-wide association study identifies genetic modifiers of fetal hemoglobin in sickle cell disease. *Exp Biol Med (Maywood)*. Apr 2016;241(7):706-18. doi:10.1177/1535370216642047
169. Zachariah M, Al Zadjali S, Bashir W, et al. Impact of Mannose-Binding Protein Gene Polymorphisms in Omani Sickle Cell Disease Patients. *Mediterr J Hematol Infect Dis*. 2016;8(1):e2016013. doi:10.4084/MJHID.2016.013
170. Okumura JV, Silva DG, Torres LS, et al. Inheritance of the Bantu/Benin haplotype causes less severe hemolytic and oxidative stress in sickle cell anemia patients treated with hydroxycarbamide. *J Hum Genet*. Jul 2016;61(7):605-11. doi:10.1038/jhg.2016.16
171. Torres Lde S, Okumura JV, da Silva DG, et al. Plasma levels of TGF-beta1 in homeostasis of the inflammation in sickle cell disease. *Cytokine*. Apr 2016;80:18-25. doi:10.1016/j.cyto.2016.02.012
172. Farawela HM, El-Ghamrawy M, Farhan MS, Soliman R, Yousry SM, AbdelRahman HA. Association between Duffy antigen receptor expression and disease severity in sickle cell disease patients. *Hematology*. Sep 2016;21(8):474-9. doi:10.1080/10245332.2015.1111643
173. Yousry SM, Ellithy HN, Shahin GH. Endothelial nitric oxide synthase gene polymorphisms and the risk of vasculopathy in sickle cell disease. *Hematology*. Jul 2016;21(6):359-67. doi:10.1080/10245332.2016.1142710
174. Leonardo FC, Brugnerotto AF, Domingos IF, et al. Reduced rate of sickle-related complications in Brazilian patients carrying HbF-promoting alleles at the BCL11A and HMIP-2 loci. *Br J Haematol*. May 2016;173(3):456-60. doi:10.1111/bjh.13961
175. Moumni I, Ben Mustapha M, Ben Mansour I, et al. Fetal Hemoglobin in Tunisian Sickle Cell Disease Patient: Relationship with Polymorphic Sequences Cis to the beta-Globin Gene. *Indian J Hematol Blood Transfus*. Mar 2016;32(1):114-9. doi:10.1007/s12288-015-0504-7
176. Fong C, Menzel S, Lizarralde MA, Barreto G. Genetic variants associated with fetal hemoglobin levels show the diverse ethnic origin in Colombian patients with sickle cell anemia. *Biomedica*. Jul-Sep 2015;35(3):437-43. doi:10.7705/biomedica.v35i3.2573
177. Belisario AR, Rodrigues Sales R, Evelin Toledo N, Velloso-Rodrigues C, Maria Silva C, Borato Viana M. Glucose-6-Phosphate Dehydrogenase Deficiency in Brazilian Children With Sickle Cell Anemia is not Associated With Clinical Ischemic Stroke or High-Risk Transcranial Doppler. *Pediatr Blood Cancer*. Jun 2016;63(6):1046-9. doi:10.1002/pbc.25924
178. Upadhye DS, Jain DL, Trivedi YL, Nadkarni AH, Ghosh K, Colah RB. Neonatal Screening and the Clinical Outcome in Children with Sickle Cell Disease in Central India. *PLoS One*. 2016;11(1):e0147081. doi:10.1371/journal.pone.0147081
179. Mousa SM, El-Ghamrawy MK, Gouda H, Khorshied M, El-Salam Ahmed DA, Shiba H. Prevalence of Hepatitis C among Egyptian Children with Sickle Cell Disease and the Role of IL28b Gene Polymorphisms in Spontaneous Viral Clearance. *Mediterr J Hematol Infect Dis*. 2016;8(1):e2016007. doi:10.4084/MJHID.2016.007
180. Sommet J, Alberti C, Couque N, et al. Clinical and haematological risk factors for cerebral macrovasculopathy in a sickle cell disease newborn cohort: a prospective study. *Br J Haematol*. Mar 2016;172(6):966-77. doi:10.1111/bjh.13916
181. Alkindi SY, Pathare A, Al Zadjali S, et al. Serum Total Bilirubin, not Cholelithiasis, is Influenced by UGT1A1 Polymorphism, Alpha Thalassemia and beta(s) Haplotype: First Report on Comparison between Arab-Indian and African beta(s) Genes. *Mediterr J Hematol Infect Dis*. 2015;7(1):e2015060. doi:10.4084/MJHID.2015.060

© 2023 Kirkham JK et al. *JAMA Network Open*.

182. Damy T, Bodez D, Habibi A, et al. Haematological determinants of cardiac involvement in adults with sickle cell disease. *Eur Heart J*. Apr 7 2016;37(14):1158-1167. doi:10.1093/eurheartj/ehv555
183. Shimauti EL, Silva DG, de Souza EM, de Almeida EA, Leal FP, Bonini-Domingos CR. Prevalence of beta(S)-globin gene haplotypes, alpha-thalassemia (3.7 kb deletion) and redox status in patients with sickle cell anemia in the state of Parana, Brazil. *Genet Mol Biol*. Jul-Sep 2015;38(3):316-23. doi:10.1590/S1415-475738320140231
184. Tatari-Calderone Z, Gordish-Dressman H, Fasano R, et al. Protective effect of HLA-DQB1 alleles against alloimmunization in patients with sickle cell disease. *Hum Immunol*. Jan 2016;77(1):35-40. doi:10.1016/j.humimm.2015.10.010
185. da Fonseca SF, Amorim T, Purificacao A, Goncalves M, Boa-Sorte N. Hemoglobin A2 values in sickle cell disease patients quantified by high performance liquid chromatography and the influence of alpha thalassemia. *Rev Bras Hematol Hemoter*. Sep-Oct 2015;37(5):296-301. doi:10.1016/j.bjhh.2015.05.005
186. Pule GD, Ngo Bitoungui VJ, Chetcha Chemegni B, Kengne AP, Antonarakis S, Wonkam A. Association between Variants at BCL11A Erythroid-Specific Enhancer and Fetal Hemoglobin Levels among Sickle Cell Disease Patients in Cameroon: Implications for Future Therapeutic Interventions. *OMICS*. Oct 2015;19(10):627-31. doi:10.1089/omi.2015.0124
187. Belisario AR, Sales RR, Toledo NE, Velloso-Rodrigues C, Silva CM, Viana MB. Association between ENPP1 K173Q and stroke in a newborn cohort of 395 Brazilian children with sickle cell anemia. *Blood*. Sep 3 2015;126(10):1259-60. doi:10.1182/blood-2015-05-645176
188. Saraf SL, Zhang X, Shah B, et al. Genetic variants and cell-free hemoglobin processing in sickle cell nephropathy. *Haematologica*. Oct 2015;100(10):1275-84. doi:10.3324/haematol.2015.124875
189. Chaouch L, Kalai M, Darragi I, et al. Genetic link with cholelithiasis among pediatric SCA Tunisian patients: Examples of UGT1A1, SLCO1A2 and SLCO1B1. *Hematology*. Mar 2016;21(2):121-5. doi:10.1179/1607845415Y.0000000030
190. Ferdinand S, Connes P, Brudey L, et al. Impact of eNOS polymorphisms on red blood cell aggregation in sickle cell disease. *Blood Cells Mol Dis*. Aug 2015;55(2):151-3. doi:10.1016/j.bcmd.2015.05.008
191. Lubega I, Ndugwa CM, Mworozi EA, Tumwine JK. Alpha thalassemia among sickle cell anaemia patients in Kampala, Uganda. *Afr Health Sci*. Jun 2015;15(2):682-9. doi:10.4314/ahs.v15i2.48
192. Joly P, Garnier N, Kebaili K, et al. G6PD deficiency and absence of alpha-thalassemia increase the risk for cerebral vasculopathy in children with sickle cell anemia. *Eur J Haematol*. Apr 2016;96(4):404-8. doi:10.1111/ejh.12607
193. Zhang X, Zhang W, Saraf SL, et al. Genetic polymorphism of APOB is associated with diabetes mellitus in sickle cell disease. *Hum Genet*. Aug 2015;134(8):895-904. doi:10.1007/s00439-015-1572-3
194. Mtatiro SN, Mgaya J, Singh T, et al. Genetic association of fetal-hemoglobin levels in individuals with sickle cell disease in Tanzania maps to conserved regulatory elements within the MYB core enhancer. *BMC Med Genet*. Feb 10 2015;16:4. doi:10.1186/s12881-015-0148-3
195. Ellithy HN, Yousri S, Shahin GH. Relation between glutathione S-transferase genes (GSTM1, GSTT1, and GSTP1) polymorphisms and clinical manifestations of sickle cell disease in Egyptian patients. *Hematology*. Dec 2015;20(10):598-606. doi:10.1179/1607845415Y.0000000013
196. Adekile A, Menzel S, Gupta R, et al. Response to hydroxyurea among Kuwaiti patients with sickle cell disease and elevated baseline HbF levels. *Am J Hematol*. Jul 2015;90(7):E138-9. doi:10.1002/ajh.24027
197. Muszlak M, Pissard S, Badens C, Chamouine A, Maillard O, Thuret I. Genetic Modifiers of Sickle Cell Disease: A Genotype-Phenotype Relationship Study in a Cohort of 82 Children on Mayotte Island. *Hemoglobin*. 2015;39(3):156-61. doi:10.3109/03630269.2015.1023897
198. Sebastiani P, Farrell JJ, Alsultan A, et al. BCL11A enhancer haplotypes and fetal hemoglobin in sickle cell anemia. *Blood Cells Mol Dis*. Mar 2015;54(3):224-30. doi:10.1016/j.bcmd.2015.01.001
199. Hanchard NA, Moulds JM, Belmont JW, Chen A. A Genome-Wide Screen for Large-Effect Alloimmunization Susceptibility Loci among Red Blood Cell Transfusion Recipients with Sickle Cell Disease. *Transfus Med Hemother*. Nov 2014;41(6):453-61. doi:10.1159/000369079
200. Bhanushali AA, Patra PK, Pradhan S, Khanka SS, Singh S, Das BR. Genetics of fetal hemoglobin in tribal Indian patients with sickle cell anemia. *Transl Res*. Jun 2015;165(6):696-703. doi:10.1016/j.trsl.2015.01.002

© 2023 Kirkham JK et al. *JAMA Network Open*.

201. Vicari P, Adegoke SA, Mazzotti DR, Cancado RD, Nogutti MA, Figueiredo MS. Interleukin-1beta and interleukin-6 gene polymorphisms are associated with manifestations of sickle cell anemia. *Blood Cells Mol Dis*. Mar 2015;54(3):244-9. doi:10.1016/j.bcmd.2014.12.004
202. Bitoungui VJ, Ngogang J, Wonkam A. Polymorphism at BCL11A compared to HBS1L-MYB loci explains less of the variance in HbF in patients with sickle cell disease in Cameroon. *Blood Cells Mol Dis*. Mar 2015;54(3):268-9. doi:10.1016/j.bcmd.2014.11.010
203. Bhanushali AA, Patra PK, Nair D, Verma H, Das BR. Genetic variant in the BCL11A (rs1427407), but not HBS1-MYB (rs6934903) loci associate with fetal hemoglobin levels in Indian sickle cell disease patients. *Blood Cells Mol Dis*. Jan 2015;54(1):4-8. doi:10.1016/j.bcmd.2014.10.003
204. Mtatiro SN, Singh T, Rooks H, et al. Genome wide association study of fetal hemoglobin in sickle cell anemia in Tanzania. *PLoS One*. 2014;9(11):e111464. doi:10.1371/journal.pone.0111464
205. Camilo-Araujo RF, Amancio OM, Figueiredo MS, Cabanas-Pedro AC, Braga JA. Molecular analysis and association with clinical and laboratory manifestations in children with sickle cell anemia. *Rev Bras Hematol Hemoter*. Sep-Oct 2014;36(5):334-9. doi:10.1016/j.bjhh.2014.06.002
206. Tantawy AA, Adly AA, Ismail EA, Aly SH. Endothelial nitric oxide synthase gene intron 4 VNTR polymorphism in sickle cell disease: relation to vasculopathy and disease severity. *Pediatr Blood Cancer*. Mar 2015;62(3):389-94. doi:10.1002/pbc.25234
207. Mtatiro SN, Makani J, Mmbando B, Thein SL, Menzel S, Cox SE. Genetic variants at HbF-modifier loci moderate anemia and leukocytosis in sickle cell disease in Tanzania. *Am J Hematol*. Jan 2015;90(1):E1-4. doi:10.1002/ajh.23859
208. Belisario AR, Nogueira FL, Rodrigues RS, et al. Association of alpha-thalassemia, TNF-alpha (-308G>A) and VCAM-1 (c.1238G>C) gene polymorphisms with cerebrovascular disease in a newborn cohort of 411 children with sickle cell anemia. *Blood Cells Mol Dis*. Jan 2015;54(1):44-50. doi:10.1016/j.bcmd.2014.08.001
209. Redha NA, Mahdi N, Al-Habboubi HH, Almawi WY. Impact of VEGFA -583C > T polymorphism on serum VEGF levels and the susceptibility to acute chest syndrome in pediatric patients with sickle cell disease. *Pediatr Blood Cancer*. Dec 2014;61(12):2310-2. doi:10.1002/pbc.25158
210. Abdel Rahman HA, Abou-Elew HH, El-Shorbagy RM, Fawzy R, Youssry I. Influence of iron regulating genes mutations on iron status in Egyptian patients with sickle cell disease. *Ann Clin Lab Sci*. Summer 2014;44(3):304-9.
211. Jhun E, He Y, Yao Y, Molokie RE, Wilkie DJ, Wang ZJ. Dopamine D3 receptor Ser9Gly and catechol-o-methyltransferase Val158Met polymorphisms and acute pain in sickle cell disease. *Anesth Analg*. Nov 2014;119(5):1201-7. doi:10.1213/ANE.0000000000000382
212. Cardoso GL, Diniz IG, Silva AN, et al. DNA polymorphisms at BCL11A, HBS1L-MYB and Xmn1-HBG2 site loci associated with fetal hemoglobin levels in sickle cell anemia patients from Northern Brazil. *Blood Cells Mol Dis*. Dec 2014;53(4):176-9. doi:10.1016/j.bcmd.2014.07.006
213. Griffin PJ, Sebastiani P, Edward H, et al. The genetics of hemoglobin A2 regulation in sickle cell anemia. *Am J Hematol*. Nov 2014;89(11):1019-23. doi:10.1002/ajh.23811
214. Silva IV, Reis AF, Palare MJ, Ferrao A, Rodrigues T, Morais A. Sickle cell disease in children: chronic complications and search of predictive factors for adverse outcomes. *Eur J Haematol*. Feb 2015;94(2):157-61. doi:10.1111/ejh.12411
215. Resende Cardoso PS, Lopes Pessoa de Aguiar RA, Viana MB. Clinical complications in pregnant women with sickle cell disease: prospective study of factors predicting maternal death or near miss. *Rev Bras Hematol Hemoter*. Jul-Aug 2014;36(4):256-63. doi:10.1016/j.bjhh.2014.05.007
216. Rumaney MB, Ngo Bitoungui VJ, Vorster AA, et al. The co-inheritance of alpha-thalassemia and sickle cell anemia is associated with better hematological indices and lower consultations rate in Cameroonian patients and could improve their survival. *PLoS One*. 2014;9(6):e100516. doi:10.1371/journal.pone.0100516
217. Shiba HF, El-Ghamrawy MK, Shaheen IA, Ali RA, Mousa SM. Glutathione S-transferase gene polymorphisms (GSTM1, GSTT1, and GSTP1) in Egyptian pediatric patients with sickle cell disease. *Pediatr Dev Pathol*. Jul-Aug 2014;17(4):265-70. doi:10.2350/14-03-1452-OA.1
218. Laurentino MR, Maia PAF, Barbosa MC, Bandeira IC, Rocha LB, Goncalves RP. Influence of betaS-globin haplotypes and hydroxyurea on tumor necrosis factor-alpha levels in sickle cell anemia. *Rev Bras Hematol Hemoter*. Mar 2014;36(2):121-5. doi:10.5581/1516-8484.20140028

© 2023 Kirkham JK et al. *JAMA Network Open*.

219. Wonkam A, Ngo Bitoungui VJ, Vorster AA, et al. Association of variants at BCL11A and HBS1L-MYB with hemoglobin F and hospitalization rates among sickle cell patients in Cameroon. *PLoS One*. 2014;9(3):e92506. doi:10.1371/journal.pone.0092506
220. Cox SE, Makani J, Soka D, et al. Haptoglobin, alpha-thalassaemia and glucose-6-phosphate dehydrogenase polymorphisms and risk of abnormal transcranial Doppler among patients with sickle cell anaemia in Tanzania. *Br J Haematol*. Jun 2014;165(5):699-706. doi:10.1111/bjh.12791
221. Wonkam A, Rumaney MB, Ngo Bitoungui VJ, Vorster AA, Ramesar R, Ngogang J. Coinheritance of sickle cell anemia and alpha-thalassemia delays disease onset and could improve survival in Cameroonian's patients (Sub-Saharan Africa). *Am J Hematol*. Jun 2014;89(6):664-5. doi:10.1002/ajh.23711
222. Connes P, Lamarre Y, Waltz X, et al. Haemolysis and abnormal haemorheology in sickle cell anaemia. *Br J Haematol*. May 2014;165(4):564-72. doi:10.1111/bjh.12786
223. Milton JN, Gordeuk VR, Taylor JGt, Gladwin MT, Steinberg MH, Sebastiani P. Prediction of fetal hemoglobin in sickle cell anemia using an ensemble of genetic risk prediction models. *Circ Cardiovasc Genet*. Apr 2014;7(2):110-5. doi:10.1161/CIRCGENETICS.113.000387
224. Barbosa LC, Miranda-Vilela AL, Hiragi Cde O, et al. Haptoglobin and myeloperoxidase (- G463A) gene polymorphisms in Brazilian sickle cell patients with and without secondary iron overload. *Blood Cells Mol Dis*. Feb-Mar 2014;52(2-3):95-107. doi:10.1016/j.bcmd.2013.10.001
225. Zhang X, Zhang W, Ma SF, et al. Hypoxic response contributes to altered gene expression and precapillary pulmonary hypertension in patients with sickle cell disease. *Circulation*. Apr 22 2014;129(16):1650-8. doi:10.1161/CIRCULATIONAHA.113.005296
226. Domingos IF, Falcao DA, Hatzlhofer BL, et al. Influence of the betas haplotype and alpha-thalassemia on stroke development in a Brazilian population with sickle cell anaemia. *Ann Hematol*. Jul 2014;93(7):1123-9. doi:10.1007/s00277-014-2016-1
227. Dadheech S, Jain S, Madhulatha D, et al. Association of Xmn1 -158 gammaG variant with severity and HbF levels in beta-thalassemia major and sickle cell anaemia. *Mol Biol Rep*. May 2014;41(5):3331-7. doi:10.1007/s11033-014-3195-5
228. Pandey SK, Pandey S, Ranjan R, et al. Phenotypic effect of alpha-globin gene numbers on Indian sickle beta-thalassemia patients. *J Clin Lab Anal*. Mar 2014;28(2):110-3. doi:10.1002/jcla.21652
229. Bandeira IC, Rocha LB, Barbosa MC, et al. Chronic inflammatory state in sickle cell anemia patients is associated with HBB(*)S haplotype. *Cytokine*. Feb 2014;65(2):217-21. doi:10.1016/j.cyto.2013.10.009
230. Chaouch L, Kalai M, Jbara MB, et al. Association between rs267196 and rs267201 of BMP6 gene and osteonecrosis among sickle cell aneamia patients. *Biomed Pap Med Fac Univ Palacky Olomouc Czech Repub*. Mar 2015;159(1):145-9. doi:10.5507/bp.2013.080
231. Patel DK, Purohit P, Dehury S, et al. Fetal hemoglobin and alpha thalassemia modulate the phenotypic expression of HbSD-Punjab. *Int J Lab Hematol*. Aug 2014;36(4):444-50. doi:10.1111/ijlh.12165
232. AlFadhli S, Al-Jafer H, Hadi M, Al-Mutairi M, Nizam R. The effect of UGT1A1 promoter polymorphism in the development of hyperbilirubinemia and cholelithiasis in hemoglobinopathy patients. *PLoS One*. 2013;8(10):e77681. doi:10.1371/journal.pone.0077681
233. Coelho A, Dias A, Morais A, et al. Genetic variation in CD36, HBA, NOS3 and VCAM1 is associated with chronic haemolysis level in sickle cell anaemia: a longitudinal study. *Eur J Haematol*. Mar 2014;92(3):237-43. doi:10.1111/ejh.12226
234. Chaouch L, Talbi E, Moumni I, et al. Early complication in sickle cell anemia children due to A(TA)nTAA polymorphism at the promoter of UGT1A1 gene. *Dis Markers*. 2013;35(2):67-72. doi:10.1155/2013/173474
235. Belfer I, Youngblood V, Darbari DS, et al. A GCH1 haplotype confers sex-specific susceptibility to pain crises and altered endothelial function in adults with sickle cell anemia. *Am J Hematol*. Feb 2014;89(2):187-93. doi:10.1002/ajh.23613
236. Silva DG, Belini Junior E, Carrocini GC, et al. Genetic and biochemical markers of hydroxyurea therapeutic response in sickle cell anemia. *BMC Med Genet*. Oct 9 2013;14:108. doi:10.1186/1471-2350-14-108
237. Nishank SS, Singh MP, Yadav R, Gupta RB, Gadge VS, Gwal A. Endothelial nitric oxide synthase gene polymorphism is associated with sickle cell disease patients in India. *J Hum Genet*. Dec 2013;58(12):775-9. doi:10.1038/jhg.2013.99

© 2023 Kirkham JK et al. *JAMA Network Open*.

238. Hamad Z, Aljedai A, Halwani R, AlSultan A. UGT1A1 promoter polymorphism associated with serum bilirubin level in Saudi patients with sickle cell disease. *Ann Saudi Med*. Jul-Aug 2013;33(4):372-6. doi:10.5144/0256-4947.2013.372
239. Bhatnagar P, Barron-Casella E, Bean CJ, et al. Genome-wide meta-analysis of systolic blood pressure in children with sickle cell disease. *PLoS One*. 2013;8(9):e74193. doi:10.1371/journal.pone.0074193
240. Lamarre Y, Romana M, Lemonne N, et al. Alpha thalassemia protects sickle cell anemia patients from macro-albuminuria through its effects on red blood cell rheological properties. *Clin Hemorheol Microcirc*. 2014;57(1):63-72. doi:10.3233/CH-131772
241. Nishank SS, Singh MP, Yadav R. Clinical impact of factor V Leiden, prothrombin G20210A, and MTHFR C677T mutations among sickle cell disease patients of Central India. *Eur J Haematol*. Nov 2013;91(5):462-6. doi:10.1111/ejh.12190
242. Bean CJ, Boulet SL, Yang G, et al. Acute chest syndrome is associated with single nucleotide polymorphism-defined beta globin cluster haplotype in children with sickle cell anaemia. *Br J Haematol*. Oct 2013;163(2):268-76. doi:10.1111/bjh.12507
243. Rocha LB, da Silva Jn GB, Daher Ede F, Rocha HA, Elias DB, Goncalves RP. Kidney dysfunction and beta S-haplotypes in patients with sickle cell disease. *Rev Bras Hematol Hemoter*. 2013;35(3):171-3. doi:10.5581/1516-8484.20130052
244. Kalai M, Chaouch L, Mansour IB, Hafsia R, Ghanem A, Abbes S. Frequency of three polymorphisms of the CCL5 gene (rs2107538, rs2280788 and rs2280789) and their implications for the phenotypic expression of sickle cell anemia in Tunisia. *Pol J Pathol*. Jun 2013;64(2):84-9. doi:10.5114/pjp.2013.36012
245. Barbosa CG, Aleluia AC, Pacheco AP, et al. Genetic modulation of HbF in Brazilians with HbSC disease and sickle cell anemia. *Am J Hematol*. Oct 2013;88(10):923-4. doi:10.1002/ajh.23538
246. Nishank SS. Endothelial Nitric Oxide Synthase (eNOS) Gene Polymorphism is Associated with Age Onset of Menarche in Sickle Cell Disease Females of India. *Mediterr J Hematol Infect Dis*. 2013;5(1):e2013036. doi:10.4084/MJHID.2013.036
247. Tatari-Calderone Z, Tamouza R, Le Bouder GP, et al. The association of CD81 polymorphisms with alloimmunization in sickle cell disease. *Clin Dev Immunol*. 2013;2013:937846. doi:10.1155/2013/937846
248. Gil GP, Ananina G, Oliveira MB, et al. Polymorphism in the HMOX1 gene is associated with high levels of fetal hemoglobin in Brazilian patients with sickle cell anemia. *Hemoglobin*. 2013;37(4):315-24. doi:10.3109/03630269.2013.789438
249. Galarneau G, Coady S, Garrett ME, et al. Gene-centric association study of acute chest syndrome and painful crisis in sickle cell disease patients. *Blood*. Jul 18 2013;122(3):434-42. doi:10.1182/blood-2013-01-478776
250. Cox SE, Makani J, Newton CR, Prentice AM, Kirkham FJ. Hematological and Genetic Predictors of Daytime Hemoglobin Saturation in Tanzanian Children with and without Sickle Cell Anemia. *ISRN Hematol*. 2013;2013:472909. doi:10.1155/2013/472909
251. Sheehan VA, Luo Z, Flanagan JM, et al. Genetic modifiers of sickle cell anemia in the BABY HUG cohort: influence on laboratory and clinical phenotypes. *Am J Hematol*. Jul 2013;88(7):571-6. doi:10.1002/ajh.23457
252. de Oliveira Filho RA, Silva GJ, de Farias Domingos I, et al. Association between the genetic polymorphisms of glutathione S-transferase (GSTM1 and GSTT1) and the clinical manifestations in sickle cell anemia. *Blood Cells Mol Dis*. Aug 2013;51(2):76-9. doi:10.1016/j.bcmd.2013.03.003
253. Alsultan A, Ngo D, Bae H, et al. Genetic studies of fetal hemoglobin in the Arab-Indian haplotype sickle cell-beta(0) thalassemia. *Am J Hematol*. Jun 2013;88(6):531-2. doi:10.1002/ajh.23434
254. Ngo D, Bae H, Steinberg MH, et al. Fetal hemoglobin in sickle cell anemia: genetic studies of the Arab-Indian haplotype. *Blood Cells Mol Dis*. Jun 2013;51(1):22-6. doi:10.1016/j.bcmd.2012.12.005
255. Flanagan JM, Sheehan V, Linder H, et al. Genetic mapping and exome sequencing identify 2 mutations associated with stroke protection in pediatric patients with sickle cell anemia. *Blood*. Apr 18 2013;121(16):3237-45. doi:10.1182/blood-2012-10-464156
256. Green NS, Ender KL, Pashankar F, et al. Candidate sequence variants and fetal hemoglobin in children with sickle cell disease treated with hydroxyurea. *PLoS One*. 2013;8(2):e55709. doi:10.1371/journal.pone.0055709

© 2023 Kirkham JK et al. *JAMA Network Open*.

257. Milton JN, Rooks H, Drasar E, et al. Genetic determinants of haemolysis in sickle cell anaemia. *Br J Haematol*. Apr 2013;161(2):270-8. doi:10.1111/bjh.12245
258. Carvalho-dos Santos BS, Dias-Elias DB, da Silva-Rocha LB, Cavalcante-Barbosa M, Pinheiro-Goncalves R. Impact of beta(S)-globin haplotypes on oxidative stress in patients with sickle cell anemia in steady state. *Arch Med Res*. Oct 2012;43(7):536-40. doi:10.1016/j.arcmed.2012.08.014
259. Cox SE, L'Esperance V, Makani J, et al. Sickle cell anemia: iron availability and nocturnal oximetry. *J Clin Sleep Med*. Oct 15 2012;8(5):541-5. doi:10.5664/jcsm.2152
260. Nekhai S, Xu M, Foster A, et al. Reduced sensitivity of the ferroportin Q248H mutant to physiological concentrations of hepcidin. *Haematologica*. Mar 2013;98(3):455-63. doi:10.3324/haematol.2012.066530
261. da Silva Filho IL, Ribeiro GS, Moura PG, Vechi ML, Cavalcante AC, de Andrada-Serpa MJ. Sickle cell disease: acute clinical manifestations in early childhood and molecular characteristics in a group of children in Rio de Janeiro. *Rev Bras Hematol Hemoter*. 2012;34(3):196-201. doi:10.5581/1516-8484.20120049
262. Haghpanah S, Nasirabadi S, Kianmehr M, Afrasiabi A, Karimi M. Polymorphisms associated with sickle cell disease in Southern Iran. *Genetika*. Jul 2012;48(7):890-3.
263. Nouraie M, Lee JS, Zhang Y, et al. The relationship between the severity of hemolysis, clinical manifestations and risk of death in 415 patients with sickle cell anemia in the US and Europe. *Haematologica*. Mar 2013;98(3):464-72. doi:10.3324/haematol.2012.068965
264. Bean CJ, Boulet SL, Ellingsen D, et al. Heme oxygenase-1 gene promoter polymorphism is associated with reduced incidence of acute chest syndrome among children with sickle cell disease. *Blood*. Nov 1 2012;120(18):3822-8. doi:10.1182/blood-2011-06-361642
265. Thangarajh M, Yang G, Fuchs D, et al. Magnetic resonance angiography-defined intracranial vasculopathy is associated with silent cerebral infarcts and glucose-6-phosphate dehydrogenase mutation in children with sickle cell anaemia. *Br J Haematol*. Nov 2012;159(3):352-9. doi:10.1111/bjh.12034
266. Bae HT, Baldwin CT, Sebastiani P, et al. Meta-analysis of 2040 sickle cell anemia patients: BCL11A and HBS1L-MYB are the major modifiers of HbF in African Americans. *Blood*. Aug 30 2012;120(9):1961-2. doi:10.1182/blood-2012-06-432849
267. Al-Habboubi HH, Mahdi N, Abu-Hijleh TM, Abu-Hijleh FM, Sater MS, Almawi WY. The relation of vascular endothelial growth factor (VEGF) gene polymorphisms on VEGF levels and the risk of vasoocclusive crisis in sickle cell disease. *Eur J Haematol*. Nov 2012;89(5):403-9. doi:10.1111/ejh.12003
268. Hatzlhofer BL, Bezerra MA, Santos MN, et al. MTHFR polymorphic variant C677T is associated to vascular complications in sickle-cell disease. *Genet Test Mol Biomarkers*. Sep 2012;16(9):1038-43. doi:10.1089/gtmb.2011.0361
269. Bartolucci P, Brugnara C, Teixeira-Pinto A, et al. Erythrocyte density in sickle cell syndromes is associated with specific clinical manifestations and hemolysis. *Blood*. Oct 11 2012;120(15):3136-41. doi:10.1182/blood-2012-04-424184
270. Desai AA, Zhou T, Ahmad H, et al. A novel molecular signature for elevated tricuspid regurgitation velocity in sickle cell disease. *Am J Respir Crit Care Med*. Aug 15 2012;186(4):359-68. doi:10.1164/rccm.201201-0057OC
271. Mahdi N, Abu-Hijleh TM, Abu-Hijleh FM, Sater MS, Al-Ola K, Almawi WY. Protein Z polymorphisms associated with vaso-occlusive crisis in young sickle cell disease patients. *Ann Hematol*. Aug 2012;91(8):1215-20. doi:10.1007/s00277-012-1474-6
272. Milton JN, Sebastiani P, Solovieff N, et al. A genome-wide association study of total bilirubin and cholelithiasis risk in sickle cell anemia. *PLoS One*. 2012;7(4):e34741. doi:10.1371/journal.pone.0034741
273. Alsultan A, Aleem A, Ghabbour H, et al. Sickle cell disease subphenotypes in patients from Southwestern Province of Saudi Arabia. *J Pediatr Hematol Oncol*. Mar 2012;34(2):79-84. doi:10.1097/MPH.0b013e3182422844
274. Viana-Baracioli LM, Tukamoto Junior NC, Ricci Junior O, Mattos LC, Angulo IL, Bonini-Domingos CR. Comparison of oxidative stress and the frequency of polymorphisms in the HFE gene between hemoglobin S trait blood donors and sickle cell disease patients. *Genet Mol Res*. Dec 8 2011;10(4):3446-54. doi:10.4238/2011.December.8.4

© 2023 Kirkham JK et al. *JAMA Network Open*.

275. Akinsheye I, Solovieff N, Ngo D, et al. Fetal hemoglobin in sickle cell anemia: molecular characterization of the unusually high fetal hemoglobin phenotype in African Americans. *Am J Hematol*. Feb 2012;87(2):217-9. doi:10.1002/ajh.22221
276. Vasavda N, Woodley C, Allman M, et al. Effects of co-existing alpha-thalassaemia in sickle cell disease on hydroxycarbamide therapy and circulating nucleic acids. *Br J Haematol*. Apr 2012;157(2):249-52. doi:10.1111/j.1365-2141.2011.08937.x
277. Pandey S, Pandey S, Mishra RM, Sharma M, Saxena R. Genotypic influence of alpha-deletions on the phenotype of Indian sickle cell anemia patients. *Korean J Hematol*. Sep 2011;46(3):192-5. doi:10.5045/kjh.2011.46.3.192
278. Ngo DA, Aygun B, Akinsheye I, et al. Fetal haemoglobin levels and haematological characteristics of compound heterozygotes for haemoglobin S and deletional hereditary persistence of fetal haemoglobin. *Br J Haematol*. Jan 2012;156(2):259-64. doi:10.1111/j.1365-2141.2011.08916.x
279. Day TG, Drasar ER, Fulford T, Sharpe CC, Thein SL. Association between hemolysis and albuminuria in adults with sickle cell anemia. *Haematologica*. Feb 2012;97(2):201-5. doi:10.3324/haematol.2011.050336
280. Joly P, Pondarre C, Bardel C, Francina A, Martin C. The alpha-globin genotype does not influence sickle cell disease severity in a retrospective cross-validation study of the pediatric severity score. *Eur J Haematol*. Jan 2012;88(1):61-7. doi:10.1111/j.1600-0609.2011.01705.x
281. Ashley-Koch AE, Okocha EC, Garrett ME, et al. MYH9 and APOL1 are both associated with sickle cell disease nephropathy. *Br J Haematol*. Nov 2011;155(3):386-94. doi:10.1111/j.1365-2141.2011.08832.x
282. Darbari DS, Onyekwere O, Nouraie M, et al. Markers of severe vaso-occlusive painful episode frequency in children and adolescents with sickle cell anemia. *J Pediatr*. Feb 2012;160(2):286-90. doi:10.1016/j.jpeds.2011.07.018
283. Ware RE, Despotovic JM, Mortier NA, et al. Pharmacokinetics, pharmacodynamics, and pharmacogenetics of hydroxyurea treatment for children with sickle cell anemia. *Blood*. Nov 3 2011;118(18):4985-91. doi:10.1182/blood-2011-07-364190
284. Cajado C, Cerqueira BA, Couto FD, et al. TNF-alpha and IL-8: serum levels and gene polymorphisms (-308G>A and -251A>T) are associated with classical biomarkers and medical history in children with sickle cell anemia. *Cytokine*. Nov 2011;56(2):312-7. doi:10.1016/j.cyto.2011.07.002
285. Filho IL, Leite AC, Moura PG, et al. Genetic polymorphisms and cerebrovascular disease in children with sickle cell anemia from Rio de Janeiro, Brazil. *Arq Neuropsiquiatr*. Jun 2011;69(3):431-5. doi:10.1590/s0004-282x2011000400004
286. Kumar R, Panigrahi I, Dalal A, Agarwal S. Sickle cell anemia--molecular diagnosis and prenatal counseling: SGPGI experience. *Indian J Pediatr*. Jan 2012;79(1):68-74. doi:10.1007/s12098-011-0510-1
287. Alsultan A, Solovieff N, Aleem A, et al. Fetal hemoglobin in sickle cell anemia: Saudi patients from the Southwestern province have similar HBB haplotypes but higher HbF levels than African Americans. *Am J Hematol*. Jul 2011;86(7):612-4. doi:10.1002/ajh.22032
288. Flanagan JM, Frohlich DM, Howard TA, et al. Genetic predictors for stroke in children with sickle cell anemia. *Blood*. Jun 16 2011;117(24):6681-4. doi:10.1182/blood-2011-01-332205
289. Silva DG, Belini Junior E, Torres Lde S, et al. Relationship between oxidative stress, glutathione S-transferase polymorphisms and hydroxyurea treatment in sickle cell anemia. *Blood Cells Mol Dis*. Jun 15 2011;47(1):23-8. doi:10.1016/j.bcmd.2011.03.004
290. Rusanova I, Cossio G, Moreno B, et al. beta-globin gene cluster haplotypes in sickle cell patients from Panama. *Am J Hum Biol*. May-Jun 2011;23(3):377-80. doi:10.1002/ajhb.21148
291. Bhatnagar P, Purvis S, Barron-Casella E, et al. Genome-wide association study identifies genetic variants influencing F-cell levels in sickle-cell patients. *J Hum Genet*. Apr 2011;56(4):316-23. doi:10.1038/jhg.2011.12
292. Dworkis DA, Klings ES, Solovieff N, et al. Severe sickle cell anemia is associated with increased plasma levels of TNF-R1 and VCAM-1. *Am J Hematol*. Feb 2011;86(2):220-3. doi:10.1002/ajh.21928
293. Mukherjee MB, Nadkarni AH, Gorakshakar AC, Ghosh K, Mohanty D, Colah RB. Clinical, hematologic and molecular variability of sickle cell-beta thalassemia in western India. *Indian J Hum Genet*. Sep 2010;16(3):154-8. doi:10.4103/0971-6866.73410

© 2023 Kirkham JK et al. *JAMA Network Open*.

294. Vicari P, Silva GS, Nogutti MA, et al. Absence of association between TNF-alpha polymorphism and cerebral large-vessel abnormalities in adults with sickle cell anemia. *Acta Haematol*. 2011;125(3):141-4. doi:10.1159/000321935
295. Belisario AR, Rodrigues CV, Martins ML, Silva CM, Viana MB. Coinheritance of alpha-thalassemia decreases the risk of cerebrovascular disease in a cohort of children with sickle cell anemia. *Hemoglobin*. 2010;34(6):516-29. doi:10.3109/03630269.2010.526003
296. Makani J, Menzel S, Nkya S, et al. Genetics of fetal hemoglobin in Tanzanian and British patients with sickle cell anemia. *Blood*. Jan 27 2011;117(4):1390-2. doi:10.1182/blood-2010-08-302703
297. Galarneau G, Palmer CD, Sankaran VG, Orkin SH, Hirschhorn JN, Lettre G. Fine-mapping at three loci known to affect fetal hemoglobin levels explains additional genetic variation. *Nat Genet*. Dec 2010;42(12):1049-51. doi:10.1038/ng.707
298. Belisario AR, Martins ML, Brito AM, Rodrigues CV, Silva CM, Viana MB. beta-globin gene cluster haplotypes in a cohort of 221 children with sickle cell anemia or Sbeta(0)-thalassemia and their association with clinical and hematological features. *Acta Haematol*. 2010;124(3):162-70. doi:10.1159/000320271
299. Adekile AD, Haider MZ. Haptoglobin gene polymorphisms in sickle cell disease patients with different betaS-globin gene haplotypes. *Med Princ Pract*. 2010;19(6):447-50. doi:10.1159/000320302
300. Rusanova I, Escames G, Cossio G, et al. Oxidative stress status, clinical outcome, and beta-globin gene cluster haplotypes in pediatric patients with sickle cell disease. *Eur J Haematol*. Dec 2010;85(6):529-37. doi:10.1111/j.1600-0609.2010.01528.x
301. Italia KY, Jijina FF, Jain D, et al. The effect of UGT1A1 promoter polymorphism on bilirubin response to hydroxyurea therapy in hemoglobinopathies. *Clin Biochem*. Nov 2010;43(16-17):1329-32. doi:10.1016/j.clinbiochem.2010.08.006
302. Mecabo G, Hayashida DY, Azevedo-Shimmoto MM, et al. Duffy-negative is associated with hemolytic phenotype of sickle cell anemia. *Clin Immunol*. Sep 2010;136(3):458-9; author reply 460-1. doi:10.1016/j.clim.2010.06.006
303. Barbosa CG, Goncalves-Santos NJ, Souza-Ribeiro SB, et al. Promoter region sequence differences in the A and G gamma globin genes of Brazilian sickle cell anemia patients. *Braz J Med Biol Res*. Aug 2010;43(8):705-11. doi:10.1590/s0100-879x2010007500062
304. Nebor D, Broquere C, Brudey K, et al. Alpha-thalassemia is associated with a decreased occurrence and a delayed age-at-onset of albuminuria in sickle cell anemia patients. *Blood Cells Mol Dis*. Aug 15 2010;45(2):154-8. doi:10.1016/j.bcmd.2010.06.003
305. Nouraie M, Reading NS, Campbell A, et al. Association of G6PD with lower haemoglobin concentration but not increased haemolysis in patients with sickle cell anaemia. *Br J Haematol*. Jul 2010;150(2):218-25. doi:10.1111/j.1365-2141.2010.08215.x
306. Joannes MO, Loko G, Deloumeaux J, Chout R, Marianne-Pepin T. Association of the +874 T/A interferon gamma polymorphism with infections in sickle cell disease. *Int J Immunogenet*. Aug 2010;37(4):219-23. doi:10.1111/j.1744-313X.2010.00912.x
307. Farra C, Zahed L, Nietert PJ, et al. Vascular at-risk genotypes and disease severity in Lebanese sickle cell disease patients. *Am J Hematol*. May 2010;85(5):395-6. doi:10.1002/ajh.21688
308. Nebor D, Durpes MC, Mougenel D, et al. Association between Duffy antigen receptor for chemokines expression and levels of inflammation markers in sickle cell anemia patients. *Clin Immunol*. Jul 2010;136(1):116-22. doi:10.1016/j.clim.2010.02.023
309. Al-Saqladi AW, Brabin BJ, Bin-Gadeem HA, Kanhai WA, Phylipsen M, Harteveld CL. Beta-globin gene cluster haplotypes in Yemeni children with sickle cell disease. *Acta Haematol*. 2010;123(3):182-5. doi:10.1159/000294965
310. Haymann JP, Stankovic K, Levy P, et al. Glomerular hyperfiltration in adult sickle cell anemia: a frequent hemolysis associated feature. *Clin J Am Soc Nephrol*. May 2010;5(5):756-61. doi:10.2215/CJN.08511109
311. Mendonca TF, Oliveira MC, Vasconcelos LR, et al. Association of variant alleles of MBL2 gene with vasoocclusive crisis in children with sickle cell anemia. *Blood Cells Mol Dis*. Apr 15 2010;44(4):224-8. doi:10.1016/j.bcmd.2010.02.004
312. Al-Saqladi AW, Harper G, Delpisheh A, Fijnvandraat K, Bin-Gadeem HA, Brabin BJ. Frequency of the MTHFR C677T polymorphism in Yemeni children with sickle cell disease. *Hemoglobin*. 2010;34(1):67-77. doi:10.3109/09687630903554111

313. Sebastiani P, Solovieff N, Hartley SW, et al. Genetic modifiers of the severity of sickle cell anemia identified through a genome-wide association study. *Am J Hematol*. Jan 2010;85(1):29-35. doi:10.1002/ajh.21572
314. Solovieff N, Milton JN, Hartley SW, et al. Fetal hemoglobin in sickle cell anemia: genome-wide association studies suggest a regulatory region in the 5' olfactory receptor gene cluster. *Blood*. Mar 4 2010;115(9):1815-22. doi:10.1182/blood-2009-08-239517
315. Cordero EA, Veit TD, da Silva MA, Jacques SM, Silla LM, Chies JA. HLA-G polymorphism influences the susceptibility to HCV infection in sickle cell disease patients. *Tissue Antigens*. Oct 2009;74(4):308-13. doi:10.1111/j.1399-0039.2009.01331.x
316. Al-Subaie AM, Fawaz NA, Mahdi N, et al. Human platelet alloantigens (HPA) 1, HPA2, HPA3, HPA4, and HPA5 polymorphisms in sickle cell anemia patients with vaso-occlusive crisis. *Eur J Haematol*. Dec 1 2009;83(6):579-85. doi:10.1111/j.1600-0609.2009.01339.x
317. Hoppe C, Klitz W, Vichinsky E, Styles L. HLA type and risk of alloimmunization in sickle cell disease. *Am J Hematol*. Jul 2009;84(7):462-4. doi:10.1002/ajh.21442
318. Oliveira MC, Mendonca TF, Vasconcelos LR, et al. Association of the MBL2 gene EXON1 polymorphism and vasoocclusive crisis in patients with sickle cell anemia. *Acta Haematol*. 2009;121(4):212-5. doi:10.1159/000220335
319. Mahdi N, Al-Subaie AM, Al-Ola K, et al. HLA DRB1*130101-DQB1*060101 haplotype is associated with acute chest syndrome in sickle cell anemia patients. *Tissue Antigens*. Mar 2009;73(3):245-9. doi:10.1111/j.1399-0039.2008.01189.x
320. Tatari-Calderone Z, Minniti CP, Kratovil T, et al. rs660 polymorphism in Ro52 (SSA1; TRIM21) is a marker for age-dependent tolerance induction and efficiency of alloimmunization in sickle cell disease. *Mol Immunol*. Nov 2009;47(1):64-70. doi:10.1016/j.molimm.2008.12.027
321. Creary LE, Ulug P, Menzel S, et al. Genetic variation on chromosome 6 influences F cell levels in healthy individuals of African descent and HbF levels in sickle cell patients. *PLoS One*. 2009;4(1):e4218. doi:10.1371/journal.pone.0004218
322. Ulug P, Vasavda N, Awogbade M, Cunningham J, Menzel S, Thein SL. Association of sickle avascular necrosis with bone morphogenic protein 6. *Ann Hematol*. Aug 2009;88(8):803-5. doi:10.1007/s00277-008-0659-5
323. Bernaudin F, Verlhac S, Chevret S, et al. G6PD deficiency, absence of alpha-thalassemia, and hemolytic rate at baseline are significant independent risk factors for abnormally high cerebral velocities in patients with sickle cell anemia. *Blood*. Nov 15 2008;112(10):4314-7. doi:10.1182/blood-2008-03-143891
324. Carpenter SL, Lieff S, Howard TA, Eggleston B, Ware RE. UGT1A1 promoter polymorphisms and the development of hyperbilirubinemia and gallbladder disease in children with sickle cell anemia. *Am J Hematol*. Oct 2008;83(10):800-3. doi:10.1002/ajh.21264
325. Sedgewick AE, Timofeev N, Sebastiani P, et al. BCL11A is a major HbF quantitative trait locus in three different populations with beta-hemoglobinopathies. *Blood Cells Mol Dis*. Nov-Dec 2008;41(3):255-258. doi:10.1016/j.bcmd.2008.06.007
326. Lettre G, Sankaran VG, Bezerra MA, et al. DNA polymorphisms at the BCL11A, HBS1L-MYB, and beta-globin loci associate with fetal hemoglobin levels and pain crises in sickle cell disease. *Proc Natl Acad Sci U S A*. Aug 19 2008;105(33):11869-74. doi:10.1073/pnas.0804799105
327. Ugochukwu CC, Okpala I, Pantelidis P, Inusa B, Ibegbulam O, Onyekwere O. l-selectin gene polymorphisms and complications of sickle cell disease. *Int J Lab Hematol*. Aug 2008;30(4):312-6. doi:10.1111/j.1751-553X.2007.00961.x
328. Ganesh A, Al-Zuhaibi S, Pathare A, et al. Orbital infarction in sickle cell disease. *Am J Ophthalmol*. Oct 2008;146(4):595-601. doi:10.1016/j.ajo.2008.05.041
329. Taylor JGt, Nolan VG, Mendelsohn L, Kato GJ, Gladwin MT, Steinberg MH. Chronic hyper-hemolysis in sickle cell anemia: association of vascular complications and mortality with less frequent vasoocclusive pain. *PLoS One*. May 7 2008;3(5):e2095. doi:10.1371/journal.pone.0002095
330. Martins R, Morais A, Dias A, et al. Early modification of sickle cell disease clinical course by UDP-glucuronosyltransferase 1A1 gene promoter polymorphism. *J Hum Genet*. 2008;53(6):524-528. doi:10.1007/s10038-008-0281-3
331. Eyler CE, Jackson T, Elliott LE, et al. beta(2)-Adrenergic receptor and adenylate cyclase gene polymorphisms affect sickle red cell adhesion. *Br J Haematol*. Apr 2008;141(1):105-8. doi:10.1111/j.1365-2141.2008.07008.x

© 2023 Kirkham JK et al. *JAMA Network Open*.

332. Kumkhaek C, Taylor JGt, Zhu J, Hoppe C, Kato GJ, Rodgers GP. Fetal haemoglobin response to hydroxycarbamide treatment and sar1a promoter polymorphisms in sickle cell anaemia. *Br J Haematol*. Apr 2008;141(2):254-9. doi:10.1111/j.1365-2141.2008.07045.x
333. Al-Ola K, Mahdi N, Al-Subaie AM, Ali ME, Al-Absi IK, Almawi WY. Evidence for HLA class II susceptible and protective haplotypes for osteomyelitis in pediatric patients with sickle cell anemia. *Tissue Antigens*. May 2008;71(5):453-7. doi:10.1111/j.1399-0039.2008.01012.x
334. Mahdi N, Al-Ola K, Al-Subaie AM, et al. HLA class II haplotypes distinctly associated with vaso-occlusion in children with sickle cell disease. *Clin Vaccine Immunol*. Apr 2008;15(4):729-31. doi:10.1128/CVI.00425-07
335. Afenyi-Annan A, Kail M, Combs MR, Orringer EP, Ashley-Koch A, Telen MJ. Lack of Duffy antigen expression is associated with organ damage in patients with sickle cell disease. *Transfusion*. May 2008;48(5):917-24. doi:10.1111/j.1537-2995.2007.01622.x
336. Uda M, Galanello R, Sanna S, et al. Genome-wide association study shows BCL11A associated with persistent fetal hemoglobin and amelioration of the phenotype of beta-thalassemia. *Proc Natl Acad Sci U S A*. Feb 5 2008;105(5):1620-5. doi:10.1073/pnas.0711566105
337. Sebastiani P, Zhao Z, Abad-Grau MM, et al. A hierarchical and modular approach to the discovery of robust associations in genome-wide association studies from pooled DNA samples. *BMC Genet*. Jan 14 2008;9:6. doi:10.1186/1471-2156-9-6
338. Ashley-Koch AE, Elliott L, Kail ME, et al. Identification of genetic polymorphisms associated with risk for pulmonary hypertension in sickle cell disease. *Blood*. Jun 15 2008;111(12):5721-6. doi:10.1182/blood-2007-02-074849
339. Mourad H, Fadel W, El Batch M, Rowisha M. Heamostatic and genetic predisposing factors for stroke in children with sickle cell anemia. *Egypt J Immunol*. 2008;15(1):25-37.
340. Bagdasaryan R, Glasser L, Quillen K, Chaves F, Xu D. Effect of hydroxyurea on immature reticulocyte fraction in sickle cell anemia. *Lab Hematol*. 2007;13(3):93-7. doi:10.1532/LH96.07008
341. Tamouza R, Busson M, Fortier C, et al. HLA-E*0101 allele in homozygous state favors severe bacterial infections in sickle cell anemia. *Hum Immunol*. Oct 2007;68(10):849-53. doi:10.1016/j.humimm.2007.08.260
342. Sebastiani P, Wang L, Nolan VG, et al. Fetal hemoglobin in sickle cell anemia: Bayesian modeling of genetic associations. *Am J Hematol*. Mar 2008;83(3):189-95. doi:10.1002/ajh.21048
343. Taylor JGt, Ackah D, Cobb C, et al. Mutations and polymorphisms in hemoglobin genes and the risk of pulmonary hypertension and death in sickle cell disease. *Am J Hematol*. Jan 2008;83(1):6-14. doi:10.1002/ajh.21035
344. Hoppe C, Klitz W, D'Harlingue K, et al. Confirmation of an association between the TNF(-308) promoter polymorphism and stroke risk in children with sickle cell anemia. *Stroke*. Aug 2007;38(8):2241-6. doi:10.1161/STROKEAHA.107.483115
345. Vasavda N, Menzel S, Kondaveeti S, et al. The linear effects of alpha-thalassaemia, the UGT1A1 and HMOX1 polymorphisms on cholelithiasis in sickle cell disease. *Br J Haematol*. Jul 2007;138(2):263-70. doi:10.1111/j.1365-2141.2007.06643.x
346. Adekile A, Al-Kandari M, Haider M, Rajaa M, D'Souza M, Sukumaran J. Hemoglobin F concentration as a function of age in Kuwaiti sickle cell disease patients. *Med Princ Pract*. 2007;16(4):286-90. doi:10.1159/000102151
347. Elliott L, Ashley-Koch AE, De Castro L, et al. Genetic polymorphisms associated with priapism in sickle cell disease. *Br J Haematol*. May 2007;137(3):262-7. doi:10.1111/j.1365-2141.2007.06560.x
348. Duckworth L, Hsu L, Feng H, et al. Physician-diagnosed asthma and acute chest syndrome: associations with NOS polymorphisms. *Pediatr Pulmonol*. Apr 2007;42(4):332-8. doi:10.1002/ppul.20582
349. Nolan VG, Ma Q, Cohen HT, et al. Estimated glomerular filtration rate in sickle cell anemia is associated with polymorphisms of bone morphogenetic protein receptor 1B. *Am J Hematol*. Mar 2007;82(3):179-84. doi:10.1002/ajh.20800
350. Chaar V, Tarer V, Etienne-Julan M, Diara JP, Elion J, Romana M. ET-1 and ecNOS gene polymorphisms andsusceptibility to acute chest syndrome and painful vaso-occlusive crises in children with sickle cell anemia. *Haematologica*. Sep 2006;91(9):1277-8.
351. Moreira Neto F, Lourenco DM, Noguti MA, et al. The clinical impact of MTHFR polymorphism on the vascular complications of sickle cell disease. *Braz J Med Biol Res*. Oct 2006;39(10):1291-5. doi:10.1590/s0100-879x2006001000004

© 2023 Kirkham JK et al. *JAMA Network Open*.

352. Adewoye AH, Nolan VG, Ma Q, et al. Association of polymorphisms of IGF1R and genes in the transforming growth factor- beta /bone morphogenetic protein pathway with bacteremia in sickle cell anemia. *Clin Infect Dis*. Sep 1 2006;43(5):593-8. doi:10.1086/506356
353. Nolan VG, Adewoye A, Baldwin C, et al. Sickle cell leg ulcers: associations with haemolysis and SNPs in Klotho, TEK and genes of the TGF-beta/BMP pathway. *Br J Haematol*. Jun 2006;133(5):570-8. doi:10.1111/j.1365-2141.2006.06074.x
354. Lima CS, Rocha EM, Silva NM, Sonatti MF, Costa FF, Saad ST. Risk factors for conjunctival and retinal vessel alterations in sickle cell disease. *Acta Ophthalmol Scand*. Apr 2006;84(2):234-41. doi:10.1111/j.1600-0420.2005.00604.x
355. Chaar V, Keclard L, Etienne-Julan M, et al. UGT1A1 polymorphism outweighs the modest effect of deletional (-3.7 kb) alpha-thalassemia on cholelithogenesis in sickle cell anemia. *Am J Hematol*. May 2006;81(5):377-9. doi:10.1002/ajh.20574
356. Tarer V, Etienne-Julan M, Diara JP, et al. Sickle cell anemia in Guadeloupean children: pattern and prevalence of acute clinical events. *Eur J Haematol*. Mar 2006;76(3):193-9. doi:10.1111/j.1600-0609.2005.00590.x
357. Vargas AE, da Silva MA, Silla L, Chies JA. Polymorphisms of chemokine receptors and eNOS in Brazilian patients with sickle cell disease. *Tissue Antigens*. Dec 2005;66(6):683-90. doi:10.1111/j.1399-0039.2005.00506.x
358. Adekile A, Kutlar F, McKie K, et al. The influence of uridine diphosphate glucuronosyl transferase 1A promoter polymorphisms, beta-globin gene haplotype, co-inherited alpha-thalassemia trait and Hb F on steady-state serum bilirubin levels in sickle cell anemia. *Eur J Haematol*. Aug 2005;75(2):150-5. doi:10.1111/j.1600-0609.2005.00477.x
359. Costa RN, Conran N, Albuquerque DM, Soares PH, Saad ST, Costa FF. Association of the G-463A myeloperoxidase polymorphism with infection in sickle cell anemia. *Haematologica*. Jul 2005;90(7):977-9.
360. Adekile A, Haider MZ, Marouf R, Adekile AD. HLA-DRB1 alleles in Hb SS patients with avascular necrosis of the femoral head. *Am J Hematol*. May 2005;79(1):8-10. doi:10.1002/ajh.20311
361. Baldwin C, Nolan VG, Wyszynski DF, et al. Association of klotho, bone morphogenic protein 6, and annexin A2 polymorphisms with sickle cell osteonecrosis. *Blood*. Jul 1 2005;106(1):372-5. doi:10.1182/blood-2005-02-0548
362. Sebastiani P, Ramoni MF, Nolan V, Baldwin CT, Steinberg MH. Genetic dissection and prognostic modeling of overt stroke in sickle cell anemia. *Nat Genet*. Apr 2005;37(4):435-40. doi:10.1038/ng1533
363. Chaar V, Keclard L, Diara JP, et al. Association of UGT1A1 polymorphism with prevalence and age at onset of cholelithiasis in sickle cell anemia. *Haematologica*. Feb 2005;90(2):188-99.
364. Nolan VG, Baldwin C, Ma Q, et al. Association of single nucleotide polymorphisms in klotho with priapism in sickle cell anaemia. *Br J Haematol*. Jan 2005;128(2):266-72. doi:10.1111/j.1365-2141.2004.05295.x
365. Adorno EV, Zanette A, Lyra I, et al. The beta-globin gene cluster haplotypes in sickle cell anemia patients from Northeast Brazil: a clinical and molecular view. *Hemoglobin*. Aug 2004;28(3):267-71. doi:10.1081/hem-120040310
366. Bakanay SM, Dainer E, Clair B, et al. Mortality in sickle cell patients on hydroxyurea therapy. *Blood*. Jan 15 2005;105(2):545-7. doi:10.1182/blood-2004-01-0322
367. Haverfield EV, McKenzie CA, Forrester T, et al. UGT1A1 variation and gallstone formation in sickle cell disease. *Blood*. Feb 1 2005;105(3):968-72. doi:10.1182/blood-2004-02-0521
368. Castro V, Alberto FL, Costa RN, et al. Polymorphism of the human platelet antigen-5 system is a risk factor for occlusive vascular complications in patients with sickle cell anemia. *Vox Sang*. Aug 2004;87(2):118-23. doi:10.1111/j.1423-0410.2004.00536.x
369. Romana M, Diara JP, Doumbo L, et al. Angiotensinogen gene associated polymorphisms and risk of stroke in sickle cell anemia: Additional data supporting an association. *Am J Hematol*. Jul 2004;76(3):310-1. doi:10.1002/ajh.20078
370. Ofori-Acquah SF, Lalloz MR, Serjeant G, Layton DM. Dominant influence of gamma-globin promoter polymorphisms on fetal haemoglobin expression in sickle cell disease. *Cell Mol Biol (Noisy-le-grand)*. Feb 2004;50(1):35-42.
371. Wyszynski DF, Baldwin CT, Cleves MA, et al. Polymorphisms near a chromosome 6q QTL area are associated with modulation of fetal hemoglobin levels in sickle cell anemia. *Cell Mol Biol (Noisy-le-grand)*. Feb 2004;50(1):23-33.

© 2023 Kirkham JK et al. *JAMA Network Open*.

372. Sharan K, Surrey S, Ballas S, et al. Association of T-786C eNOS gene polymorphism with increased susceptibility to acute chest syndrome in females with sickle cell disease. *Br J Haematol*. Jan 2004;124(2):240-3. doi:10.1046/j.1365-2141.2003.04762.x
373. Jeng MR, Adams-Graves P, Howard TA, Whorton MR, Li CS, Ware RE. Identification of hemochromatosis gene polymorphisms in chronically transfused patients with sickle cell disease. *Am J Hematol*. Dec 2003;74(4):243-8. doi:10.1002/ajh.10426
374. Hoppe C, Klitz W, Cheng S, et al. Gene interactions and stroke risk in children with sickle cell anemia. *Blood*. Mar 15 2004;103(6):2391-6. doi:10.1182/blood-2003-09-3015
375. Goncalves MS, Bomfim GC, Maciel E, et al. BetaS-haplotypes in sickle cell anemia patients from Salvador, Bahia, Northeastern Brazil. *Braz J Med Biol Res*. Oct 2003;36(10):1283-8. doi:10.1590/s0100-879x2003001000001
376. Hsu LL, Miller ST, Wright E, et al. Alpha Thalassemia is associated with decreased risk of abnormal transcranial Doppler ultrasonography in children with sickle cell anemia. *J Pediatr Hematol Oncol*. Aug 2003;25(8):622-8. doi:10.1097/00043426-200308000-00007
377. Fertrin KY, Melo MB, Assis AM, Saad ST, Costa FF. UDP-glucuronosyltransferase 1 gene promoter polymorphism is associated with increased serum bilirubin levels and cholecystectomy in patients with sickle cell anemia. *Clin Genet*. Aug 2003;64(2):160-2. doi:10.1034/j.1399-0004.2003.00113.x
378. Inati A, Taher A, Bou Alawi W, et al. Beta-globin gene cluster haplotypes and HbF levels are not the only modulators of sickle cell disease in Lebanon. *Eur J Haematol*. Feb 2003;70(2):79-83. doi:10.1034/j.1600-0609.2003.00016.x
379. Taylor JGt, Tang DC, Savage SA, et al. Variants in the VCAM1 gene and risk for symptomatic stroke in sickle cell disease. *Blood*. Dec 15 2002;100(13):4303-9. doi:10.1182/blood-2001-12-0306
380. Wali YA, Al-Lamki Z, Hussein SS, et al. Splenic function in Omani children with sickle cell disease: correlation with severity index, hemoglobin phenotype, iron status, and alpha-thalassemia trait. *Pediatr Hematol Oncol*. Oct-Nov 2002;19(7):491-500. doi:10.1080/08880010290097314
381. Powars DR, Hiti A, Ramicone E, Johnson C, Chan L. Outcome in hemoglobin SC disease: a four-decade observational study of clinical, hematologic, and genetic factors. *Am J Hematol*. Jul 2002;70(3):206-15. doi:10.1002/ajh.10140
382. el-Hazmi MA, Warsy AS. Pattern for alpha-thalassaemia in Yemeni sickle-cell-disease patients. *East Mediterr Health J*. Nov 1999;5(6):1159-64.
383. Passon RG, Howard TA, Zimmerman SA, Schultz WH, Ware RE. Influence of bilirubin uridine diphosphate-glucuronosyltransferase 1A promoter polymorphisms on serum bilirubin levels and cholelithiasis in children with sickle cell anemia. *J Pediatr Hematol Oncol*. Oct 2001;23(7):448-51. doi:10.1097/00043426-200110000-00011
384. Tamouza R, Neonato MG, Busson M, et al. Infectious complications in sickle cell disease are influenced by HLA class II alleles. *Hum Immunol*. Mar 2002;63(3):194-9. doi:10.1016/s0198-8859(01)00378-0
385. Romana M, Muralitharan S, Ramasawmy R, Nagel RL, Krishnamoorthy R. Thrombosis-associated gene variants in sickle cell anemia. *Thromb Haemost*. Feb 2002;87(2):356-8.
386. Taylor JG, Tang D, Foster CB, Serjeant GR, Rodgers GP, Chanock SJ. Patterns of low-affinity immunoglobulin receptor polymorphisms in stroke and homozygous sickle cell disease. *Am J Hematol*. Feb 2002;69(2):109-14. doi:10.1002/ajh.10048
387. Tang DC, Prauner R, Liu W, et al. Polymorphisms within the angiotensinogen gene (GT-repeat) and the risk of stroke in pediatric patients with sickle cell disease: a case-control study. *Am J Hematol*. Nov 2001;68(3):164-9. doi:10.1002/ajh.1173
388. Luporini SM, Bendit I, Manhani R, Bracco OL, Manzella L, Giannella-Neto D. Growth hormone and insulin-like growth factor I axis and growth of children with different sickle cell anemia haplotypes. *J Pediatr Hematol Oncol*. Aug-Sep 2001;23(6):357-63. doi:10.1097/00043426-200108000-00007
389. Kutlar A, Kutlar F, Turker I, Tural C. The methylene tetrahydrofolate reductase (C677T) mutation as a potential risk factor for avascular necrosis in sickle cell disease. *Hemoglobin*. May 2001;25(2):213-7. doi:10.1081/hem-100104029
390. Olatunji PO, Davies SC. The predictive value of white cell count in assessing clinical severity of sickle cell anaemia in Afro-Caribbeans patients. *Afr J Med Med Sci*. Mar 2000;29(1):27-30.
391. Adekile AD, Gupta R, Yacoub F, Sinan T, Al-Bloushi M, Haider MZ. Avascular necrosis of the hip in children with sickle cell disease and high Hb F: magnetic resonance imaging findings and influence of alpha-thalassemia trait. *Acta Haematol*. 2001;105(1):27-31. doi:10.1159/000046529

392. Adekile AD, Kutlar F, Haider MZ, Kutlar A. Frequency of the 677 C-->T mutation of the methylenetetrahydrofolate reductase gene among Kuwaiti sickle cell disease patients. *Am J Hematol*. Apr 2001;66(4):263-6. doi:10.1002/ajh.1055
393. Ballas SK, Marcolina MJ. Determinants of red cell survival and erythropoietic activity in patients with sickle cell anemia in the steady state. *Hemoglobin*. Nov 2000;24(4):277-86. doi:10.3109/03630260008993134
394. Raghupathy R, Haider MZ, Azizieh F, Abdelsalam R, D'Souza TM, Adekile AD. Th1 and Th2 cytokine profiles in sickle cell disease. *Acta Haematol*. 2000;103(4):197-202. doi:10.1159/000041049
395. Neonato MG, Guilloud-Bataille M, Beauvais P, et al. Acute clinical events in 299 homozygous sickle cell patients living in France. French Study Group on Sickle Cell Disease. *Eur J Haematol*. Sep 2000;65(3):155-64. doi:10.1034/j.1600-0609.2000.90210.x
396. Styles LA, Hoppe C, Klitz W, Vichinsky E, Lubin B, Trachtenberg E. Evidence for HLA-related susceptibility for stroke in children with sickle cell disease. *Blood*. Jun 1 2000;95(11):3562-7.
397. Mouele R, Pambou O, Feingold J, Galacteros F. alpha-thalassemia in Bantu population from Congo-Brazzaville: its interaction with sickle cell anemia. *Hum Hered*. Mar-Apr 2000;50(2):118-25. doi:10.1159/000022899
398. el-Hazmi MA, Warsy AS. Alpha thalassaemia in Yemeni children with sickle cell disease. *J Trop Pediatr*. Dec 1999;45(6):370-4. doi:10.1093/tropej/45.6.370
399. Driscoll MC, Prauner R. The methylenetetrahydrofolate reductase gene C677T mutant and ischemic stroke in sickle cell disease. *Thromb Haemost*. Dec 1999;82(6):1780-1.
400. Cumming AM, Olujohungbe A, Keeney S, Singh H, Hay CR, Serjeant GR. The methylenetetrahydrofolate reductase gene C677T polymorphism in patients with homozygous sickle cell disease and stroke. *Br J Haematol*. Dec 1999;107(3):569-71. doi:10.1046/j.1365-2141.1999.01728.x
401. Balasa VV, Gruppo RA, Gartside PS, Kalinyak KA. Correlation of the C677T MTHFR genotype with homocysteine levels in children with sickle cell disease. *J Pediatr Hematol Oncol*. Sep-Oct 1999;21(5):397-400. doi:10.1097/00043426-199909000-00011
402. Neonato MG, Lu CY, Guilloud-Bataille M, et al. Genetic polymorphism of the mannose-binding protein gene in children with sickle cell disease: identification of three new variant alleles and relationship to infections. *Eur J Hum Genet*. Sep 1999;7(6):679-86. doi:10.1038/sj.ejhg.5200360
403. Mouele R, Boukila V, Fourcade V, Feingold J, Galacteros F. Sickle-cell disease in Brazzaville, Congo: genetical, hematological, biochemical and clinical aspects. *Acta Haematol*. 1999;101(4):178-84. doi:10.1159/000040950
404. Guasch A, Zayas CF, Eckman JR, Muralidharan K, Zhang W, Elsas LJ. Evidence that microdeletions in the alpha globin gene protect against the development of sickle cell glomerulopathy in humans. *J Am Soc Nephrol*. May 1999;10(5):1014-9. doi:10.1681/ASN.V1051014
405. Kinney TR, Sleeper LA, Wang WC, et al. Silent cerebral infarcts in sickle cell anemia: a risk factor analysis. The Cooperative Study of Sickle Cell Disease. *Pediatrics*. Mar 1999;103(3):640-5. doi:10.1542/peds.103.3.640
406. Ofori-Acquah SF, Lalloz MR, Layton DM. Localisation of cis regulatory elements at the beta-globin locus: analysis of hybrid haplotype chromosomes. *Biochem Biophys Res Commun*. Jan 8 1999;254(1):181-7. doi:10.1006/bbrc.1998.9901
407. Haider MZ, Ashebu S, Aduh P, Adekile AD. Influence of alpha-thalassemia on cholelithiasis in SS patients with elevated Hb F. *Acta Haematol*. Dec 1998;100(3):147-50. doi:10.1159/000040890
408. Zimmerman SA, Ware RE. Inherited DNA mutations contributing to thrombotic complications in patients with sickle cell disease. *Am J Hematol*. Dec 1998;59(4):267-72. doi:10.1002/(sici)1096-8652(199812)59:4<267::aid-ajh1>3.0.co;2-w
409. Steinberg MH, Lu ZH, Nagel RL, et al. Hematological effects of atypical and Cameroon beta-globin gene haplotypes in adult sickle cell anemia. *Am J Hematol*. Oct 1998;59(2):121-6. doi:10.1002/(sici)1096-8652(199810)59:2<121::aid-ajh4>3.0.co;2-#
410. Lee K, Prehu C, Merault G, et al. Genetic and hematological studies in a group of 114 adult patients with SC sickle cell disease. *Am J Hematol*. Sep 1998;59(1):15-21. doi:10.1002/(sici)1096-8652(199809)59:1<15::aid-ajh4>3.0.co;2-2
411. Mukherjee MB, Surve R, Tamankar A, et al. The influence of alpha-thalassaemia on the haematological & clinical expression of sickle cell disease in western India. *Indian J Med Res*. Apr 1998;107:178-81.

© 2023 Kirkham JK et al. *JAMA Network Open*.

412. Chang YP, Maier-Redelsperger M, Smith KD, et al. The relative importance of the X-linked FCP locus and beta-globin haplotypes in determining haemoglobin F levels: a study of SS patients homozygous for beta S haplotypes. *Br J Haematol*. Mar 1997;96(4):806-14. doi:10.1046/j.1365-2141.1997.d01-2094.x
413. Thomas PW, Higgs DR, Serjeant GR. Benign clinical course in homozygous sickle cell disease: a search for predictors. *J Clin Epidemiol*. Feb 1997;50(2):121-6. doi:10.1016/s0895-4356(96)00320-4
414. Steinberg MH, Lu ZH, Barton FB, Terrin ML, Charache S, Dover GJ. Fetal hemoglobin in sickle cell anemia: determinants of response to hydroxyurea. Multicenter Study of Hydroxyurea. *Blood*. Feb 1 1997;89(3):1078-88.
415. Mukherjee MB, Colah RB, Ghosh K, Mohanty D, Krishnamoorthy R. Milder clinical course of sickle cell disease in patients with alpha thalassemia in the Indian subcontinent. *Blood*. Jan 15 1997;89(2):732.
416. Walker TM, Beardsall K, Thomas PW, Serjeant GR. Renal length in sickle cell disease: observations from a cohort study. *Clin Nephrol*. Dec 1996;46(6):384-8.
417. Figueirido MS, Steinberg MH. 5' hypersensitive site-2 and fetal hemoglobin in Brazilians. *Hemoglobin*. Nov 1996;20(4):435-8. doi:10.3109/03630269609005847
418. Figueiredo MS, Kerbauy J, Goncalves MS, et al. Effect of alpha-thalassemia and beta-globin gene cluster haplotypes on the hematological and clinical features of sickle-cell anemia in Brazil. *Am J Hematol*. Oct 1996;53(2):72-6. doi:10.1002/(SICI)1096-8652(199610)53:2<72::AID-AJH3>3.0.CO;2-0
419. Adekile AD, Tuli M, Haider MZ, Al-Zaabi K, Mohannadi S, Owunwanne A. Influence of alpha-thalassemia trait on spleen function in sickle cell anemia patients with high HbF. *Am J Hematol*. Sep 1996;53(1):1-5. doi:10.1002/(SICI)1096-8652(199609)53:1<1::AID-AJH1>3.0.CO;2-V
420. Steinberg MH, Nagel RL, Lawrence C, et al. Beta-globin gene haplotype in Hb SC disease. *Am J Hematol*. Jul 1996;52(3):189-91. doi:10.1002/(SICI)1096-8652(199607)52:3<189::AID-AJH9>3.0.CO;2-P
421. Braden DS, Covitz W, Milner PF. Cardiovascular function during rest and exercise in patients with sickle-cell anemia and coexisting alpha thalassemia-2. *Am J Hematol*. Jun 1996;52(2):96-102. doi:10.1002/(SICI)1096-8652(199606)52:2<96::AID-AJH5>3.0.CO;2-0
422. Keclard L, Ollendorf V, Berchel C, Loret H, Merault G. beta S haplotypes, alpha-globin gene status, and hematological data of sickle cell disease patients in Guadeloupe (F.W.I.). *Hemoglobin*. Feb 1996;20(1):63-74. doi:10.3109/03630269609027911
423. Serjeant G, Serjeant B, Stephens A, et al. Determinants of haemoglobin level in steady-state homozygous sickle cell disease. *Br J Haematol*. Jan 1996;92(1):143-9. doi:10.1046/j.1365-2141.1996.284816.x
424. Gill FM, Sleeper LA, Weiner SJ, et al. Clinical events in the first decade in a cohort of infants with sickle cell disease. Cooperative Study of Sickle Cell Disease. *Blood*. Jul 15 1995;86(2):776-83.
425. al-Hazzaa S, Bird AC, Kulozik A, et al. Ocular findings in Saudi Arabian patients with sickle cell disease. *Br J Ophthalmol*. May 1995;79(5):457-61. doi:10.1136/bjo.79.5.457
426. Steinberg MH, Hsu H, Nagel RL, et al. Gender and haplotype effects upon hematological manifestations of adult sickle cell anemia. *Am J Hematol*. Mar 1995;48(3):175-81. doi:10.1002/ajh.2830480307
427. Chang YC, Smith KD, Moore RD, Serjeant GR, Dover GJ. An analysis of fetal hemoglobin variation in sickle cell disease: the relative contributions of the X-linked factor, beta-globin haplotypes, alpha-globin gene number, gender, and age. *Blood*. Feb 15 1995;85(4):1111-7.
428. Padmos A, Roberts G, Lindahl S, et al. Avascular necrosis of the femoral head in Saudi Arabians with homozygous sickle cell disease - risk factors. *Ann Saudi Med*. Jan 1995;15(1):21-4. doi:10.5144/0256-4947.1995.21
429. Brown AK, Sleeper LA, Miller ST, Pegelow CH, Gill FM, Waclawiw MA. Reference values and hematologic changes from birth to 5 years in patients with sickle cell disease. Cooperative Study of Sickle Cell Disease. *Arch Pediatr Adolesc Med*. Aug 1994;148(8):796-804. doi:10.1001/archpedi.1994.02170080026005
430. Castro O, Brambilla DJ, Thorington B, et al. The acute chest syndrome in sickle cell disease: incidence and risk factors. The Cooperative Study of Sickle Cell Disease. *Blood*. Jul 15 1994;84(2):643-9.

© 2023 Kirkham JK et al. *JAMA Network Open*.

431. el-Hazmi MA, Warsy AS, al-Swailem AR, al-Faleh FZ, al-Jabbar FA. Genetic compounds--Hb S, thalassaemias and enzymopathies: spectrum of interactions. *J Trop Pediatr*. Jun 1994;40(3):149-56. doi:10.1093/tropej/40.3.149
432. Adams RJ, Kutlar A, McKie V, et al. Alpha thalassemia and stroke risk in sickle cell anemia. *Am J Hematol*. Apr 1994;45(4):279-82. doi:10.1002/ajh.2830450402
433. Falusi AG, Olatunji PO. Effects of alpha thalassaemia and haemoglobin F (HbF) level on the clinical severity of sickle-cell anaemia. *Eur J Haematol*. Jan 1994;52(1):13-5. doi:10.1111/j.1600-0609.1994.tb01278.x
434. de Montalembert M, Maier-Redelsperger M, Girot R, et al. Beta-globin gene cluster haplotype and alpha-thalassemia do not correlate with the acute clinical manifestations of sickle cell disease in children. *Blood*. Oct 15 1993;82(8):2595-6.
435. Green NS, Fabry ME, Kaptue-Noche L, Nagel RL. Senegal haplotype is associated with higher HbF than Benin and Cameroon haplotypes in African children with sickle cell anemia. *Am J Hematol*. Oct 1993;44(2):145-6. doi:10.1002/ajh.2830440214
436. Milner PF, Kraus AP, Sebes JI, et al. Osteonecrosis of the humeral head in sickle cell disease. *Clin Orthop Relat Res*. Apr 1993;(289):136-43.
437. Adekile AD, McKie KM, Adeodu OO, et al. Spleen in sickle cell anemia: comparative studies of Nigerian and U.S. patients. *Am J Hematol*. Mar 1993;42(3):316-21. doi:10.1002/ajh.2830420313
438. Fox PD, Higgs DR, Serjeant GR. Influence of alpha thalassaemia on the retinopathy of homozygous sickle cell disease. *Br J Ophthalmol*. Feb 1993;77(2):89-90. doi:10.1136/bjo.77.2.89
439. el-Hazmi MA. Heterogeneity and variation of clinical and haematological expression of haemoglobin S in Saudi Arabs. *Acta Haematol*. 1992;88(2-3):67-71. doi:10.1159/000204654
440. el-Hazmi MA, Bahakim HM, Warsy AS. DNA polymorphism in the beta-globin gene cluster in Saudi Arabs: relation to severity of sickle cell anaemia. *Acta Haematol*. 1992;88(2-3):61-6. doi:10.1159/000204653
441. Milner PF, Kraus AP, Sebes JI, et al. Sickle cell disease as a cause of osteonecrosis of the femoral head. *N Engl J Med*. Nov 21 1991;325(21):1476-81. doi:10.1056/NEJM199111213252104
442. Powars DR, Elliott-Mills DD, Chan L, et al. Chronic renal failure in sickle cell disease: risk factors, clinical course, and mortality. *Ann Intern Med*. Oct 15 1991;115(8):614-20. doi:10.7326/0003-4819-115-8-614
443. Platt OS, Thorington BD, Brambilla DJ, et al. Pain in sickle cell disease. Rates and risk factors. *N Engl J Med*. Jul 4 1991;325(1):11-6. doi:10.1056/NEJM199107043250103
444. Nagel RL, Erlingsson S, Fabry ME, et al. The Senegal DNA haplotype is associated with the amelioration of anemia in African-American sickle cell anemia patients. *Blood*. Mar 15 1991;77(6):1371-5.
445. Rieder RF, Safaya S, Gillette P, et al. Effect of beta-globin gene cluster haplotype on the hematological and clinical features of sickle cell anemia. *Am J Hematol*. Mar 1991;36(3):184-9. doi:10.1002/ajh.2830360305
446. Ballas SK, Talacki CA, Adachi K, Schwartz E, Surrey S, Rappaport E. The Xmn I site (-158, C----T) 5' to the G gamma gene: correlation with the Senegalese haplotype and G gamma globin expression. *Hemoglobin*. 1991;15(5):393-405. doi:10.3109/03630269108998859
447. el-Hazmi MA. Beta-globin gene haplotypes in the Saudi sickle cell anaemia patients. *Hum Hered*. 1990;40(3):177-86. doi:10.1159/000153927
448. Powars DR, Chan L, Schroeder WA. Beta S-gene-cluster haplotypes in sickle cell anemia: clinical implications. *Am J Pediatr Hematol Oncol*. Fall 1990;12(3):367-74. doi:10.1097/00043426-199023000-00022
449. Koshy M, Entsuah R, Koranda A, et al. Leg ulcers in patients with sickle cell disease. *Blood*. Sep 1989;74(4):1403-8.
450. Ballas SK, Talacki CA, Rao VM, Steiner RM. The prevalence of avascular necrosis in sickle cell anemia: correlation with alpha-thalassemia. *Hemoglobin*. 1989;13(7-8):649-55. doi:10.3109/03630268908998842
451. Tejuca M, Martinez G, Ferreira R, et al. Alpha-thalassemia changes the cell density profile in sickle cell anaemia. *Haematologia (Budap)*. 1989;22(3):175-80.
452. Schroeder WA, Powars DR, Kay LM, et al. Beta-cluster haplotypes, alpha-gene status, and hematological data from SS, SC, and S-beta-thalassemia patients in southern California. *Hemoglobin*. 1989;13(4):325-53. doi:10.3109/03630268909003397

© 2023 Kirkham JK et al. *JAMA Network Open*.

453. Ballas SK, Larner J, Smith ED, Surrey S, Schwartz E, Rappaport EF. Rheologic predictors of the severity of the painful sickle cell crisis. *Blood*. Oct 1988;72(4):1216-23.
454. Kulozik AE, Kar BC, Serjeant GR, Serjeant BE, Weatherall DJ. The molecular basis of alpha thalassemia in India. Its interaction with the sickle cell gene. *Blood*. Feb 1988;71(2):467-72.
455. Pajot N, Maier-Redelsperger M, Dode C, Labie D, Girot R. Density distribution of red cells and prognostic significance in 50 patients with homozygous sickle-cell disease. *Haematologia (Budap)*. 1988;21(4):189-97.
456. Felice AE, McKie KM, Cleek MP, Marino EM, Kutlar A, McKie VC. Effects of alpha-thalassemia-2 on the developmental changes of hematological values in children with sickle cell disease from Georgia. *Am J Hematol*. Aug 1987;25(4):389-400. doi:10.1002/ajh.2830250405
457. Falusi AG, Esan GJ, Ayyub H, Higgs DR. Alpha-thalassaemia in Nigeria: its interaction with sickle-cell disease. *Eur J Haematol*. Apr 1987;38(4):370-5. doi:10.1111/j.1600-0609.1987.tb00013.x
458. Dover GJ, Chang VT, Boyer SH, Serjeant GR, Antonarakis S, Higgs DR. The cellular basis for different fetal hemoglobin levels among sickle cell individuals with two, three, and four alpha-globin genes. *Blood*. Jan 1987;69(1):341-4.
459. Williams S, Maude GH, Serjeant GR. Clinical presentation of sickle cell-hemoglobin C disease. *J Pediatr*. Oct 1986;109(4):586-9. doi:10.1016/s0022-3476(86)80217-7
460. Billett HH, Kim K, Fabry ME, Nagel RL. The percentage of dense red cells does not predict incidence of sickle cell painful crisis. *Blood*. Jul 1986;68(1):301-3.
461. Milner PF, Garbutt GJ, Nolan-Davis LV, Jonah F, Wilson LB, Wilson JT. The effect of Hb F and alpha-thalassemia on the red cell indices in sickle cell anemia. *Am J Hematol*. Apr 1986;21(4):383-95. doi:10.1002/ajh.2830210407
462. Stevens MC, Maude GH, Beckford M, et al. Alpha thalassemia and the hematology of homozygous sickle cell disease in childhood. *Blood*. Feb 1986;67(2):411-4.
463. Bainbridge R, Higgs DR, Maude GH, Serjeant GR. Clinical presentation of homozygous sickle cell disease. *J Pediatr*. Jun 1985;106(6):881-5. doi:10.1016/s0022-3476(85)80230-4
464. Noguchi CT, Dover GJ, Rodgers GP, et al. Alpha thalassemia changes erythrocyte heterogeneity in sickle cell disease. *J Clin Invest*. May 1985;75(5):1632-7. doi:10.1172/JCI111870
465. Nagel RL, Fabry ME, Pagnier J, et al. Hematologically and genetically distinct forms of sickle cell anemia in Africa. The Senegal type and the Benin type. *N Engl J Med*. Apr 4 1985;312(14):880-4. doi:10.1056/NEJM198504043121403
466. Labie D, Pagnier J, Lapoumeroulie C, et al. Common haplotype dependency of high G gamma-globin gene expression and high Hb F levels in beta-thalassemia and sickle cell anemia patients. *Proc Natl Acad Sci U S A*. Apr 1985;82(7):2111-4. doi:10.1073/pnas.82.7.2111
467. el-Hazmi MA. Clinical manifestation and laboratory findings of sickle cell anaemia in association with alpha-thalassaemia in Saudi Arabia. *Acta Haematol*. 1985;74(3):155-60. doi:10.1159/000206194
468. Steinberg MH, Coleman MB, Adams JG, Rosenstock W. Interaction between HBS-beta-o-thalassemia and alpha-thalassemia. *Am J Med Sci*. Dec 1984;288(5):195-9. doi:10.1097/00000441-198412000-00001
469. Fabry ME, Mears JG, Patel P, et al. Dense cells in sickle cell anemia: the effects of gene interaction. *Blood*. Nov 1984;64(5):1042-6.
470. Steinberg MH, Rosenstock W, Coleman MB, et al. Effects of thalassemia and microcytosis on the hematologic and vasoocclusive severity of sickle cell anemia. *Blood*. Jun 1984;63(6):1353-60.
471. Embury SH, Clark MR, Monroy G, Mohandas N. Concurrent sickle cell anemia and alpha-thalassemia. Effect on pathological properties of sickle erythrocytes. *J Clin Invest*. Jan 1984;73(1):116-23. doi:10.1172/JCI111181
472. Rucknagel D, Ferrucci S, Whitten CF, et al. alpha-Thalassemia and HbF concentration in sickle cell anemia. *Prog Clin Biol Res*. 1984;165:103-20.
473. Higgs DR, Aldridge BE, Lamb J, et al. The interaction of alpha-thalassemia and homozygous sickle-cell disease. *N Engl J Med*. Jun 17 1982;306(24):1441-6. doi:10.1056/NEJM198206173062402
474. Embury SH, Dozy AM, Miller J, et al. Concurrent sickle-cell anemia and alpha-thalassemia: effect on severity of anemia. *N Engl J Med*. Feb 4 1982;306(5):270-4. doi:10.1056/NEJM198202043060504
475. Altay C, Gravely ME, Joseph BR, Williams DF. alpha-thalassemia-2 and the variability of hematological values in children with sickle cell anemia. *Pediatr Res*. Aug 1981;15(8):1093-6. doi:10.1203/00006450-198108000-00004

476. Bauer DE, Kamran SC, Lessard S, et al. An erythroid enhancer of BCL11A subject to genetic variation determines fetal hemoglobin level. Article. *Science*. Oct 11 2013;342(6155):253-7. doi:10.1126/science.1242088
477. Chaturvedi S, Bhatnagar P, Bean CJ, et al. Genome-wide association study to identify variants associated with acute severe vaso-occlusive pain in sickle cell anemia. Letter. *Blood*. Aug 3 2017;130(5):686-688. doi:10.1182/blood-2017-02-769661
478. Lee N, Makani J, Tluway F, et al. Decreased Hepcidin Levels Are Associated with Low Steady-state Hemoglobin in Children With Sickle Cell Disease in Tanzania. Article. *EBioMedicine*. Aug 2018;34:158-164. doi:10.1016/j.ebiom.2018.07.024
479. Adorno EV, Zanette Â, Lyra I, Seixas MO, Reis MG, Gonçalves MS. Clinical and molecular characteristics of sickle cell anemia in the northeast of Brazil. Article. *Genet Mol Biol*. 2008;31(3):621-625. doi:10.1590/s1415-47572008000400003
480. Marouf R, Gupta R, Haider MZ, Al-Wazzan H, Adekile AD. Avascular necrosis of the femoral head in adult Kuwaiti sickle cell disease patients. Article. *Acta Haematol*. 2003;110(1):11-5. doi:10.1159/000072406
481. Darshana T, Bandara D, Nawarathne U, et al. Sickle cell disease in Sri Lanka: clinical and molecular basis and the unanswered questions about disease severity. Article. *Orphanet J Rare Dis*. Jul 6 2020;15(1):177. 177. doi:10.1186/s13023-020-01458-w
482. Costa FF, Tavella MH, Zago MA. DELETION TYPE ALPHA-THALASSEMIA AMONG BRAZILIAN PATIENTS WITH SICKLE-CELL ANEMIA. Article. *Rev Bras Genet*. Sep 1989;12(3):605-611.
483. Serjeant GR, Chin N, Asnani MR, et al. Causes of death and early life determinants of survival in homozygous sickle cell disease: The Jamaican cohort study from birth. Article. *PLoS One*. Mar 2018;13(3):e0192710. e0192710. doi:10.1371/journal.pone.0192710
484. Condon PI, Marsh RJ, Maude GH, Higgs DR, Weatherall DJ, Serjeant GR. Alpha thalassaemia and the macular vasculature in homozygous sickle cell disease. Article. *Br J Ophthalmol*. Nov 1983;67(11):779-81. doi:10.1136/bjo.67.11.779
485. Serjeant BE, Mason KP, Kenny MW, et al. Effect of alpha thalassaemia on the rheology of homozygous sickle cell disease. Article. *Br J Haematol*. Nov 1983;55(3):479-86. doi:10.1111/j.1365-2141.1983.tb02163.x
486. Torres LS, Okumura JV, Silva DG, et al. Inflammation in Sickle Cell Disease: Differential and Down-Expressed Plasma Levels of Annexin A1 Protein. Article. *PLoS One*. Nov 2016;11(11):e0165833. e0165833. doi:10.1371/journal.pone.0165833
487. Adekile AD, Haider MZ, Serebour F, Al-Zaabi K, Tuli M. Serum Immunoglobulins and IgG Subclasses in SS Patients with Saudi Arabia/India Haplotype. Article. *Med Princ Pract*. Jul-Sep 1999;8(3):183-188. doi:10.1159/000026090
488. Vyas P, Higgs DR, Weatherall DJ, Dunn D, Serjeant BE, Serjeant GR. The interaction of alpha thalassaemia and sickle cell-beta zero thalassaemia. Article. *Br J Haematol*. Dec 1988;70(4):449-54. doi:10.1111/j.1365-2141.1988.tb02515.x
489. Cumming V, King L, Fraser R, Serjeant G, Reid M. Venous incompetence, poverty and lactate dehydrogenase in Jamaica are important predictors of leg ulceration in sickle cell anaemia. Article. *Br J Haematol*. Jul 2008;142(1):119-25. doi:10.1111/j.1365-2141.2008.07115.x
490. Dossou-Yovo OP, Zaccaria I, Benkerrou M, et al. Effects of RANTES and MBL2 gene polymorphisms in sickle cell disease clinical outcomes: association of the g.In1.1T>C RANTES variant with protection against infections. Article. *Am J Hematol*. Jun 2009;84(6):378-80. doi:10.1002/ajh.21411
491. Rahimi Z, Vaisi-Raygani A, Merat A, Haghshenass M, Rezaei M. Level of hemoglobin F and Gγ gene expression in sickle cell disease and their association with haplotype and XmnI polymorphic site in south of Iran. Article. *Iranian Journal of Medical Sciences*. 2007;32(4):234-239.
492. Adekile AD, Owunwanne A, Al-Za'abi K, Haider MZ, Tuli M, Al-Mohannadi S. Temporal sequence of splenic dysfunction in sickle cell disease. Article. *Am J Hematol*. Jan 2002;69(1):23-7. doi:10.1002/ajh.10010
493. Hoppe C, Cheng S, Grow M, et al. A novel multilocus genotyping assay to identify genetic predictors of stroke in sickle cell anaemia. Article. *Br J Haematol*. Sep 2001;114(3):718-20. doi:10.1046/j.1365-2141.2001.02997.x

© 2023 Kirkham JK et al. *JAMA Network Open*.

494. Al-Lamki Z, Wali YA, Shah W, Zachariah M, Rafique B, Ahmed S. Natural history of sickle hemoglobinopathies in Omani children. Article. *International Journal of Pediatric Hematology/Oncology*. 2000;7(2):101-107.
495. Platt OS, Brambilla DJ, Rosse WF, et al. Mortality in sickle cell disease. Life expectancy and risk factors for early death. Article. *N Engl J Med*. Jun 9 1994;330(23):1639-44. doi:10.1056/NEJM199406093302303
496. el-Hazmi MA, al-Swailem AR, Bahakim HM, al-Faleh FZ, Warsy AS. Effect of alpha thalassaemia, G-6-PD deficiency and Hb F on the nature of sickle cell anaemia in south-western Saudi Arabia. Article. *Trop Geogr Med*. Jul 1990;42(3):241-7.
497. el-Hazmi MA, Bahakim HM, al-Swailem AM, Warsy AS. The features of sickle cell disease in Saudi children. Article. *J Trop Pediatr*. Aug 1990;36(4):148-55. doi:10.1093/tropej/36.4.148
498. Falusi AG, Esan GJ. Foetal haemoglobin levels in sickle cell anaemia in Nigerians. Article. *Afr J Med Med Sci*. Jun 1989;18(2):145-9.
499. de Ceulaer K, Higgs DR, Weatherall DJ, Hayes RJ, Serjeant BE, Serjeant GR. alpha-Thalassemia reduces the hemolytic rate in homozygous sickle-cell disease. Letter. *N Engl J Med*. Jul 21 1983;309(3):189-90. doi:10.1056/NEJM198307213090320
500. Steinberg MH, Coleman MB, Adams JG, Platica O, Gillette P, Rieder RF. The effects of alpha-thalassaemia in HbSC disease. Article. *Br J Haematol*. Nov 1983;55(3):487-92. doi:10.1111/j.1365-2141.1983.tb02164.x
501. Bernardo VS, Torres FF, Chaves NA, Okumura JV, da Silva DGH, Bonini-Domingos CR. Relationship of polymorphism rs3800231 in FOXO3 gene and clinical severity with oxidative stress markers in sickle cell disease. Article. *Meta Gene*. 2020;24100660. doi:10.1016/j.mgene.2020.100660
502. Belisario AR, de Almeida JA, Mendes FG, et al. Prevalence and risk factors for albuminuria and glomerular hyperfiltration in a large cohort of children with sickle cell anemia. Letter. *Am J Hematol*. May 2020;95(5):E125-E128. doi:10.1002/ajh.25763
503. Patel R, Kang S, Valeshabad AK, et al. Kidney ultrasound findings according to kidney function in sickle cell anemia. Letter. *Am J Hematol*. Nov 2019;94(11):E288-E291. doi:10.1002/ajh.25602
504. Uyoga S, Macharia AW, Mochamah G, et al. The epidemiology of sickle cell disease in children recruited in infancy in Kilifi, Kenya: a prospective cohort study. Article. *Lancet Glob Health*. Oct 2019;7(10):e1458-e1466. doi:10.1016/S2214-109X(19)30328-6
505. Amle DB, Patnayak RL, Verma V, et al. VEGF Promoter Region 18-bp Insertion-Deletion Polymorphism in Sickle Cell Disease Patients with Microalbuminuria: A Pilot Study. Article. *Indian J Hematol Blood Transfus*. Apr 2019;35(2):278-283. doi:10.1007/s12288-018-1018-x
506. Hassan FM, Alzahrani FM. Cytokines TNF-Alpha and IL-8 Gene Polymorphisms in Sickle Cell Anaemia Patients under Hydroxyurea Treatment. Article. *Journal of Clinical and Diagnostic Research*. 2018;12(6):EC14-EC17. doi:10.7860/jcdr/2018/35740.11681
507. Laurentino MR, Barbosa MC, Santos TEJ, Perdigão ACB, Araújo FMC, Lemes RPG. Analysis of BCL11A gene polymorphisms and hemolysis parameters in patients with: sickle-cell disease. Article. *Jornal Brasileiro de Patologia e Medicina Laboratorial*. 2018;54(3):132-137. doi:10.5935/1676-2444.20180025
508. Vasseur C, Domingues-Hamdi E, Pakdaman S, et al. Elevated soluble alpha-hemoglobin pool in sickle cell anemia. Letter. *Am J Hematol*. Oct 2017;92(10):E593-E595. doi:10.1002/ajh.24835
509. Howell S, Marshall K, Reid M, McFarlane-Anderson N, McKenzie C. A cross-sectional clinic-based study exploring whether variants within genes coding for enzymes of the transmethylation and trans-sulphuration pathways are associated with inter-individual phenotypic variation in sickle cell anaemia. Article. *West Indian Medical Journal*. 2017;66(4):510-517. doi:10.7727/wimj.2016.205
510. Medeiros FS, Mendonca TF, Lopes KAM, et al. Combined genotypes of the MBL2 gene related to low mannose-binding lectin levels are associated with vaso-occlusive events in children with sickle cell anemia. Article. *Genet Mol Biol*. Jul-Sep 2017;40(3):600-603. doi:10.1590/1678-4685-GMB-2016-0161
511. Watanabe AM, Pianovski MAD, Lenzi L, Cat R. The frequency of βS-globin haplotypes in the state of Paraná, Brazil, and clinical: manifestations of sickle cell anemia. Article. *Jornal Brasileiro de Patologia e Medicina Laboratorial*. 2017;53(1):24-30. doi:10.5935/1676-2444.20170007

© 2023 Kirkham JK et al. *JAMA Network Open*.

512. Figueiredo CVB, Carvalho MOS, Santiago RP, et al. Leptin − 2548 G > A gene polymorphism is associated with lipids metabolism and TGF-β alteration in sickle cell disease. Article. *Meta Gene*. 2016;10:27-31. doi:10.1016/j.mgene.2016.10.001
513. Ben Sassi M, Chaouch L, Kalai M, et al. Association of rs1319868, rs1567811 and rs8041224 of IGF1R gene with infection among sickle cell anemia Tunisian patients. Article. *Acta Haematologica Polonica*. 2016;47(4):242-247. doi:10.1016/j.achaem.2016.10.004
514. Purohit P, Patel S, Mohanty PK, Das P, Panigrahi J. Fetal Hemoglobin Modifies the Disease Manifestation of Severe Plasmodium Falciparum Malaria in Adult Patients with Sickle Cell Anemia. Letter. *Mediterr J Hematol Infect Dis*. 2016;8(1):e2016055. e2016055. doi:10.4084/MJHID.2016.055
515. Leal AS, Martins PRJ, Balarin MAS, Pereira GA, Resende GAD. Haplotypes βs-globin and its clinical-haematological correlation in patients with sickle-cell anemia in Triângulo Mineiro, Minas Gerais, Brazil. Article. *Jornal Brasileiro de Patologia e Medicina Laboratorial*. 2016;52(1):5-10. doi:10.5935/1676-2444.20160001
516. Araujo NB, Domingos IF, Medeiros FS, et al. Lack of association between the Duffy antigen receptor for chemokines (DARC) expression and clinical outcome of children with sickle cell anemia. Letter. *Immunol Lett*. Aug 2015;166(2):140-2. doi:10.1016/j.imlet.2015.05.015
517. Leila C, Miniar K, Dorra C, et al. Gilbert syndrome acts as a risk factor of developing gallstone among β hemoglobinopathy Tunisian patients. Article. *Tunisie Medicale*. 2015;93(4):237-241.
518. Lustosa Souza CR, Azevedo Shimmoto MM, Vicari P, Mecabo G, Arruda MM, Figueiredo MS. Klotho gene polymorphisms and their association with sickle cell disease phenotypes. Letter. *Rev Bras Hematol Hemoter*. Jul-Aug 2015;37(4):275-6. doi:10.1016/j.bjhh.2015.02.009
519. Rabab A. GSTM1 and GSTT1 Polymorphism in Egyptian Sickle Cell Anemia Patients. Article. *International Journal of Hematology and Oncology*. 2013;23(4):269-275. doi:10.4999/uhod.13025
520. Kangne HK, Jijina FF, Italia YM, et al. The Fc receptor polymorphisms and expression of neutrophil activation markers in patients with sickle cell disease from Western India. Article. *Biomed Res Int*. 2013;2013:457656. 457656. doi:10.1155/2013/457656
521. Drasar ER, Menzel S, Fulford T, Thein SL. The effect of Duffy antigen receptor for chemokines on severity in sickle cell disease. Letter. *Haematologica*. Aug 2013;98(8):e87-9. doi:10.3324/haematol.2013.089243
522. Lemonne N, Lamarre Y, Romana M, et al. Does increased red blood cell deformability raise the risk for osteonecrosis in sickle cell anemia? Letter. *Blood*. Apr 11 2013;121(15):3054-6. doi:10.1182/blood-2013-01-480277
523. El Deen MAK, Khorshied MM, El Sadani ZA, Amrousy YM, Galal NM. Mannose-binding lectin (MBL2) gene polymorphism in sickle cell anemia: an Egyptian study. Article. *Comparative Clinical Pathology*. 2012;22(3):387-394. doi:10.1007/s00580-012-1420-y
524. Pandey S, Ranjan R, Firdos A, et al. Relation between the uridine diphosphate glucuronosyltransferase 1A1 polymorphism and the bilirubin levels in sickle cell disease. Article. *Journal of Clinical and Diagnostic Research*. 2012;6(5):821-824.
525. Pandey S, Pandey S, Mishra RM, Saxena R. Modulating Effect of the -158 gamma (C-->T) Xmn1 Polymorphism in Indian Sickle Cell Patients. Article. *Mediterr J Hematol Infect Dis*. 2012;4(1):e2012001. e2012001. doi:10.4084/MJHID.2012.001
526. Heitzer AM, Longoria J, Rampersaud E, et al. Fetal hemoglobin modulates neurocognitive performance in sickle cell anemia(☆,☆☆). *Curr Res Transl Med*. Jul 2022;70(3):103335. doi:10.1016/j.retram.2022.103335
527. de Freitas Dutra V, Leal VNC, Fernandes FP, Souza CRL, Figueiredo MS, Pontillo A. Genetic contribution and functional impairment of inflammasome in sickle cell disease. *Cytokine*. Jan 2022;149:155717. doi:10.1016/j.cyto.2021.155717
528. Garrett ME, Soldano KL, Erwin KN, et al. Genome-wide meta-analysis identifies new candidate genes for sickle cell disease nephropathy. *Blood Adv*. Nov 18 2022;doi:10.1182/bloodadvances.2022007451
529. Nardo-Marino A, Glenthøj A, Brewin JN, et al. The significance of spleen size in children with sickle cell anemia. *Am J Hematol*. Dec 2022;97(12):1520-1528. doi:10.1002/ajh.26703
530. Wang X, Gardner K, Tegegn MB, et al. Genetic variants of PKLR are associated with acute pain in sickle cell disease. *Blood Adv*. Jun 14 2022;6(11):3535-3540. doi:10.1182/bloodadvances.2021006668

© 2023 Kirkham JK et al. *JAMA Network Open*.

531. Adebayo OC, Betukumesu DK, Nkoy AB, et al. Clinical and genetic factors are associated with kidney complications in African children with sickle cell anaemia. *Br J Haematol*. Jan 2022;196(1):204-214. doi:10.1111/bjh.17832
532. Pincez T, Lo KS, D'Orengiani A, Garrett, M. E., et al. Variation and impact of polygenic hematologic traits in monogenic sickle cell disease. *Haematologica*. Mar 1 2023;108(3):870-881. doi:10.3324/haematol.2022.281180
533. Walker TM, Hambleton IR, Mason KP, Serjeant G. Spleen size in homozygous sickle cell disease: trends in a birth cohort using ultrasound. *Br J Radiol*. Dec 1 2022;95(1140):20220634. doi:10.1259/bjr.20220634
534. Ibrahim NS, Makhlouf MM, Shahin GH, Elghamrawy MK, Hussein NM. The impact of MCP1-2518A/G and CCR2-V64I genetic polymorphisms in Egyptian sickle cell disease patients. *Exp Mol Pathol*. Oct 2022;128:104834. doi:10.1016/j.yexmp.2022.104834
535. Allard P, Alhaj N, Lobitz S, et al. Genetic modifiers of fetal hemoglobin affect the course of sickle cell disease in patients treated with hydroxyurea. *Haematologica*. Jul 1 2022;107(7):1577-1588. doi:10.3324/haematol.2021.278952
536. Liyanage JSS, Estepp JH, Srivastava K, Li Y, Mori M, Kang G. GMEPS: a fast and efficient likelihood approach for genome-wide mediation analysis under extreme phenotype sequencing. *Stat Appl Genet Mol Biol*. Mar 11 2022;21(1)doi:10.1515/sagmb-2021-0071
537. Rizo-de la Torre LC, Borrayo-López FJ, Perea-Díaz FJ, et al. Fetal hemoglobin regulating genetic variants identified in homozygous (HbSS) and heterozygous (HbSA) subjects from South Mexico. *J Trop Pediatr*. Aug 4 2022;68(5)doi:10.1093/tropej/fmac073
538. Boisson C, Renoux C, Nader E, et al. Comparisons of oxygen gradient ektacytometry parameters between sickle cell patients with or without α-thalassaemia. *Br J Haematol*. Nov 2021;195(4):629-633. doi:10.1111/bjh.17777
539. Osunkalu VO, Ogbenna AA, Davies NO, et al. Assessment of MTR Rs1805087 SNP as Possible Modifier of Sickle Cell Disease Severity in a Nigerian Population. *West Afr J Med*. Nov 30 2022;39(11):1198-1204.
540. Belvitch P, Casanova N, Sun X, et al. A cortactin CTTN coding SNP contributes to lung vascular permeability and inflammatory disease severity in African descent subjects. *Transl Res*. Jun 2022;244:56-74. doi:10.1016/j.trsl.2022.02.002
541. Germano I, Santos B, Delgadinho M, et al. Genetic modulation of anemia severity, hemolysis level, and hospitalization rate in Angolan children with Sickle Cell Anemia. *Mol Biol Rep*. Nov 2022;49(11):10347-10356. doi:10.1007/s11033-022-07831-1
542. Hariharan P, Gorivale M, Sawant P, Mehta P, Nadkarni A. Significance of genetic modifiers of hemoglobinopathies leading towards precision medicine. *Sci Rep*. Oct 22 2021;11(1):20906. doi:10.1038/s41598-021-00169-x
543. Prohaska CC, Zhang X, Schwantes-An TL, et al. RASA3 is a candidate gene in sickle cell disease-associated pulmonary hypertension and pulmonary arterial hypertension. *Pulm Circ*. Apr 2023;13(2):e12227. doi:10.1002/pul2.12227
544. Powell-Roach KL, Yao Y, Wallace MR, et al. HUMAN STUDY COMT and DRD3 haplotype-associated pain intensity and acute care utilization in adult sickle cell disease. *Exp Biol Med (Maywood)*. Sep 2022;247(17):1601-1608. doi:10.1177/15353702221080716
545. Ndour EHM, Mnika K, Guèye Tall F, et al. Effects of Senegal haplotype (Xmn1-rs7412844), alpha-thalassemia (3.7kb HBA1/HBA2 deletion), NPRL3-rs11248850 and BCL11A-rs4671393 variants on sickle cell nephropathy. *Int J Biochem Mol Biol*. 2022;13(2):5-16.
546. Menezes JF, Carvalho MOS, Rocha LC, et al. Role of paraoxonase 1 activity and PON1 gene polymorphisms in sickle cell disease. *Sci Rep*. May 3 2023;13(1):7215. doi:10.1038/s41598-023-34396-1
547. Sales RR, Nogueira BL, Belisário AR, et al. Fetal hemoglobin-boosting haplotypes of BCL11A gene and HBS1L-MYB intergenic region in the prediction of clinical and hematological outcomes in a cohort of children with sickle cell anemia. *J Hum Genet*. Dec 2022;67(12):701-709. doi:10.1038/s10038-022-01079-0
548. Manu GP, Segbefia C, N'Guessan B B, Coffie SA, Adjei GO. Association Between Selected Single Nucleotide Polymorphisms in Globin and Related Genes and Response to Hydroxyurea Therapy in Ghanaian Children with Sickle Cell Disease. *Pharmgenomics Pers Med*. 2022;15:205-214. doi:10.2147/pgpm.S351599

© 2023 Kirkham JK et al. *JAMA Network Open*.

549. Brewin JN, Nardo-Marino A, Stuart-Smith S, et al. The pleiotropic effects of α-thalassemia on HbSS and HbSC sickle cell disease: Reduced erythrocyte cation co-transport activity, serum erythropoietin, and transfusion burden, do not translate into increased survival. *Am J Hematol*. Oct 2022;97(10):1275-1285. doi:10.1002/ajh.26652
550. Powell-Roach KL, Yao Y, Cao X, et al. Analysis of AVPR1A, thermal and pressure pain thresholds, and stress in sickle cell disease. *Front Pain Res (Lausanne)*. 2022;3:1060245. doi:10.3389/fpain.2022.1060245
551. Hamadi A, Mir R, Mahzari A, et al. Molecular Determination of Vascular Endothelial Growth Factor, miRNA-423 Gene Abnormalities by Utilizing ARMS-PCR and Their Association with Fetal Hemoglobin Expression in the Patients with Sickle Cell Disease. *Curr Issues Mol Biol*. Jun 1 2022;44(6):2569-2582. doi:10.3390/cimb44060175
552. Bernaudin F, Arnaud C, Kamdem A, et al. Incidence, kinetics, and risk factors for intra- and extracranial cerebral arteriopathies in a newborn sickle cell disease cohort early assessed by transcranial and cervical color Doppler ultrasound. *Front Neurol*. 2022;13:846596. doi:10.3389/fneur.2022.846596
553. Duru AN, Ocheni S, Ibegbulam O, Okpala I. Plasma Concentration of 12-Hydroxyeicosatetraenoic Acid, Single Nucleotide Polymorphisms of 12-Lipooxygenase Gene and Vaso-Occlusion in Sickle Cell Disease. *Front Genome Ed*. 2021;3:722190. doi:10.3389/fgeed.2021.722190
554. Dosunmu-Ogunbi A, Yuan S, Reynolds M, et al. SOD2 V16A amplifies vascular dysfunction in sickle cell patients by curtailing mitochondria complex IV activity. *Blood*. Mar 17 2022;139(11):1760-1765. doi:10.1182/blood.2021013350
555. Kumkhaek C, Kim C, Kurban G, et al. Single nucleotide polymorphisms in SAR1A coding regions in sickle cell disease and their potential miRNA binding sites. *EJHaem*. Nov 2022;3(4):1438-1441. doi:10.1002/jha2.542
556. Dehury S, Mohanty PK, Patel S, et al. Profiling of 35 Cases of Hb S/Hb E (HBB: c.20A>T/HBB: c.79G>a), Disease and Association with α-Thalassemia and β-Globin Gene Cluster Haplotypes from Odisha, India. *Hemoglobin*. Nov 2021;45(6):380-386. doi:10.1080/03630269.2021.1965618
557. Hassab H, Hanafi M, Elbeheiry A, Hassan M, Chazli YE. Does TGFBR3 Polymorphism Increase the Risk of Silent Cerebral Infarction in Egyptian Children with Sickle Cell Disease? *Indian J Pediatr*. Feb 2023;90(2):146-152. doi:10.1007/s12098-022-04181-5
558. Knisely MR, Yang Q, Stauffer N, et al. Evaluating Associations between Average Pain Intensity and Genetic Variation in People with Sickle Cell Disease: An Exploratory Study. *Pain Manag Nurs*. Feb 2023;24(1):12-18. doi:10.1016/j.pmn.2022.08.002
559. do Kleyton Palmeira Ó, da Silva Freire, A. K., de Nóbrega DN, Dos Santos Souza R, et al. Polymorphisms and gene expression of metalloproteinases and their inhibitors associated with cerebral ischemic stroke in young patients with sickle cell anemia. *Mol Biol Rep*. Apr 2023;50(4):3341-3353. doi:10.1007/s11033-023-08262-2
560. Purohit P, Mohanty PK, Panigrahi J, Das K, Patel S. Effect of α(+) Thalassemia on the Severity of Plasmodium falciparum Malaria in Different Sickle Cell Genotypes in Indian Adults: A Hospital-Based Study. *Hemoglobin*. Jan 2023;47(1):11-18. doi:10.1080/03630269.2023.2168201
561. Earley EJ, Kelly S, Fang F, et al. Genome-wide association study of early ischaemic stroke risk in Brazilian individuals with sickle cell disease implicates ADAMTS2 and CDK18 and uncovers novel loci. *Br J Haematol*. Apr 2023;201(2):343-352. doi:10.1111/bjh.18637
562. Olupot-Olupot P, Tomlinson G, Williams TN, et al. Hydroxyurea treatment is associated with lower malaria incidence in children with sickle cell anemia in sub-Saharan Africa. *Blood*. Mar 23 2023;141(12):1402-1410. doi:10.1182/blood.2022017051
563. Cappelli B, Scigliuolo GM, Boukouaci W, et al. Impact of the human leucocyte antigen (HLA)-B leader peptide dimorphism and HLA-A expression on outcomes of stem cell transplantation for sickle cell disease. *Br J Haematol*. Oct 2021;195(2):e128-e131. doi:10.1111/bjh.17665
564. Liyanage JSS, Estepp JH, Srivastava K, et al. A Versatile and Efficient Novel Approach for Mendelian Randomization Analysis with Application to Assess the Causal Effect of Fetal Hemoglobin on Anemia in Sickle Cell Anemia. Article. *Mathematics*. Oct 2022;10(20)3743. doi:10.3390/math10203743
565. Khorshied MM, Shaheen IA, Selim YMM, Elshahawy AO, Youssry I. Impact of Superoxide Dismutase Genetic Polymorphism (SOD2 Val16Ala) and Superoxide Dismutase Level on Disease

© 2023 Kirkham JK et al. *JAMA Network Open*.

Severity in a Cohort of Egyptian Sickle Cell Disease Patients. Article. *Mediterranean Journal of Hematology and Infectious Diseases*. May 2022;14e2022037. doi:10.4084/mjhid.2022.037
566. Nardo-Marino A, Petersen J, Brewin JN, et al. Oxygen gradient ektacytometry does not predict pain in children with sickle cell anaemia. Article. *British Journal of Haematology*. Jun 2022;197(5):609-617. doi:10.1111/bjh.17975
567. Martins JO, Pagani F, Dezan MR, et al. Impact of HLA-G +3142C>G on the development of antibodies to blood group systems other than the Rh and Kell among sensitized patients with sickle cell disease. Article. *Transfusion and Apheresis Science*. 2022;61(5)103447. doi:10.1016/j.transci.2022.103447
568. da Costa-Júnior DA, Santos APP, da Silva CM, Velloso-Rodrigues C. Growth Hormone/Insulin-like Growth Factor 1 Axis Associated with Modifier Factors in Children with Sickle Cell Anemia. Article. *Endocrine, Metabolic and Immune Disorders - Drug Targets*. 2022;22(9):954-962. doi:10.2174/1871530322666220303164029
569. Kengne Fotsing CB, Pieme CA, Biapa Nya PC, et al. Relation between haptoglobin polymorphism and oxidative stress status, lipid profile, and cardiovascular risk in sickle cell anemia patients. Article. *Health Science Reports*. 2022;5(1)e465. doi:10.1002/hsr2.465
570. Sokkar MF, Kamal L, Salama N, Hamdy M. Thrombophilic mutations and risk of vascular complications in sickle cell disease. Article. *Gene Reports*. 2022;27101595. doi:10.1016/j.genrep.2022.101595
571. Lakkakula BVKS, Pattnaik S. Hbb gene cluster haplotype diversity in sickle cell anemia patients of chhattisgarh, india. Article. *Journal of Applied Biology and Biotechnology*. 2021;9(5):64-69. doi:10.7324/JABB.2021.9509
572. Smith WR, Penberthy LT, Bovbjerg VE, et al. Daily assessment of pain in adults with sickle cell disease. *Ann Intern Med*. Jan 15 2008;148(2):94-101. doi:10.7326/0003-4819-148-2-200801150-00004
573. Zaidi AU, Glaros AK, Lee S, et al. A systematic literature review of frequency of vaso-occlusive crises in sickle cell disease. *Orphanet J Rare Dis*. Nov 2 2021;16(1):460. doi:10.1186/s13023-021-02096-6
574. Piel FB, Jobanputra M, Gallagher M, Weber J, Laird SG, McGahan M. Co-morbidities and mortality in patients with sickle cell disease in England: A 10-year cohort analysis using hospital episodes statistics (HES) data. *Blood Cells Mol Dis*. Jul 2021;89:102567. doi:10.1016/j.bcmd.2021.102567
575. Idris IM, Abba A, Galadanci JA, et al. Men with sickle cell disease experience greater sexual dysfunction when compared with men without sickle cell disease. *Blood Adv*. Jul 28 2020;4(14):3277-3283. doi:10.1182/bloodadvances.2020002062
576. Brousse V, Elie C, Benkerrou M, et al. Acute splenic sequestration crisis in sickle cell disease: cohort study of 190 paediatric patients. *Br J Haematol*. Mar 2012;156(5):643-8. doi:10.1111/j.1365-2141.2011.08999.x
577. Rosse WF, Gallagher D, Kinney TR, et al. Transfusion and alloimmunization in sickle cell disease. The Cooperative Study of Sickle Cell Disease. *Blood*. Oct 1 1990;76(7):1431-7.
578. Kassim AA, Pruthi S, Day M, et al. Silent cerebral infarcts and cerebral aneurysms are prevalent in adults with sickle cell anemia. *Blood*. Apr 21 2016;127(16):2038-40. doi:10.1182/blood-2016-01-694562
579. Lachman BS, Lazerson J, Starshak RJ, Vaughters FM, Werlin SL. The prevalence of cholelithiasis in sickle cell disease as diagnosed by ultrasound and cholecystography. *Pediatrics*. Nov 1979;64(5):601-3.
580. Niss O, Quinn CT, Lane A, et al. Cardiomyopathy With Restrictive Physiology in Sickle Cell Disease. *JACC Cardiovasc Imaging*. Mar 2016;9(3):243-52. doi:10.1016/j.jcmg.2015.05.013
581. Bohm N, Toussaint B, Sarratt S, Vaughan L, Duckett A. Assessment of Iron Overload and Chelation Therapy in Adult Inpatients with Sickle Cell Disease. *International Archives of Clinical Pharmacology*. 2018;4(1)doi:10.23937/2572-3987.1510014
582. Nawaiseh M, Roto A, Nawaiseh Y, et al. Risk factors associated with sickle cell retinopathy: findings from the Cooperative Study of Sickle Cell Disease. *Int J Retina Vitreous*. Sep 22 2022;8(1):68. doi:10.1186/s40942-022-00419-8
583. Rogers ZR, Wang WC, Luo Z, et al. Biomarkers of splenic function in infants with sickle cell anemia: baseline data from the BABY HUG Trial. *Blood*. Mar 3 2011;117(9):2614-7. doi:10.1182/blood-2010-04-278747

© 2023 Kirkham JK et al. *JAMA Network Open*.

584. Little J, Higgins JP, Ioannidis JP, et al. STrengthening the REporting of Genetic Association Studies (STREGA): an extension of the STROBE statement. *PLoS Med*. Feb 3 2009;6(2):e22. doi:10.1371/journal.pmed.1000022
585. Vandenbroucke JP, von Elm E, Altman DG, et al. Strengthening the Reporting of Observational Studies in Epidemiology (STROBE): explanation and elaboration. *PLoS Med*. Oct 16 2007;4(10):e297. doi:10.1371/journal.pmed.0040297
586. Willer CJ, Li Y, Abecasis GR. METAL: fast and efficient meta-analysis of genomewide association scans. *Bioinformatics*. Sep 1 2010;26(17):2190-1. doi:10.1093/bioinformatics/btq340
587. Wu T, Hu E, Xu S, et al. clusterProfiler 4.0: A universal enrichment tool for interpreting omics data. *Innovation (Camb)*. Aug 28 2021;2(3):100141. doi:10.1016/j.xinn.2021.100141
588. Yu G, He QY. ReactomePA: an R/Bioconductor package for reactome pathway analysis and visualization. *Mol Biosyst*. Feb 2016;12(2):477-9. doi:10.1039/c5mb00663e
589. The Gene Ontology Consortium. The Gene Ontology Resource: 20 years and still GOing strong. *Nucleic Acids Res*. Jan 8 2019;47(D1):D330-D338. doi:10.1093/nar/gky1055
590. Gillespie M, Jassal B, Stephan R, et al. The reactome pathway knowledgebase 2022. *Nucleic Acids Res*. Jan 7 2022;50(D1):D687-D692. doi:10.1093/nar/gkab1028
591. Benjamini Y, Hochberg Y. Controlling the False Discovery Rate: A Practical and Powerful Approach to Multiple Testing. *Journal of the Royal Statistical Society: Series B (Methodological)*. 1995;57(1):289-300. doi:10.1111/j.2517-6161.1995.tb02031.x

© 2023 Kirkham JK et al. *JAMA Network Open*.
