# Supplementary material for: Genetic Variation and Sickle Cell Disease Severity: A Systematic Review and Meta-Analysis
Source: JAMA Netw Open. 2023 Oct 18;6(10):e2337484. doi: 10.1001/jamanetworkopen.2023.37484 (PMC10585422; doi:10.1001/jamanetworkopen.2023.37484)
Supplement: Supplement 3. — Data Sharing Statement [file jamanetwopen-e2337484-s003.pdf]

Data Sharing Statement

Kirkham. Genetic Variation and Sickle Cell Disease Severity. *JAMA Netw Open*. Published October 12, 2023. doi:10.1001/jamanetworkopen.2023.37484

Data
Data available: Yes
Data types: Data (not involving human participants)
How to access data: All extracted data used in all analyses and results presented in this manuscript are included in eTables 1-3.
When available: With publication

Supporting Documents
Document types: None

Additional Information
Who can access the data: Anyone
Types of analyses: All
Mechanisms of data availability: All
